# Establishment of reliable identification algorithms for acute heart failure or acute exacerbation of chronic heart failure using clinical data from a medical information database network

**DOI:** 10.3389/fcvm.2025.1642323

**Published:** 2025-10-15

**Authors:** Ryusuke Inoue, Masaharu Nakayama, Hideki Ota, Naoki Nakamura, Susumu Fujii, Akira Ishii, Atsuko Saito, Takahiro Suzuki, Hiroko Nomura, Natsuko Goto, Shinya Watanabe, Hotaka Maruyama, Mayu Nozawa, Yoshiaki Uyama

**Affiliations:** ^1^ Medical Information Technology Center, Tohoku University Hospital, Sendai, Miyagi, Japan; ^2^ Department of Medical Informatics, Tohoku University Graduate School of Medicine, Sendai, Miyagi, Japan; ^3^ Division of Medical Informatics and Management, Chiba University Hospital, Chiba-shi, Chiba, Japan; ^4^ Tokushukai General Incorporated Association Osaka, Osaka, Japan; ^5^ Office of Pharmacovigilance I, Pharmaceuticals and Medical Devices Agency, Chiyoda-ku, Tokyo, Japan; ^6^ Center for Regulatory Science, Pharmaceuticals and Medical Devices Agency, Chiyoda-ku, Tokyo, Japan

**Keywords:** MID-NET®, heart failure, phenotyping, medical information database network, real world data

## Abstract

**Introduction:**

This study aimed to evaluate the validity of algorithms based on electronic health data in identifying cases of acute heart failure and acute exacerbation of chronic heart failure at multiple institutions using the Medical Information Database Network (MID-NET®) in Japan.

**Methods:**

Data were collected from March 8, 2021 to March 31, 2021, from the data source of three hospitals among the MID-NET® cooperating medical institutions. All Possible Cases were defined by combining ICD-10 codes related to acute heart failure and abnormal values of serum B-type natriuretic peptide (BNP) or N-terminal pro-brain natriuretic peptide (NT-proBNP). Eighteen algorithms were created using various data sources in MID-NET®, including electronic medical records, diagnostic procedure combination (DPC) data, and health insurance claims data. True cases were determined by reviewing medical records obtained independently by two experienced physicians.

**Results:**

The kappa coefficient among the three medical institutions was 0.94 (95% confidence interval: 0.90–0.98). Among the 18 algorithms, the highest positive predictive value (PPV) of the three medical institutions was 77.78% for Algorithm 8 which was constructed using ICD-10 codes in DPC disease data, moderate or high range of abnormal BNP (≥100 pg/mL) or NT-proBNP (≥400 pg/mL), and medications for acute heart failure. The highest sensitivity at 89.53% was observed for Algorithm 9. This algorithm was constructed using a combination of disease codes entered in electronic medical records, DPC, or health insurance claims data, abnormal BNP values in the moderate or high range (≥100 pg/mL), and medications for acute heart failure. However, its PPV was the lowest among 18 algorithms, generally reflecting the inverse relationship between PPV and sensitivity. The same tendency was seen in the sensitivity study. Cases with stable chronic heart failure, renal insufficiency, assessment for cardiac function, or severe circulatory failure inflated false-positive cases in this study.

**Conclusion:**

Validated algorithms for identifying acute heart failure and acute exacerbation of chronic heart failure were successfully established. Using these algorithms should facilitate more appropriate pharmacoepidemiological studies related to acute heart failure and contribute to better drug safety assessments based on real-world data in Japan.

## Introduction

Heart failure is one of the leading causes of death in most countries around the world, and the number of patients with the disease is anticipated to surge, particularly in aging populations (1, 2). Heart failure is associated with many pathologies, including ischemic heart disease, valvular heart disease, myocarditis, and drug-induced heart failure. Among these, drug-induced heart failure has garnered attention in the field of cardio-oncology as a condition often seen with anticancer medications (3), emphasizing the need for prompt detection and innovative treatment strategies. To detect drug-induced heart failure as early as possible, accurate identification of heart failure patients based on data from hospital information systems is essential. Furthermore, e-phenotyping has emerged as a valuable approach for depicting patient conditions and inferring disease progression from electronic medical records and other clinical information (4–6).

There have been several studies on algorithms to identify heart failure from data in hospital information systems (7, 8). However, these studies were conducted at single centers and were based on algorithms using unstructured data such as free text. Algorithms using structured data such as International Statistical Classification of Diseases and Related Health Problems 10th Revision (ICD-10) are less accurate for retrieval in those studies, however, standardized structured data is easier to collect data from hospital information systems at multiple centers. Furthermore, these reports were made outside of Japan, and it would be difficult to directly apply them to medical database research in Japan.

Similar attempts using insurance claims data have also been reported in Japan (9, 10). However, the target populations in these studies were hospitalized patients, and the validated algorithms did not incorporate laboratory test results.

In the present study, we aimed to develop algorithms based on electronic health data to identify acute heart failure or acute exacerbation of chronic heart failure (hereinafter, “acute heart failure”) using the Medical Information Database Network (MID-NET®) (11–13). The validated algorithms are expected to support appropriate database research and safety measures for acute heart failure.

## Methods

### Data source

MID-NET® is a medical information database operated by the Pharmaceuticals and Medical Devices Agency (PMDA) and was inistially established in collaboration with 23 hospitals from 10 healthcare organizations when the present study began (11–13).

Since the database was launched in April 2018, PMDA, pharmaceutical companies, and academic researchers have mainly utilized MID-NET® for post-marketing drug safety assessment. This database stores a different types of hospital information system (HIS) data including health insurance claims data, Diagnosis Procedure Combination (DPC) data, and electronic medical records, which are standardized based on the specifications of Standardized Structured Medical Information eXchange version 2 (SS-MIX2) (14). In SS-MIX2, a standardized storage from HIS, diagnostic order data, prescription or injection order/execution data, and results of laboratory data are recorded (14); and in DPC, a case-mix patient classification system linked with a lump-sum payment system for inpatients in acute care hospitals (15, 16), diagnoses are recorded with disease name and the ICD-10 codes. These diagnostic data are entered into specific fields such as the main diagnosis, diagnosis causing admission, the most and second most resource-consuming diagnoses, comorbidities present on admission, and complications during admission (16). In addition to this, ordering information of procedures and medications are also recorded in DPC data. Health insurance claims data provide information on the performed procedures and prescribed medications, such as the name of the disease, visit date, medication, laboratory tests and procedures (17). However, medical imaging data are not available in MID-NET®.

### Definitions

#### Study population

The target population was defined as the inpatients or outpatients that met the algorithm of All Possible Cases (APC) (referred to the “*Definition of APC”* in detail) in three hospitals (Hospitals A, B, and C) from March 8, 2021–March 31, 2021 (study period). The APC was assumed to be a population that included all patients with acute heart failure during the study period (18). After we extracted the cases that met the algorithm of APC from the database, more than 100 cases were randomly sampled from each hospital to form the study population for medical chart reviews. A feasibility study was conducted on 30 cases meeting the APC algorithm, since at least 100 true cases with acute heart failure should be included in the study population to ensure the width of the 95% confidence interval of PPV being ±10% or less (19). Out of the 30 cases included in the feasibility study sample, 12 were determined to be true cases at three hospitals. Based on these results, in order to ensure a total of 100 or more true cases at all medical institutions in this study, the number of cases selected as the study population to be judged was set at 100 or more at each medical institution. The study period required for each medical institution to extract at least 100 true cases was 24 days.

#### Conditions for algorithms based on electronic health data

We developed 18 algorithms for the identification of cases with acute heart failure using diagnosis codes related to acute heart failure, medications for the diseases, clinical procedures (transthoracic echocardiography), or laboratory test values [brain natriuretic peptide (BNP) or N-terminal pro-BNP (NT-proBNP)]. Table 1 outlines the conditions used for the algorithms. All codes and data fields used in this study are available in the Supplementary Table S1.

**Table 1:** Overview of the conditions used in the algorithms for the identification of the cases with acute heart failure in MID-NET®.

Condition-name	Conditions
Disease 1	Disease names related to acute heart failure in DPC (any of the six items^a^).
Disease 2	Disease names related to acute heart failure in DPC (any of the three items^b^).
Disease 3	Disease names related to acute heart failure (including suspected disease name) in SS-MIX2.
Disease 4	Disease names related to acute heart failure (not including suspected disease name) in SS-MIX2.
Disease 5	Disease names related to acute heart failure in the health insurance claims data.
Disease 6	Disease names related to acute heart failure in DPC (any of the six items^a^) and the hospital stay is three days or more.
Drug 1	Human atrial natriuretic peptide (carperitide) in the SS-MIX2, DPC, or the health insurance claims data.
Drug 2	Catecholamines or pimobendan in the SS-MIX2, DPC, or the health insurance claims data.
Drug 3	Potassium-sparing diuretics in the SS-MIX2, DPC, or the health insurance claims data.
Drug 4	Digitalis in the SS-MIX2, DPC, or the health insurance claims data.
Drug 5	Diuretics in the SS-MIX2, DPC, or the health insurance claims data.
Drug 6	Loop diuretics in the SS-MIX2, DPC, or the health insurance claims data.
Drug 7	Tolvaptan in the SS-MIX2, DPC, or the health insurance claims data.
Drug 8	Phosphodiesterase inhibitors in the SS-MIX2, DPC, or the health insurance claims data.
Blood test 1	The value of serum BNP that is greater than or equal to 100 pg/ml in SS-MIX2.
Blood test 2	The value of serum BNP that is greater than or equal to 400 pg/ml in SS-MIX2.
Blood test 3	The value of serum BNP that is between 100 pg/ml and 400 pg/ml in SS-MIX2.
Blood test 4	The value of serum NT-proBNP that is greater than or equal to 400 pg/ml in SS-MIX2.
Blood test 5	The value of serum NT-proBNP that is greater than or equal to 3,000 pg/ml in SS-MIX2.
Blood test 6	The value of serum NT-proBNP that is between 400 pg/ml and 3,000 pg/ml in SS-MIX2.
Medical Practice 1	Transthoracic echocardiography in DPC or the health insurance claims data.

^a^ The main diagnosis, the diagnosis causing admission, the most resource-consuming diagnoses, the second most resource-consuming diagnoses, the comorbidities present on admission, or the complications during admission.

^b^ The main diagnosis, the diagnosis causing admission, the most resource-consuming diagnoses.

#### Algorithm of APC

We defined the algorithm of APC as the combination of Disease 1, 3, or 5 and laboratory records for Blood test 1 or 4 within 30 days before or after the first date of the diagnosis dates for Disease 1–3. Values of serum BNP and NT-proBNP were used as criteria for acute heart failure referring to the guideline (20).

For all algorithms, the index date was defined as the first date of diagnosis (Disease 1–6) selected for each algorithm (admission date recorded in the DPC, start date recorded in the SS-MIX2, or start date recorded in health insurance claims data). The date was used to determine the temporal relationship between conditions for each algorithm.

#### Algorithm definitions

We developed 18 algorithms to identify true cases with acute heart failure (Table 2), based on various conditions shown in Table 1. Algorithm 1 came from our previous study using a machine-learning method (21). This algorithm encompassed patients with a disease name related to acute heart failure (Disease 1, 4, or 5), high BNP (Blood test 2) or moderate BNP (Blood test 3), and acute heart failure-related medications (Drug 1, 2, or 3, or Drug 4 and 5), with all components recorded within 28 days of the index date (i.e., 14 days before and after the index date). Algorithm 2 covered both confirmed and suspected cases of acute heart failure. For Algorithms 3–8, we extended Algorithms 1 and 2 by incorporating a wide range of perspectives discussed among clinical experts. In Algorithm 3, the time window to determine the temporal relationship between conditions was shortened to 14 days instead of 28 days. In Algorithm 4, we added the procedure of ultrasound cardiography to Algorithm 2. In Algorithm 5, a target disease name was limited to Disease 1, instead of Disease 1, 4, or 5 in Algorithm 2. In Algorithm 6, Disease 6 was used for a target disease name, instead of disease conditions in Algorithm 5. Similarly, a target disease name of Algorithm 2 was changed to Disease 2 or 4 in Algorithm 7 and to Disease 2 in Algorithm 8.

**Table 2:** Algorithms to identify patients with acute heart failure.

Algorithm number	Algorithm	Temporal relationship between the index date and conditions
1	1. [Disease 1, 4, or 5] and [Blood test 2].	All conditions were recorded within 28 days of the index date (14 days before and after the index date of the disease name)
	or	
	2. [Disease 1, 4, or 5], [Blood test 3], and [Drug 1, 2, or 3].	
	or	
	3. [Disease 1, 4, or 5], [Blood test 3], and [Drug 4 and 5].	
2	1. [Disease 1, 3, or 5] and [Blood test 2 or 5].	
	or	
	2. [Disease 1, 3, or 5], [Blood test 3 or 6], and [Drug 1, 2, or 3].	
	or	
	3. [Disease 1, 3, or 5], [Blood test 3 or 6], and [Drug 4 and 5].	
3	1. [Disease 1, 3, or 5] and [Blood test 2 or 5].	All conditions were recorded within 14 days of the index date (7 days before and after the index date of the disease name)
	Or	
	2. [Disease 1, 3, or 5], [Blood test 3 or 6], and [Drug 1, 2, or 3].	
	Or	
	3. [Disease 1, 3, or 5], [Blood test 3 or 6], and [Drug 4 and 5].	
4	1. [Disease 1, 3, or 5], [Blood test 2 or 5], and [Medical Practice 1].	All conditions were recorded within 28 days of the index date (14 days before and after the index date of the disease name)
	or	
	2. [Disease 1, 3, or 5], [Blood test 3 or 6], [Drug 1, 2, or 3], and [Medical Practice 1].	
	or	
	3. [Disease 1, 3, or 5], [Blood test 3 or 6], [Drug 4 and 5], and [Medical Practice 1].	
5	1. [Disease 1] and [Blood test 2 or 5].	
	or	
	2. [Disease 1], [Blood test 3 or 6], and [Drug 1, 2, or 3].	
	or	
	3. [Disease 1], [Blood test 3 or 6], and [Drug 4 and 5].	
6	1. [Disease 6] and [Blood test 2 or 5].	
	or	
	2. [Disease 6], [Blood test 3 or 6], and [Drug 1, 2, or 3].	
	or	
	3. [Disease 6], [Blood test 3 or 6], and [Drug 4 and 5].	
7	1. [Disease 2 or 4] and [Blood test 2 or 5].	
	or	
	2. [Disease 2 or 4], [Blood test 3 or 6], and [Drug 1, 2, or 3].	
	or	
	3. [Disease 2 or 4], [Blood test 3 or 6], and [Drug 4 and 5].	
8	1. [Disease 2] and [Blood test 2 or 5].	
	or	
	2. [Disease 2], [Blood test 3 or 6], and [Drug 1, 2, or 3].	
	or	
	3. [Disease 2], [Blood test 3 or 6], and [Drug 4 and 5].	
9	[Disease 1, 4, or 5], [Blood test 1], and [Drug 1, 6, 7 or 8].	All conditions were recorded within 60 days of the index date (30 days before and after the index date of the disease name)
10	[Disease 1, 3, or 5], [Blood test 1 or 4], and [Drug 1, 6, 7 or 8].	All conditions were recorded within 28 days of the index date (14 days before and after the index date of the disease name)
11	[Disease 1, 3, or 5], [Blood test 1 or 4], and [Drug 1, 6, 7 or 8].	All conditions were recorded within 14 days of the index date (7 days before and after the index date of the disease name)
12	[Disease 1, 3, or 5], [Blood test 1 or 4], [Drug 1, 6, 7 or 8], and [Medical Practice 1].	All conditions were recorded within 28 days of the index date (14 days before and after the index date of the disease name)
13	[Disease 1, 3, or 5], [Blood test 1 or 4], and [Drug 6].	
14	[Disease 1, 3, or 5], [Blood test 1 or 4], and [Drug 1].	
15	[Disease 1], [Blood test 1 or 4], and [Drug 1, 6, 7 or 8].	
16	[Disease 6], [Blood test 1 or 4], and [Drug 1, 6, 7 or 8].	
17	[Disease 2 or 4], [Blood test 1 or 4], and [Drug 1, 6, 7 or 8].	
18	[Disease 2], [Blood test 1 or 4], and [Drug 1, 6, 7 or 8].	

Furthermore, Algorithms 9 and 10 was constructed based on expert opinions or the clinical guideline (20) instead of the results from machine learning technique. Algorithm 9 employed Disease 1, 4, or 5 with Blood test 1 and Drug 1, 6, 7 or 8 within 60 days of the index date. Algorithm 10 included the disease name with a suspected diagnosis by replacing Disease 4 with 3, added Blood test 4 and used a 28-day time window. Algorithms 11–18 were developed by modifying Algorithms 9 and 10. In Algorithm 11, the time window was further shortened to 14 days of the index date based on the conditions of Algorithm 10. In Algorithm 12, we added Medical Practice 1 within 28 days of the index date to Algorithm 10. Algorithm 13 limited a target drug of Algorithm 10 to Drug 6 only. In Algorithm 14, Drug 1 was used instead of Drug 6 in Algorithm 13. In Algorithm 15, a target disease name was limited to Disease 1, instead of Disease 1, 3, or 5 in Algorithm 10. Similarly, in Algorithms 16, 17, and 18, a target disease name of Algorithm 10 was changed to Disease 6, Disease 2 or 4, and Disease 2, respectively.

### Criteria for true cases

**Figure 1:**
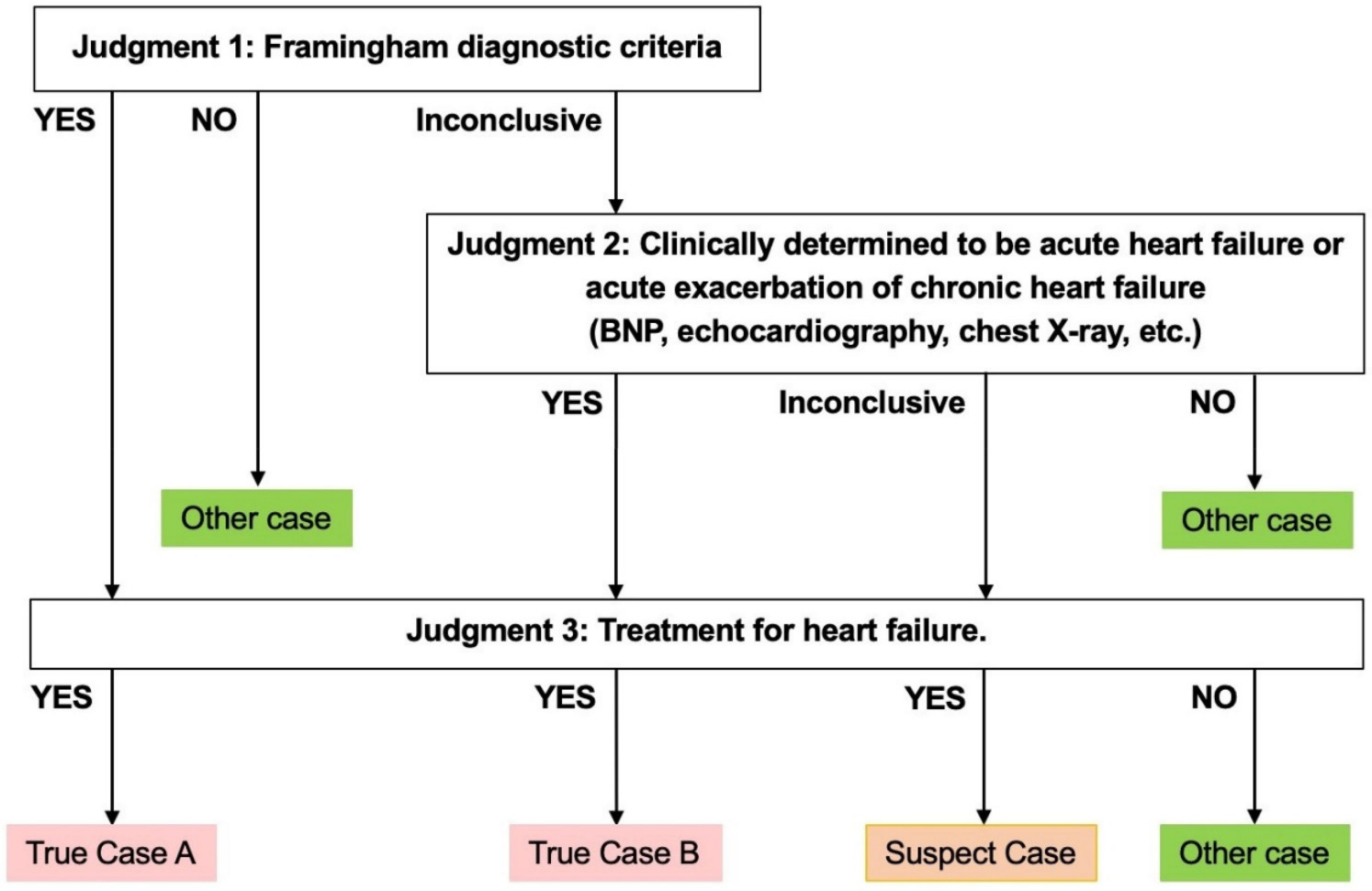
Criteria for determining a true case.

In this study, physicians conducted chart reviews on a random sample of patients who fulfilled the APC algorithm, without access to results. After the study population was identified, two physicians including at least one cardiologist in each facility independently adjudicated each case of the population. Disagreements between adjudicators were resolved through discussion between them. Each case was classified as True cases (True case A or True case B), Suspected cases, or Other cases, according to the three-step criteria as shown in Figure 1. The judgment criteria were created with the involvement of specialists, with reference to the “the Japanese Circulation Society 2017/the Japanese Heart Failure Society 2017 Guideline on diagnosis and treatment of acute and chronic heart failure” (20). Judgement 1 was based on the Framingham diagnostic criteria. Judgement 2 was comprehensive assessment by reviewers including specialists based on clinical findings related to acute heart failure, such as BNP or NT-proBNP concentrations, results from chest x-ray and ultrasound cardiography. Judgement 3 was set based on the treatment for acute heart failure including medications and surgery. A case with positive results according to Judgement 1 and 3 was labeled “True case A”. A case deemed inconclusive by Judgment 1 but positive by Judgement 2 and 3 was labeled “True case B”. A case with a positive result identified by Judgement 3 but inconclusive under Judgements 1 and 2 was labeled a “Suspected case”.

### Analysis

**“**Possible cases” in each algorithm were defined as the subjects to adjudication and who were extracted from the study population which was randomly sampled from the target population identified by the APC algorithm. In the primary analysis, both True cases A and B were categorized as “True cases”, and Suspected and Other cases were categorized as “Other cases”. In the sensitivity analysis, on the other hand, the categorization of “True cases” included not only True cases A and B but also Suspected cases, and only Other cases was categorized as “Other cases”.

To assess the validity of each algorithm, positive predictive value (PPV), sensitivity, negative predictive value (NPV), and specificity were calculated. Especially, PPVs were treated as major evaluation items. PPV, sensitivity, NPV, and specificity were calculated as described below formulas and the 95% confidence interval (95% CI) was also calculated for all validity measure using the Clopper–Pearson exact methods. To calculate validity measures, we defined true-positives as “True cases” identified for each algorithm and false-negatives were cases not identified by each algorithm but adjudicated as “True cases” within the study population. False positives were defined as “Other cases” for each algorithm. True negatives were calculated by subtracting true positives, false positives, and false negatives from the total number of patients at the three hospitals between March 8, 2021–March 31, 2021.

The Fleiss–Cohen weighted kappa coefficient was calculated as an indicator of the degree of inter-reviewer agreement of the study population (22, 23).

SAS version 9.4 (SAS Institute, Cary, NC, USA) was used for calculations.

PPV (%) = the number of true positives/(the number of true positives + the number of false positives) × 100
Sensitivity (%) = the number of true positives/(the number of true positives + the number of false negatives)* × 100
*(the number of true positives + the number of false negatives) = the number of True cases meeting the algorithm of APC, assuming 100% sensitivity
NPV (%) = the number of true negatives/(the number of false negatives + the number of true negatives) × 100
Specificity (%) = the number of true negatives/(the number of false positives + the number of true negatives) × 100

### Ethics

The study protocol was approved by the ethics committee of each site. The approval code for the coordinating institution was A2901, and the approval codes for Sites A, B, and C are 3,212, 2020-1-459, TGE00944-163. In the present study, the need to obtain informed consent from study subjects was waived according to the Ethical Guidelines for Medical and Health Research Involving Human Subjects in Japan because of the nature of this study as a noninvasive investigation with secondary use of data. Based on the recommendation of the ethics committees, subjects were provided with the ability to opt-out of study inclusion as announced on the notice boards or websites of each participating site.

## Results

**Figure 2:**
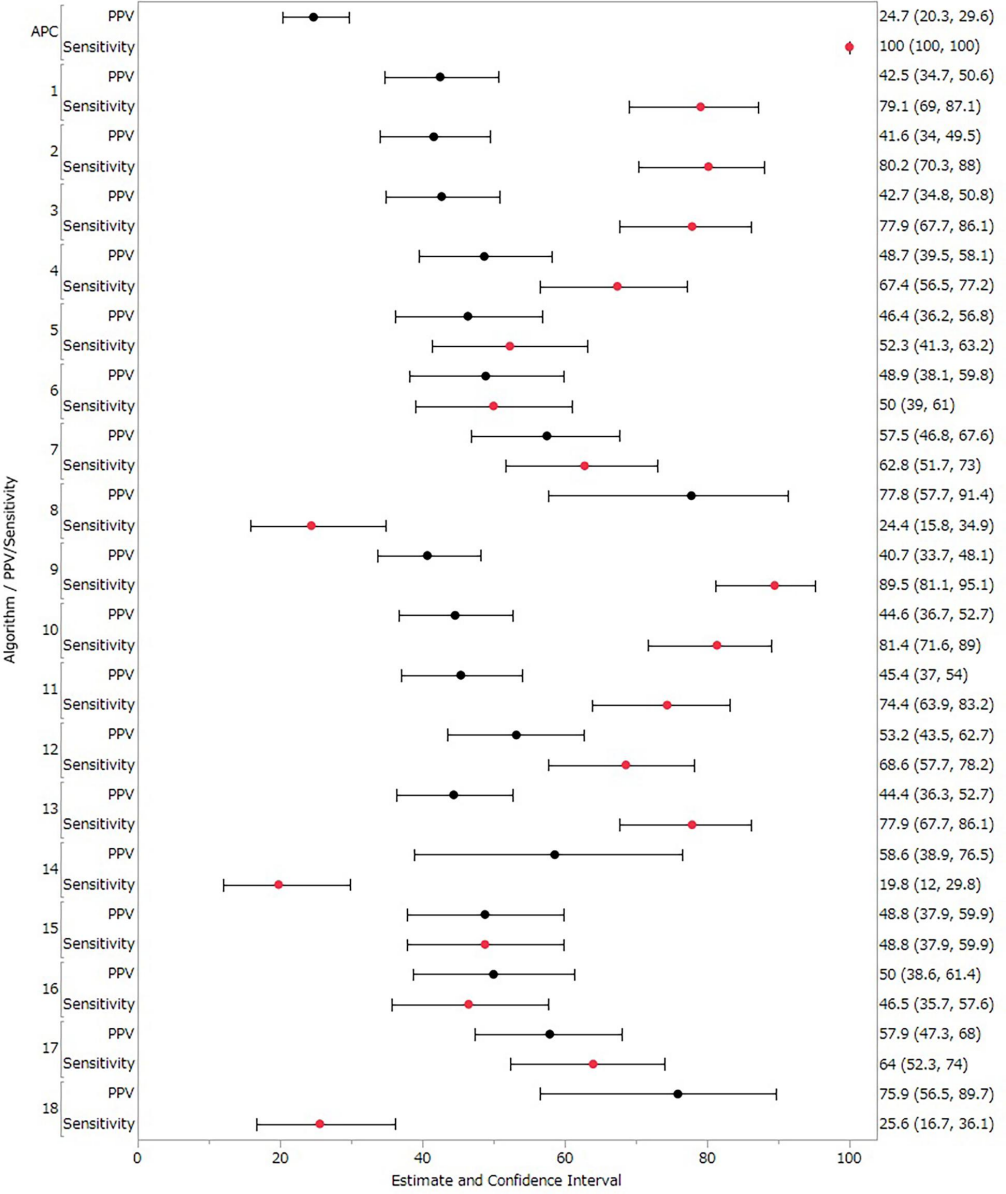
PPVs and sensitivity results in the primary analysis (treated suspected cases as other cases). Black circles represent positive predictive value (PPV), red circles represent sensitivity, and horizontal lines represent 95% confidence intervals (CI).

The degree of concordance among the evaluations of judges as assessed using the kappa coefficient was 0.94 (95% CI 0.90–0.98). This high level of agreement integrating results from the three medical institutions confirmed the reliability of the evaluations presented in Table 3. We assessed the validity of each algorithm based on PPV and sensitivity across the three medical institutions, assuming 100% sensitivity for the cases identified by the APC definition. For the primary analysis (treated Suspected cases as Other cases), PPV and sensitivity data for different algorithms are shown in Table 4 and Figure 2. Algorithm 1, developed from machine learning methods, yielded a PPV of 42.50% (95% CI 34.73–50.55) and a sensitivity of 79.07% (95% CI 68.95–87.10). Algorithm 2 evolved from Algorithm 1 by incorporating suspected disease names and the BNP/NT-proBNP test into the SS-MIX2 disease criteria, resulting in a sensitivity of 80.23% (95% CI 70.25–88.04). Algorithm 8, which narrowed the disease condition to three specific DPC entries, showed the highest PPV, at 77.78% (95% CI 57.74–91.38). Algorithm 9, which specified BNP testing and medication for acute heart failure without including Suspected cases, recorded the highest sensitivity, at 89.53% (95% CI 81.06–95.10). Algorithm 18, a variation of Algorithm 10 with restricted DPC information criteria, showed a PPV of 75.86% (95% CI 56.46–89.70).

**Table 3:** Final classification of all possible cases by chart reviews.

Number of cases judged by reviewers						
		Judgment by a reviewer				
		True case A	True case B	Suspect case	Other case	Total
Judgment by the other reviewer	True case A	57	4	1	3	65
	True case B	3	16	2	1	22
	Suspect case	0	1	6	1	8
	Other case	0	3	2	248	253
	Total	60	24	11	253	348

**Table 4:** PPVs and sensitivity results in the primary analysis (treated suspected cases as other cases).

Algorithm	Possible cases	True positives	False positives	False negatives	PPV (%)	95% CI	Sensitivity (%)	95% CI
APC	348	86	262	0	24.71	[20.27–29.59]	100.00	-
1	160	68	92	18	42.50	[34.73–50.55]	79.07	[68.95–87.10]
2	166	69	97	17	41.57	[33.98–49.46]	80.23	[70.25–88.04]
3	157	67	90	19	42.68	[34.83–50.81]	77.91	[67.67–86.14]
4	119	58	61	28	48.74	[39.47–58.07]	67.44	[56.48–77.16]
5	97	45	52	41	46.39	[36.20–56.81]	52.33	[41.27–63.21]
6	88	43	45	43	48.86	[38.05–59.75]	50.00	[39.02–60.98]
7	94	54	40	32	57.45	[46.82–67.59]	62.79	[51.70–72.98]
8	27	21	6	65	77.78	[57.74–91.38]	24.42	[15.80–34.87]
9	189	77	112	9	40.74	[33.67–48.11]	89.53	[81.06–95.10]
10	157	70	87	16	44.59	[36.66–52.72]	81.40	[71.55–88.98]
11	141	64	77	22	45.39	[36.99–53.98]	74.42	[63.87–83.22]
12	111	59	52	27	53.15	[43.45–62.69]	68.60	[57.70–78.19]
13	151	67	84	19	44.37	[36.30–52.67]	77.91	[67.67–86.14]
14	29	17	12	69	58.62	[38.94–76.48]	19.77	[11.96–29.75]
15	86	42	44	44	48.84	[37.90–59.86]	48.84	[37.90–59.86]
16	80	40	40	46	50.00	[38.60–61.40]	46.51	[35.68–57.59]
17	95	55	40	31	57.89	[47.33–67.96]	63.95	[52.88–74.03]
18	29	22	7	64	75.86	[56.46–89.70]	25.58	[16.78–36.13]

**Figure 3:**
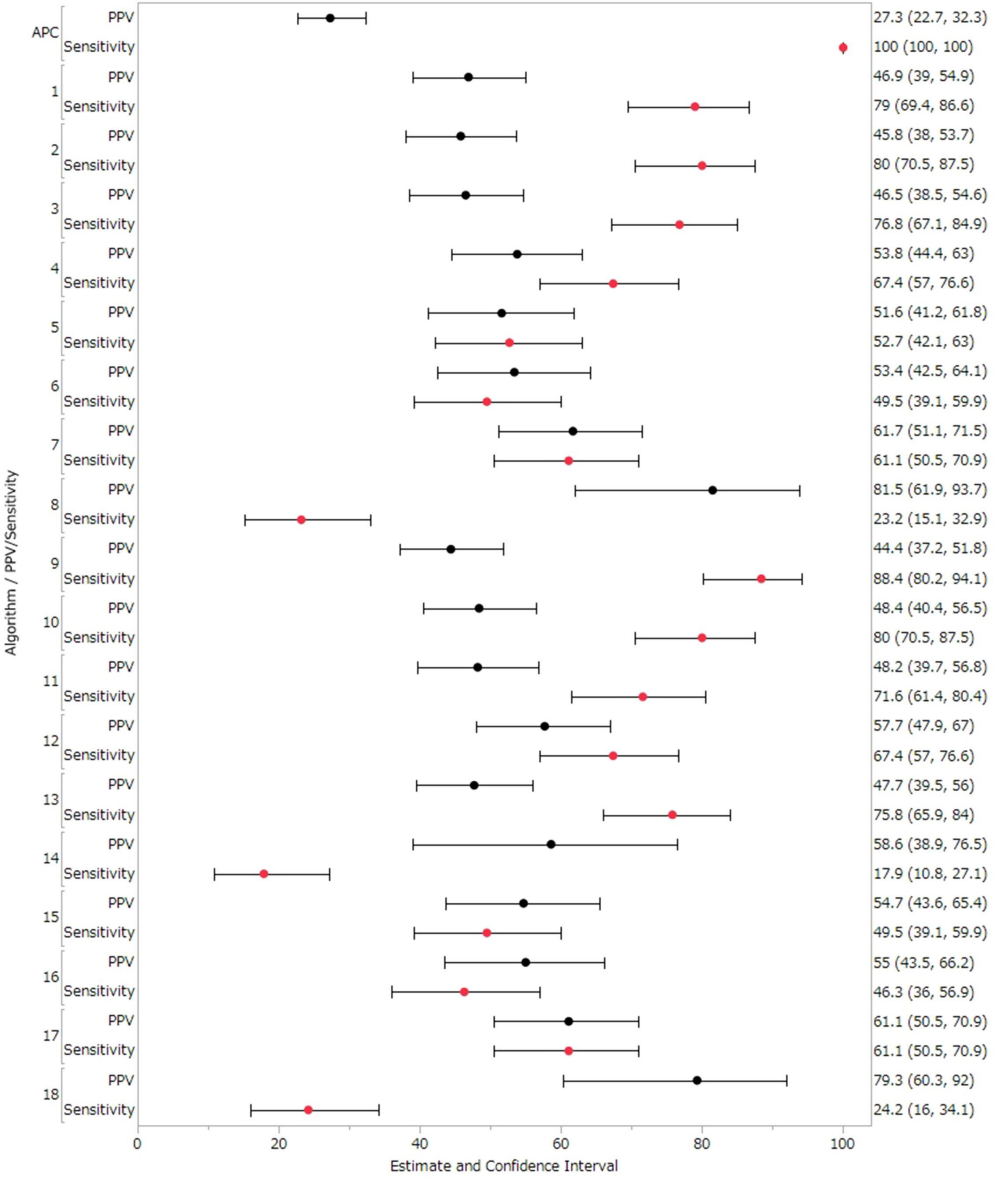
PPVs and sensitivity results in sensitivity analysis (treated suspected cases as true cases). Black circles represent positive predictive value (PPV), red circles represent sensitivity, and horizontal lines represent 95% confidence intervals (CI).

Table 5 and Figure 3 extends this analysis to include Suspected cases as True cases in the sensitivity analysis. In the sensitivity analysis, Algorithms 1–18 showed PPVs ranging from 44.44%–81.48% and sensitivities from 17.89%–88.42% (Table 5 and Figure 3). Algorithms 2 demonstrated a sensitivity of 80.00%, and Algorithms 8 achieved the highest PPV of 81.48%. Overall, results from the sensitivity analysis, which included Suspected cases as True cases, were consistent with the primary analysis. Detailed values for each facility are provided in Supplementary Tables S2, S3. Each algorithm showed high NPV and specificity and almost all values were nearly 100% (Supplementary Tables S4, S5).

**Table 5:** PPVs and sensitivity results in sensitivity analysis (treated suspected cases as true cases).

Algorithm	Possible cases	True positives	False positives	False negatives	PPV (%)	95% CI	Sensitivity (%)	95% CI
APC	348	95	253	0	27.30	[22.69–32.30]	100.00	–
1	160	75	85	20	46.88	[38.95–54.92]	78.95	[69.38–86.64]
2	166	76	90	19	45.78	[38.04–53.68]	80.00	[70.54–87.51]
3	157	73	84	22	46.50	[38.51–54.62]	76.84	[67.06–84.88]
4	119	64	55	31	53.78	[44.41–62.96]	67.37	[56.98–76.64]
5	97	50	47	45	51.55	[41.18–61.82]	52.63	[42.12–62.97]
6	88	47	41	48	53.41	[42.46–64.12]	49.47	[39.05–59.93]
7	94	58	36	37	61.70	[51.10–71.54]	61.05	[50.50–70.89]
8	27	22	5	73	81.48	[61.92–93.70]	23.16	[15.12–32.94]
9	189	84	105	11	44.44	[37.23–51.83]	88.42	[80.23–94.08]
10	157	76	81	19	48.41	[40.37–56.51]	80.00	[70.54–87.51]
11	141	68	73	27	48.23	[39.74–56.79]	71.58	[61.40–80.36]
12	111	64	47	31	57.66	[47.92–66.98]	67.37	[56.98–76.64]
13	151	72	79	23	47.68	[39.50–55.96]	75.79	[65.92–83.99]
14	29	17	12	78	58.62	[38.94–76.48]	17.89	[10.78–27.10]
15	86	47	39	48	54.65	[43.55–65.42]	49.47	[39.05–59.93]
16	80	44	36	51	55.00	[43.47–66.15]	46.32	[36.02–56.85]
17	95	58	37	37	61.05	[50.50–70.89]	61.05	[50.50–70.89]
18	29	23	6	72	79.31	[60.28–92.01]	24.21	[16.01–34.08]

Table 6 shows several common reasons for reviewers judging cases as Other cases, such as chronic heart failure without acute exacerbation, admission for examination of cardiac function, diagnosis of respiratory disease, and elevated BNP caused by renal dysfunction.

**Table 6:** Reasons for judgment of other cases (i.e., false positive cases).

Reasons for Judgment of Other cases in Hospital A	Number of cases
Chronic heart failure without acute exacerbation	15
Cardiac function is stable with treatment for arrhythmia	8
Follow-up examinations or postoperative assessments for cardiac conditions such as angina pectoris and myocardial infarction, in the absence of heart failure symptoms	9
Symptoms mainly due to respiratory disease (interstitial pneumonia, pulmonary embolism, etc.)	7
Evaluations of cardiac function for patients undergoing cancer treatment, in the absence of heart failure symptoms	10
Elevated BNP due to renal failure	10
Insufficient materials to determine (out-of-hospital cardiopulmonary arrest, death on arrival at hospital)	4
Other (rheumatoid arthritis, another disease such as arterial dissection, chest pain and cardiac enlargement)	11
Total	74
Reasons for Judgment of Other cases in Hospital B	Number of cases
Suspected disease name for BNP measurement	38
Chronic heart failure without acute exacerbation	50
Evaluations of cardiac function	3
Total	91
Reasons for Judgment of Other cases in Hospital C	Number of cases
Chronic heart failure without acute exacerbation	18
Cardiac function is stable with treatment for arrhythmia	2
Follow-up examinations or postoperative assessments for cardiac conditions such as angina pectoris and myocardial infarction, in the absence of heart failure symptoms	26
Symptoms mainly due to respiratory disease (interstitial pneumonia, pulmonary embolism, etc.)	8
Evaluations of cardiac function for patients undergoing cancer treatment, in the absence of heart failure symptoms	4
Other (rheumatoid arthritis, another disease such as arterial dissection, chest pain and cardiac enlargement)	30
Total	88

## Discussion

This study succeeded in delineating several algorithms critical for the precise identification of patients with acute heart failure. Selecting an optimal algorithm is essential, as PPV and sensitivity typically exhibit an inverse relationship. Notably, an algorithm that combines acute heart failure-related disease names with BNP measurement within 60 days of the index date achieved the highest sensitivity of 89.5% in our analysis. This contrasts with the findings of Tison et al. (24), who found that an algorithm using more than one heart failure code plus any heart failure medication offered only 67.2% sensitivity, decreasing further when elevated NT-proBNP was included. This variance may be attributed to differences in medication use and NT-proBNP threshold values.

In our pursuit of a higher PPV for identifying acute heart failure, we noted that Algorithms 8 and 18 yielded PPVs of 77.8% and 75.9%, respectively. This was consistent with the ranges reported in other studies (9, 25, 26). Some numerical differences between studies could stem from several factors. First, previous studies were confined to a single institution, whereas this study aggregated data from multiple sources. Second, updates in heart failure guidelines have influenced treatment protocols. Third, the inclusion of acute exacerbations of chronic heart failure broadened the scope of this study. In fact, Table 6 shows that false positive cases included many cases with stable chronic heart failure not in the exacerbation phase, suggesting that identifying exacerbation of chronic heart failure would require conditions in addition to the algorithm to detect acute heart failure. ICD-10 codes for congestive heart failure (I500) are consistent with acute heart failure or exacerbation of chronic heart failure and are difficult to distinguish by ICD-10 codes alone. Thus, we used cut-off values of BNP or NT-proBNP to detect cases of acute heart failure. However, those biomarker levels are already high in patients with chronic heart failure. To distinguish acute heart failure patients from chronic heart failure patients having high BNP levels even in a stable stage, use of relative BNP levels by comparing with BNP levels in the past may be appropriate. In addition, diuretics are commonly used to treat heart failure. Acute heart failure typically requires injectable forms, while chronic heart failure is almost always managed with oral forms. Therefore, checking the formulation of diuretics may be useful to further differentiate between acute and chronic heart failure. Notably, since symptoms of heart failure and renal failure (such as edema and dyspnea) often overlap and similar treatments (i.e., loop diuretics) are used for those, discerning the primary cause can be challenging in clinical practice. To exclude renal failure, further refinement of the algorithm is necessary, such as exclusion of patients with treatments not only common to both heart failure and renal failure (e.g., diuretics), but also with treatments specific to renal failure (e.g., serum potassium suppressants and activated charcoal, and dialysis). Excluding patients with persistently elevated BNP and prescribed treatments specific to renal failure may improve PPV.

“Other cases” were mainly assessments of cardiac function for patients such as those undergoing cancer treatment or surgery despite the absence of heart failure symptoms. Instances have been reported in which the diagnosis for health insurance claims was assigned merely to authorize tests for heart disease detection. This practice contributes to an inflated proportion of “Other cases” (false-positive) when attempting to identify genuine instances of the condition. In particular, several cases showed a postoperative decline in cardiac function and carperitide or catecholamine was used before diagnosis, resulting in false positive cases. Excluding cases of drug-induced heart failure that are unlikely to reflect genuine disease may lead to more well-balanced algorithms. Further improvement to algorithm depending on the research objectives may be warranted.

The study period for this study was 24 days in March, which was relatively short period. The same period was set to eliminate effects of time difference in extraction among different medical institutions. It has been known that the incidence of acute heart failure varies depending on the temperature (27). However, treatment approaches (i.e., records generated for medical interventions) generally remain consistent throughout the year in hospitals. Therefore, the results in this study could have certain generalizability.

Suspected cases accounted for about 10% of study population, which represented a no-negligible portion. However, as shown in Figures 2, 3, when Suspected cases were treated as True cases, the results across algorithms remained largely consistent, suggesting no major impact on algorithm selection.

Based on the discussions above, an appropriate algorithm should be selected depending on the research objective, taking into consideration the balance of PPV and sensitivity. For pharmacoepidemiological studies examining drug safety, it is preferable to use an algorithm that maintains reasonably high PPV while also ensuring sufficient sensitivity. From this veiwpoint, Algorithms 8 and 18 may not adequately detect drug safety signals because of lower sensitivity even in the highest PPV. In contrast, Algorithms 7 and 17 which demonstrated relatively high PPV along with a certain degree of sensitivity, could be appropriate for use in a study examining the risk of acute heart failure.

## Conclusion

This study clarified algorithms for identifying acute heart failure within a multi-institutional database. The validated algorithms for identifying acute heart failure were successfully established. Use of these outcomes should facilitate appropriate pharmacoepidemiological studies related to acute heart failure. Especially, Algorithm 7 and 17 may contribute to better drug safety assessments related to acute heart failure based on real-world data in Japan.

## Data Availability

The medical charts, DPC data, and healthcare insurance claims generated and analyzed during the present study are not publicly available because of ethical and legal restrictions in Japan (Act on the Protection of Personal Information). Requests to access the data can be directed to the corresponding author.
