# Supplementary material for: Establishment of reliable identification algorithms for acute heart failure or acute exacerbation of chronic heart failure using clinical data from a medical information database network
Source: Front Cardiovasc Med. 2025 Oct 15;12:1642323. doi: 10.3389/fcvm.2025.1642323 (PMC12568510; doi:10.3389/fcvm.2025.1642323)
Supplement: Supplementary file 1 [file Table1.pdf]

Supplementary Table 1. Code List.

Criteria Name	Table Name	Code Classification System	Code List	Code Description
Disease 1 and 6	07_DPC Disease Information	ICD-10 Code (Primary Diagnosis); ICD-10 Code (Diagnosis that led to hospitalization); ICD-10 Code (Diagnosis that required the most medical resources); ICD-10 Code (Diagnosis that required the second most medical resources); ICD-10 Code (Comorbidities at the time of hospitalization); ICD-10 Code (Diseases that developed after hospitalization)*	E059	Thyrotoxic heart failure, unspecified
Disease 1 and 6	07_DPC Disease Information	ICD-10 Code (Primary Diagnosis); ICD-10 Code (Diagnosis that led to hospitalization); ICD-10 Code (Diagnosis that required the most medical resources); ICD-10 Code (Diagnosis that required the second most medical resources); ICD-10 Code (Comorbidities at the time of hospitalization); ICD-10 Code (Diseases that developed after hospitalization)*	I099	Rheumatic heart failure, unspecified
Disease 1 and 6	07_DPC Disease Information	ICD-10 Code (Primary Diagnosis); ICD-10 Code (Diagnosis that led to hospitalization); ICD-10 Code (Diagnosis that required the most medical resources); ICD-10 Code (Diagnosis that required the second most medical resources); ICD-10 Code (Comorbidities at the time of hospitalization); ICD-10 Code (Diseases that developed after hospitalization)*	I110	Hypertensive heart disease with congestive heart failure
Disease 1 and 6	07_DPC Disease Information	ICD-10 Code (Primary Diagnosis); ICD-10 Code (Diagnosis that led to hospitalization); ICD-10 Code (Diagnosis that required the most medical resources); ICD-10 Code (Diagnosis that required the second most medical resources); ICD-10 Code (Comorbidities at the time of hospitalization); ICD-10 Code (Diseases that developed after hospitalization)*	I130	Hypertensive cardiorenal disease with congestive heart failure
Disease 1 and 6	07_DPC Disease Information	ICD-10 Code (Primary Diagnosis); ICD-10 Code (Diagnosis that led to hospitalization); ICD-10 Code (Diagnosis that required the most medical resources); ICD-10 Code (Diagnosis that required the second most medical resources); ICD-10 Code (Comorbidities at the time of hospitalization); ICD-10 Code (Diseases that developed after hospitalization)*	I132	Hypertensive cardiorenal disease with congestive heart failure and renal failure
Disease 1 and 6	07_DPC Disease Information	ICD-10 Code (Primary Diagnosis); ICD-10 Code (Diagnosis that led to hospitalization); ICD-10 Code (Diagnosis that required the most medical resources); ICD-10 Code (Diagnosis that required the second most medical resources); ICD-10 Code (Comorbidities at the time of hospitalization); ICD-10 Code (Diseases that developed after hospitalization)*	I500	Congestive heart failure
Disease 1 and 6	07_DPC Disease Information	ICD-10 Code (Primary Diagnosis); ICD-10 Code (Diagnosis that led to hospitalization); ICD-10 Code (Diagnosis that required the most medical resources); ICD-10 Code (Diagnosis that required the second most medical resources); ICD-10 Code (Comorbidities at the time of hospitalization); ICD-10 Code (Diseases that developed after hospitalization)*	I501	Left-sided heart failure
Disease 1 and 6	07_DPC Disease Information	ICD-10 Code (Primary Diagnosis); ICD-10 Code (Diagnosis that led to hospitalization); ICD-10 Code (Diagnosis that required the most medical resources); ICD-10 Code (Diagnosis that required the second most medical resources); ICD-10 Code (Comorbidities at the time of hospitalization); ICD-10 Code (Diseases that developed after hospitalization)*	I509	Heart failure (broad definition)
Disease 1 and 6	07_DPC Disease Information	ICD-10 Code (Primary Diagnosis); ICD-10 Code (Diagnosis that led to hospitalization); ICD-10 Code (Diagnosis that required the most medical resources); ICD-10 Code (Diagnosis that required the second most medical resources); ICD-10 Code (Comorbidities at the time of hospitalization); ICD-10 Code (Diseases that developed after hospitalization)*	O291	Heart failure due to anesthesia in pregnancy
Disease 1 and 6	07_DPC Disease Information	ICD-10 Code (Primary Diagnosis); ICD-10 Code (Diagnosis that led to hospitalization); ICD-10 Code (Diagnosis that required the most medical resources); ICD-10 Code (Diagnosis that required the second most medical resources); ICD-10 Code (Comorbidities at the time of hospitalization); ICD-10 Code (Diseases that developed after hospitalization)*	P290	Neonatal heart failure
Disease 1 and 6	07_DPC Disease Information	ICD-10 Code (Primary Diagnosis); ICD-10 Code (Diagnosis that led to hospitalization); ICD-10 Code (Diagnosis that required the most medical resources); ICD-10 Code (Diagnosis that required the second most medical resources); ICD-10 Code (Comorbidities at the time of hospitalization); ICD-10 Code (Diseases that developed after hospitalization)*	R570	Cardiogenic shock
Disease 2	07_DPC Disease Information	ICD-10 Code (Primary Diagnosis); ICD-10 Code (Diagnosis that led to hospitalization); ICD-10 Code (Diagnosis that required the most medical resources)	E059	Thyrotoxic heart failure, unspecified
Disease 2	07_DPC Disease Information	ICD-10 Code (Primary Diagnosis); ICD-10 Code (Diagnosis that led to hospitalization); ICD-10 Code (Diagnosis that required the most medical resources)	I099	Rheumatic heart failure, unspecified
Disease 2	07_DPC Disease Information	ICD-10 Code (Primary Diagnosis); ICD-10 Code (Diagnosis that led to hospitalization); ICD-10 Code (Diagnosis that required the most medical resources)	I110	Hypertensive heart disease with congestive heart failure
Disease 2	07_DPC Disease Information	ICD-10 Code (Primary Diagnosis); ICD-10 Code (Diagnosis that led to hospitalization); ICD-10 Code (Diagnosis that required the most medical resources)	I130	Hypertensive cardiorenal disease with congestive heart failure
Disease 2	07_DPC Disease Information	ICD-10 Code (Primary Diagnosis); ICD-10 Code (Diagnosis that led to hospitalization); ICD-10 Code (Diagnosis that required the most medical resources)	I132	Hypertensive cardiorenal disease with congestive heart failure and renal failure
Disease 2	07_DPC Disease Information	ICD-10 Code (Primary Diagnosis); ICD-10 Code (Diagnosis that led to hospitalization); ICD-10 Code (Diagnosis that required the most medical resources)	I500	Congestive heart failure
Disease 2	07_DPC Disease Information	ICD-10 Code (Primary Diagnosis); ICD-10 Code (Diagnosis that led to hospitalization); ICD-10 Code (Diagnosis that required the most medical resources)	I501	Left-sided heart failure
Disease 2	07_DPC Disease Information	ICD-10 Code (Primary Diagnosis); ICD-10 Code (Diagnosis that led to hospitalization); ICD-10 Code (Diagnosis that required the most medical resources)	I509	Heart failure (broad definition)
Disease 2	07_DPC Disease Information	ICD-10 Code (Primary Diagnosis); ICD-10 Code (Diagnosis that led to hospitalization); ICD-10 Code (Diagnosis that required the most medical resources)	O291	Heart failure due to anesthesia in pregnancy
Disease 2	07_DPC Disease Information	ICD-10 Code (Primary Diagnosis); ICD-10 Code (Diagnosis that led to hospitalization); ICD-10 Code (Diagnosis that required the most medical resources)	P290	Neonatal heart failure
Disease 2	07_DPC Disease Information	ICD-10 Code (Primary Diagnosis); ICD-10 Code (Diagnosis that led to hospitalization); ICD-10 Code (Diagnosis that required the most medical resources)	R570	Cardiogenic shock
Disease 3 and 4	01_Disease Information (Diagnosis)	Identification code for Disease Exchange (SS-MIX2)	B879	Hypertensive heart disease with congestive heart failure
Disease 3 and 4	01_Disease Information (Diagnosis)	Identification code for Disease Exchange (SS-MIX2)	BCEA	Myocardial failure
Disease 3 and 4	01_Disease Information (Diagnosis)	Identification code for Disease Exchange (SS-MIX2)	BF6C	Cardiogenic pulmonary edema
Disease 3 and 4	01_Disease Information (Diagnosis)	Identification code for Disease Exchange (SS-MIX2)	CJ4E	Thyrotoxic heart failure
Disease 3 and 4	01_Disease Information (Diagnosis)	Identification code for Disease Exchange (SS-MIX2)	CLJA	Cardiogenic shock
Disease 3 and 4	01_Disease Information (Diagnosis)	Identification code for Disease Exchange (SS-MIX2)	D3MU	Cardiac asthma
Disease 3 and 4	01_Disease Information (Diagnosis)	Identification code for Disease Exchange (SS-MIX2)	DGBT	Neonatal heart failure
Disease 3 and 4	01_Disease Information (Diagnosis)	Identification code for Disease Exchange (SS-MIX2)	FP5V	Acute heart failure
Disease 3 and 4	01_Disease Information (Diagnosis)	Identification code for Disease Exchange (SS-MIX2)	G7RM	Cardiac edema
Disease 3 and 4	01_Disease Information (Diagnosis)	Identification code for Disease Exchange (SS-MIX2)	HCSK	Rheumatic heart failure
Disease 3 and 4	01_Disease Information (Diagnosis)	Identification code for Disease Exchange (SS-MIX2)	HD64	Heart failure due to anesthesia in pregnancy
Disease 3 and 4	01_Disease Information (Diagnosis)	Identification code for Disease Exchange (SS-MIX2)	J9J2	Bilateral heart failure
Disease 3 and 4	01_Disease Information (Diagnosis)	Identification code for Disease Exchange (SS-MIX2)	K41T	Chronic congestive heart failure
Disease 3 and 4	01_Disease Information (Diagnosis)	Identification code for Disease Exchange (SS-MIX2)	NJES	Chronic heart failure
Disease 3 and 4	01_Disease Information (Diagnosis)	Identification code for Disease Exchange (SS-MIX2)	QB3C	Cardiac dyspnea
Disease 3 and 4	01_Disease Information (Diagnosis)	Identification code for Disease Exchange (SS-MIX2)	R24B	Congestive heart failure
Disease 3 and 4	01_Disease Information (Diagnosis)	Identification code for Disease Exchange (SS-MIX2)	RFHT	Hypertensive heart disease with congestive heart failure
Disease 3 and 4	01_Disease Information (Diagnosis)	Identification code for Disease Exchange (SS-MIX2)	S01N	Reight-sided heart failure
Disease 3 and 4	01_Disease Information (Diagnosis)	Identification code for Disease Exchange (SS-MIX2)	S0K8	Left-sided heart failure
Disease 3 and 4	01_Disease Information (Diagnosis)	Identification code for Disease Exchange (SS-MIX2)	S0K8	Left ventricular heart failure
Disease 3 and 4	01_Disease Information (Diagnosis)	Identification code for Disease Exchange (SS-MIX2)	SU8P	Right ventricular heart failure
Disease 3 and 4	01_Disease Information (Diagnosis)	Identification code for Disease Exchange (SS-MIX2)	U9GK	Heart failure (broad definition)
Disease 5	13_Receipt Disease Information	Electronic medical claims processing code	2429015	Thyrotoxic heart failure
Disease 5	13_Receipt Disease Information	Electronic medical claims processing code	3089003	Cardiogenic shock
Disease 5	13_Receipt Disease Information	Electronic medical claims processing code	4280002	Cardiac edema
Disease 5	13_Receipt Disease Information	Electronic medical claims processing code	4280005	Chronic congestive heart failure
Disease 5	13_Receipt Disease Information	Electronic medical claims processing code	4280011	Reight-sided heart failure
Disease 5	13_Receipt Disease Information	Electronic medical claims processing code	4281005	Cardiac asthma
Disease 5	13_Receipt Disease Information	Electronic medical claims processing code	4281009	Left-sided heart failure
Disease 5	13_Receipt Disease Information	Electronic medical claims processing code	4281010	Cardiac dyspnea
Disease 5	13_Receipt Disease Information	Electronic medical claims processing code	4289005	Acute heart failure
Disease 5	13_Receipt Disease Information	Electronic medical claims processing code	4289015	Heart failure (broad definition)
Disease 5	13_Receipt Disease Information	Electronic medical claims processing code	4289018	Chronic heart failure
Disease 5	13_Receipt Disease Information	Electronic medical claims processing code	5140016	Cardiogenic pulmonary edema
Disease 5	13_Receipt Disease Information	Electronic medical claims processing code	8830796	Congestive heart failure
Disease 5	13_Receipt Disease Information	Electronic medical claims processing code	8833422	Hypertensive heart disease with congestive heart failure
Disease 5	13_Receipt Disease Information	Electronic medical claims processing code	8833426	Hypertensive heart failure
Disease 5	13_Receipt Disease Information	Electronic medical claims processing code	8834012	Left-sided heart failure
Disease 5	13_Receipt Disease Information	Electronic medical claims processing code	8834931	Myocardial failure
Disease 5	13_Receipt Disease Information	Electronic medical claims processing code	8835038	Neonatal heart failure
Disease 5	13_Receipt Disease Information	Electronic medical claims processing code	8838629	Heart failure due to anesthesia in pregnancy
Disease 5	13_Receipt Disease Information	Electronic medical claims processing code	8840943	Rheumatic heart failure
Disease 5	13_Receipt Disease Information	Electronic medical claims processing code	8841016	Bilateral heart failure
Disease 5	13_Receipt Disease Information	Electronic medical claims processing code	8842461	Reight-sided heart failure
Drug 1	22_Prescription/Injection Order or Administration	YJ Code	2179400D1022	HANP For injection
Drug 1	22_Prescription/Injection Order or Administration	YJ Code	2179400D1MID	Carperitide
Drug 1	23_DPC or Receipt Pharmaceutical Information	Electronic medical claims processing code	640407007	HANP For injection

Drug 2	22_Prescription/Injection Order or Administration	YJ Code	2119400A1028	Proternol-L injection 0.2mg
Drug 2	22_Prescription/Injection Order or Administration	YJ Code	2119400A1036	Proternol-L injection 0.2mg
Drug 2	22_Prescription/Injection Order or Administration	YJ Code	2119400A1MID	l-Isoprenaline hydrochloride
Drug 2	22_Prescription/Injection Order or Administration	YJ Code	2119400A2024	Proternol-L injection 1mg
Drug 2	22_Prescription/Injection Order or Administration	YJ Code	2119400A2032	Proternol-L injection 1mg
Drug 2	22_Prescription/Injection Order or Administration	YJ Code	2119400A2MID	Generic name code
Drug 2	22_Prescription/Injection Order or Administration	YJ Code	2119402A1027	Actopamin injection
Drug 2	22_Prescription/Injection Order or Administration	YJ Code	2119402A1035	Inovan injection
Drug 2	22_Prescription/Injection Order or Administration	YJ Code	2119402A1043	Evetant injection
Drug 2	22_Prescription/Injection Order or Administration	YJ Code	2119402A1060	Dopamine hydrochloride injection [KOBAYASHI]
Drug 2	22_Prescription/Injection Order or Administration	YJ Code	2119402A1078	Dopamine hydrochloride injection [SHIONOGI]100mg
Drug 2	22_Prescription/Injection Order or Administration	YJ Code	2119402A1086	Dopamine hydrochloride injection [DJ]
Drug 2	22_Prescription/Injection Order or Administration	YJ Code	2119402A1094	Kakodin injection
Drug 2	22_Prescription/Injection Order or Administration	YJ Code	2119402A1116	Gabans injection
Drug 2	22_Prescription/Injection Order or Administration	YJ Code	2119402A1124	Catherine injection
Drug 2	22_Prescription/Injection Order or Administration	YJ Code	2119402A1132	Critpan injection
Drug 2	22_Prescription/Injection Order or Administration	YJ Code	2119402A1167	Tsurudopami injection
Drug 2	22_Prescription/Injection Order or Administration	YJ Code	2119402A1175	Tronjin injection 100
Drug 2	22_Prescription/Injection Order or Administration	YJ Code	2119402A1205	Doparalmin injection
Drug 2	22_Prescription/Injection Order or Administration	YJ Code	2119402A1213	Dominin injection
Drug 2	22_Prescription/Injection Order or Administration	YJ Code	2119402A1221	Dolpamil injection
Drug 2	22_Prescription/Injection Order or Administration	YJ Code	2119402A1248	Martburn injection
Drug 2	22_Prescription/Injection Order or Administration	YJ Code	2119402A1264	Yaelista injection
Drug 2	22_Prescription/Injection Order or Administration	YJ Code	2119402A1272	Taiadopa injection
Drug 2	22_Prescription/Injection Order or Administration	YJ Code	2119402A1280	Dopamine injection 2% [KN]
Drug 2	22_Prescription/Injection Order or Administration	YJ Code	2119402A1299	Inovan injection 100mg
Drug 2	22_Prescription/Injection Order or Administration	YJ Code	2119402A1302	Doparalmin injection 100mg
Drug 2	22_Prescription/Injection Order or Administration	YJ Code	2119402A1310	Kakodin injection 100mg
Drug 2	22_Prescription/Injection Order or Administration	YJ Code	2119402A1329	Gabans injection 100mg
Drug 2	22_Prescription/Injection Order or Administration	YJ Code	2119402A1337	Dominin injection 100mg
Drug 2	22_Prescription/Injection Order or Administration	YJ Code	2119402A1345	Martburnin jection 100mg
Drug 2	22_Prescription/Injection Order or Administration	YJ Code	2119402A1353	Dopamine hydrochloride injection 100mg [IROM]
Drug 2	22_Prescription/Injection Order or Administration	YJ Code	2119402A1361	Dopamine hydrochloride injection 100mg [TAIYO]
Drug 2	22_Prescription/Injection Order or Administration	YJ Code	2119402A1370	Yaelista injection 100mg
Drug 2	22_Prescription/Injection Order or Administration	YJ Code	2119402A1388	Dopamine hydrochloride injection 100mg [KN]
Drug 2	22_Prescription/Injection Order or Administration	YJ Code	2119402A1396	Evetant injection 100mg
Drug 2	22_Prescription/Injection Order or Administration	YJ Code	2119402A1400	Tsurudopami injection 100mg
Drug 2	22_Prescription/Injection Order or Administration	YJ Code	2119402A1418	Critpan injection 100mg
Drug 2	22_Prescription/Injection Order or Administration	YJ Code	2119402A1426	Dopamine hydrochloride injection 100mg [NP]
Drug 2	22_Prescription/Injection Order or Administration	YJ Code	2119402A1434	Dopamine hydrochloride injection 100mg [PFIZER]
Drug 2	22_Prescription/Injection Order or Administration	YJ Code	2119402A1442	Dopamine hydrochloride injection 100mg [ISEI]
Drug 2	22_Prescription/Injection Order or Administration	YJ Code	2119402A1450	Dopamine hydrochloride injection 100mg [TSURUHARA]
Drug 2	22_Prescription/Injection Order or Administration	YJ Code	2119402A1469	Dopamine hydrochloride injection 100mg [KCC]
Drug 2	22_Prescription/Injection Order or Administration	YJ Code	2119402A1477	Dopamine hydrochloride injection 100mg [NIG]
Drug 2	22_Prescription/Injection Order or Administration	YJ Code	2119402A1MID	Generic name code
Drug 2	22_Prescription/Injection Order or Administration	YJ Code	2119402A2023	Dominin injection
Drug 2	22_Prescription/Injection Order or Administration	YJ Code	2119402A2031	Dominin injection 40mg
Drug 2	22_Prescription/Injection Order or Administration	YJ Code	2119402A2MID	Generic name code
Drug 2	22_Prescription/Injection Order or Administration	YJ Code	2119402A3020	Inovan injection
Drug 2	22_Prescription/Injection Order or Administration	YJ Code	2119402A3038	Tsurudopami injection
Drug 2	22_Prescription/Injection Order or Administration	YJ Code	2119402A3054	Inovan injection 50mg
Drug 2	22_Prescription/Injection Order or Administration	YJ Code	2119402A3062	Dopamine hydrochloride injection 50mg [KN]
Drug 2	22_Prescription/Injection Order or Administration	YJ Code	2119402A3070	Critpan injection 50mg
Drug 2	22_Prescription/Injection Order or Administration	YJ Code	2119402A3089	Evetant injection 50mg
Drug 2	22_Prescription/Injection Order or Administration	YJ Code	2119402A3097	Dopamine hydrochloride injection 50mg [IROM]
Drug 2	22_Prescription/Injection Order or Administration	YJ Code	2119402A3100	Dopamine hydrochloride injection 50mg [TAIYO]
Drug 2	22_Prescription/Injection Order or Administration	YJ Code	2119402A3119	Martburn injection 50mg
Drug 2	22_Prescription/Injection Order or Administration	YJ Code	2119402A3127	Yaelista injection 50mg
Drug 2	22_Prescription/Injection Order or Administration	YJ Code	2119402A3135	Tsurudopami injection 50mg
Drug 2	22_Prescription/Injection Order or Administration	YJ Code	2119402A3143	Doparalmin injection 50mg
Drug 2	22_Prescription/Injection Order or Administration	YJ Code	2119402A3151	Kakodin injection 50mg
Drug 2	22_Prescription/Injection Order or Administration	YJ Code	2119402A3160	Dopamine hydrochloride injection 50mg [NP]
Drug 2	22_Prescription/Injection Order or Administration	YJ Code	2119402A3178	Dopamine hydrochloride injection 50mg [NIG]
Drug 2	22_Prescription/Injection Order or Administration	YJ Code	2119402A3MID	Generic name code
Drug 2	22_Prescription/Injection Order or Administration	YJ Code	2119402A4026	Inovan injection
Drug 2	22_Prescription/Injection Order or Administration	YJ Code	2119402A4050	Tsurudopami injection
Drug 2	22_Prescription/Injection Order or Administration	YJ Code	2119402A4085	Dominin injection
Drug 2	22_Prescription/Injection Order or Administration	YJ Code	2119402A4093	Dominin injection 200mg
Drug 2	22_Prescription/Injection Order or Administration	YJ Code	2119402A4107	Inovan injection 200mg
Drug 2	22_Prescription/Injection Order or Administration	YJ Code	2119402A4115	Dopamine hydrochloride injection 200mg [KN]
Drug 2	22_Prescription/Injection Order or Administration	YJ Code	2119402A4123	Tsurudopami injection 200mg
Drug 2	22_Prescription/Injection Order or Administration	YJ Code	2119402A4131	Critpan injection 200mg
Drug 2	22_Prescription/Injection Order or Administration	YJ Code	2119402A4140	Doparalmin injection 200mg
Drug 2	22_Prescription/Injection Order or Administration	YJ Code	2119402A4158	Evetant injection 200mg
Drug 2	22_Prescription/Injection Order or Administration	YJ Code	2119402A4166	Dopamine hydrochloride injection 200mg [IROM]
Drug 2	22_Prescription/Injection Order or Administration	YJ Code	2119402A4174	Kakodin injection 200mg
Drug 2	22_Prescription/Injection Order or Administration	YJ Code	2119402A4182	Dopamine hydrochloride injection 200mg [TAIYO]
Drug 2	22_Prescription/Injection Order or Administration	YJ Code	2119402A4190	Martburn injection 200mg
Drug 2	22_Prescription/Injection Order or Administration	YJ Code	2119402A4204	Yaelista injection 200mg
Drug 2	22_Prescription/Injection Order or Administration	YJ Code	2119402A4212	Dopamine hydrochloride injection 200mg [NP]
Drug 2	22_Prescription/Injection Order or Administration	YJ Code	2119402A4220	Dopamine hydrochloride injection 200mg [NIG]
Drug 2	22_Prescription/Injection Order or Administration	YJ Code	2119402A4MID	Generic name code
Drug 2	22_Prescription/Injection Order or Administration	YJ Code	2119402G6021	Kakodin-D injection 200
Drug 2	22_Prescription/Injection Order or Administration	YJ Code	2119402G6064	Dopamine hydrochloride injection kit 200
Drug 2	22_Prescription/Injection Order or Administration	YJ Code	2119402G6080	Dopamine 200 [TOWA]
Drug 2	22_Prescription/Injection Order or Administration	YJ Code	2119402G6099	Kakodin-D injection 0.1%
Drug 2	22_Prescription/Injection Order or Administration	YJ Code	2119402G6MID	Generic name code
Drug 2	22_Prescription/Injection Order or Administration	YJ Code	2119402G7028	Kakodin-D injection 600
Drug 2	22_Prescription/Injection Order or Administration	YJ Code	2119402G7060	Dopamine hydrochloride injection kit 600
Drug 2	22_Prescription/Injection Order or Administration	YJ Code	2119402G7087	Dopamine injection 600 [TOWA]
Drug 2	22_Prescription/Injection Order or Administration	YJ Code	2119402G7095	Kakodin-D injection 0.3%
Drug 2	22_Prescription/Injection Order or Administration	YJ Code	2119402G7MID	Generic name code
Drug 2	22_Prescription/Injection Order or Administration	YJ Code	2119402P1020	Predopa injection 200
Drug 2	22_Prescription/Injection Order or Administration	YJ Code	2119402P1038	Kakodin-D injection 200
Drug 2	22_Prescription/Injection Order or Administration	YJ Code	2119402P1046	Catabon-Low
Drug 2	22_Prescription/Injection Order or Administration	YJ Code	2119402P1062	Dopamine hydrochloride injection kit 200
Drug 2	22_Prescription/Injection Order or Administration	YJ Code	2119402P1070	Catabon-Low injection 200mg
Drug 2	22_Prescription/Injection Order or Administration	YJ Code	2119402P1089	Kakodin-D injection 0.1%
Drug 2	22_Prescription/Injection Order or Administration	YJ Code	2119402P1097	Dopamine hydrochloride injection 200mg kit [PFIZER]
Drug 2	22_Prescription/Injection Order or Administration	YJ Code	2119402P1100	Dopamine hydrochloride injection 200mg bag [TAKEDA TEVA]
Drug 2	22_Prescription/Injection Order or Administration	YJ Code	2119402P1119	Dopamine hydrochloride injection 200mg bag [NICHIYAKU]
Drug 2	22_Prescription/Injection Order or Administration	YJ Code	2119402P1127	Dopamine hydrochloride injection 200mg kit [KCC]
Drug 2	22_Prescription/Injection Order or Administration	YJ Code	2119402P1135	Dopamine hydrochloride injection 200mg bag [NIG]
Drug 2	22_Prescription/Injection Order or Administration	YJ Code	2119402P1MID	Generic name code
Drug 2	22_Prescription/Injection Order or Administration	YJ Code	2119402P2026	Predopa injection 600
Drug 2	22_Prescription/Injection Order or Administration	YJ Code	2119402P2034	Kakodin-D injection 600
Drug 2	22_Prescription/Injection Order or Administration	YJ Code	2119402P2042	Catabon-Hi
Drug 2	22_Prescription/Injection Order or Administration	YJ Code	2119402P2069	Dopamine hydrochloride injection kit 600
Drug 2	22_Prescription/Injection Order or Administration	YJ Code	2119402P2077	Catabon-Hi injection 600mg
Drug 2	22_Prescription/Injection Order or Administration	YJ Code	2119402P2085	Kakodin-D injection 0.3%
Drug 2	22_Prescription/Injection Order or Administration	YJ Code	2119402P2093	Dopamine liquid 600 [TOWA]
Drug 2	22_Prescription/Injection Order or Administration	YJ Code	2119402P2107	Dopamine hydrochloride injection 600mg kit [PFIZER]
Drug 2	22_Prescription/Injection Order or Administration	YJ Code	2119402P2115	Dopamine hydrochloride injection 600mg bag [TAKEDA TEVA]
Drug 2	22_Prescription/Injection Order or Administration	YJ Code	2119402P2123	Dopamine hydrochloride injection 600mg bag [NICHIYAKU]
Drug 2	22_Prescription/Injection Order or Administration	YJ Code	2119402P2131	Dopamine hydrochloride injection 600mg kit [KCC]
Drug 2	22_Prescription/Injection Order or Administration	YJ Code	2119402P2140	Dopamine hydrochloride injection 600mg bag [NIG]
Drug 2	22_Prescription/Injection Order or Administration	YJ Code	2119402P2MID	Generic name code
Drug 2	22_Prescription/Injection Order or Administration	YJ Code	2119402P3022	Inovan 0.1% injection syringe
Drug 2	22_Prescription/Injection Order or Administration	YJ Code	2119402P3030	Inovan injection 0.1% syringe
Drug 2	22_Prescription/Injection Order or Administration	YJ Code	2119402P3MID	Generic name code
Drug 2	22_Prescription/Injection Order or Administration	YJ Code	2119402P4029	Inovan 0.3% injection syringe
Drug 2	22_Prescription/Injection Order or Administration	YJ Code	2119402P4037	Inovan injection 0.3% syringe
Drug 2	22_Prescription/Injection Order or Administration	YJ Code	2119402P4MID	Generic name code
Drug 2	22_Prescription/Injection Order or Administration	YJ Code	2119402P5025	Inovan injection 0.6% syringe
Drug 2	22_Prescription/Injection Order or Administration	YJ Code	2119402P5MID	Generic name code
Drug 2	22_Prescription/Injection Order or Administration	YJ Code	2119403A1021	Araminon injection
Drug 2	22_Prescription/Injection Order or Administration	YJ Code	2119403A1MID	Generic name code
Drug 2	22_Prescription/Injection Order or Administration	YJ Code	2119404A1026	Dobutrex injection 100mg
Drug 2	22_Prescription/Injection Order or Administration	YJ Code	2119404A1034	Retamex
Drug 2	22_Prescription/Injection Order or Administration	YJ Code	2119404A1042	Generic dobutamine hydrochloride injection (discontinued drug)
Drug 2	22_Prescription/Injection Order or Administration	YJ Code	2119404A1050	Dobux injection
Drug 2	22_Prescription/Injection Order or Administration	YJ Code	2119404A1069	Generic dobutamine hydrochloride injection 100mg (discontinued drug)
Drug 2	22_Prescription/Injection Order or Administration	YJ Code	2119404A1077	Bubucin injection
Drug 2	22_Prescription/Injection Order or Administration	YJ Code	2119404A1085	Doputamin-H injection
Drug 2	22_Prescription/Injection Order or Administration	YJ Code	2119404A1093	Dopmin injection
Drug 2	22_Prescription/Injection Order or Administration	YJ Code	2119404A1107	Generic dobutamine hydrochloride injection (discontinued drug)
Drug 2	22_Prescription/Injection Order or Administration	YJ Code	2119404A1115	Dobutamine hydrochloride injection 100mg
Drug 2	22_Prescription/Injection Order or Administration	YJ Code	2119404A1123	Dopmin injection 100mg
Drug 2	22_Prescription/Injection Order or Administration	YJ Code	2119404A1131	Bubucin injection 100mg
Drug 2	22_Prescription/Injection Order or Administration	YJ Code	2119404A1140	Dobutamine injection 100mg [IROM]
Drug 2	22_Prescription/Injection Order or Administration	YJ Code	2119404A1158	Doputamin injection 100mg
Drug 2	22_Prescription/Injection Order or Administration	YJ Code	2119404A1166	Dobux injection 100mg
Drug 2	22_Prescription/Injection Order or Administration	YJ Code	2119404A1174	Retamex injection 100mg
Drug 2	22_Prescription/Injection Order or Administration	YJ Code	2119404A1182	Dobutamine hydrochloride injection 100mg [SAWAI]
Drug 2	22_Prescription/Injection Order or Administration	YJ Code	2119404A1190	Dobutamine injection 100mg [AFP]
Drug 2	22_Prescription/Injection Order or Administration	YJ Code	2119404A1204	Dobutamine hydrochloride injection 100mg [KN]
Drug 2	22_Prescription/Injection Order or Administration	YJ Code	2119404A1212	Dobutamine injection 100mg [F]
Drug 2	22_Prescription/Injection Order or Administration	YJ Code	2119404A1220	Dobutamine injection 100mg [PFIZER]
Drug 2	22_Prescription/Injection Order or Administration	YJ Code	2119404A1MID	Generic name code

Drug 2	22_Prescription/Injection Order or Administration	YJ Code	2119404G1029	Dobutrex K injection 200mg
Drug 2	22_Prescription/Injection Order or Administration	YJ Code	2119404G1MID	Generic name code
Drug 2	22_Prescription/Injection Order or Administration	YJ Code	2119404G2025	Dobutrex K injection 600mg
Drug 2	22_Prescription/Injection Order or Administration	YJ Code	2119404G2033	Doputamin K injection 600mg
Drug 2	22_Prescription/Injection Order or Administration	YJ Code	2119404G2MID	Generic name code
Drug 2	22_Prescription/Injection Order or Administration	YJ Code	2119404G3021	Dopmin K injection 200
Drug 2	22_Prescription/Injection Order or Administration	YJ Code	2119404G3030	Dobutrex K injection 200mg
Drug 2	22_Prescription/Injection Order or Administration	YJ Code	2119404G3048	Dobutrex injection 200mg bag
Drug 2	22_Prescription/Injection Order or Administration	YJ Code	2119404G3056	Dobutrex kit injection 200mg
Drug 2	22_Prescription/Injection Order or Administration	YJ Code	2119404G3064	Dobutamine injection 200mg kit [PFIZER]
Drug 2	22_Prescription/Injection Order or Administration	YJ Code	2119404G3MID	Generic name code
Drug 2	22_Prescription/Injection Order or Administration	YJ Code	2119404G4028	Dopmin K injection 600
Drug 2	22_Prescription/Injection Order or Administration	YJ Code	2119404G4036	Dobutrex K injection 600mg
Drug 2	22_Prescription/Injection Order or Administration	YJ Code	2119404G4044	Dobutrex injection 600mg bag
Drug 2	22_Prescription/Injection Order or Administration	YJ Code	2119404G4052	Dobutrex kit injection 600mg
Drug 2	22_Prescription/Injection Order or Administration	YJ Code	2119404G4060	Dobutamine injection 600mg kit [PFIZER]
Drug 2	22_Prescription/Injection Order or Administration	YJ Code	2119404G4MID	Generic name code
Drug 2	22_Prescription/Injection Order or Administration	YJ Code	2119404G5024	Dobupum 0.1% injection syringe
Drug 2	22_Prescription/Injection Order or Administration	YJ Code	2119404G5032	Dobupum injection 0.1% syringe
Drug 2	22_Prescription/Injection Order or Administration	YJ Code	2119404G5040	Dobutamine injection 50mg syringe [KKC]
Drug 2	22_Prescription/Injection Order or Administration	YJ Code	2119404G5MID	Generic name code
Drug 2	22_Prescription/Injection Order or Administration	YJ Code	2119404G6020	Dobupum 0.3% injection syringe
Drug 2	22_Prescription/Injection Order or Administration	YJ Code	2119404G6039	Dobupum injection 0.3% syringe
Drug 2	22_Prescription/Injection Order or Administration	YJ Code	2119404G6047	Dobutamine injection 150mg syringe [KKC]
Drug 2	22_Prescription/Injection Order or Administration	YJ Code	2119404G6MID	Generic name code
Drug 2	22_Prescription/Injection Order or Administration	YJ Code	2119404G7027	Dobupum injection 0.6% syringe
Drug 2	22_Prescription/Injection Order or Administration	YJ Code	2119404G7035	Dobutamine injection 300mg syringe [KKC]
Drug 2	22_Prescription/Injection Order or Administration	YJ Code	2119404G7MID	Generic name code
Drug 2	22_Prescription/Injection Order or Administration	YJ Code	2119405D1027	Actosin injection
Drug 2	22_Prescription/Injection Order or Administration	YJ Code	2119405D1035	Actosin injection 300mg
Drug 2	22_Prescription/Injection Order or Administration	YJ Code	2119405D1MID	Generic name code
Drug 2	22_Prescription/Injection Order or Administration	YJ Code	2119406A1025	Amcoral injection 50
Drug 2	22_Prescription/Injection Order or Administration	YJ Code	2119406A1033	Cartonic injection 50mg
Drug 2	22_Prescription/Injection Order or Administration	YJ Code	2119406A1MID	Generic name code
Drug 2	22_Prescription/Injection Order or Administration	YJ Code	2119406A2021	Amcoral injection 100
Drug 2	22_Prescription/Injection Order or Administration	YJ Code	2119406A2030	Cartonic injection 100mg
Drug 2	22_Prescription/Injection Order or Administration	YJ Code	2119406A2MID	Generic name code
Drug 2	22_Prescription/Injection Order or Administration	YJ Code	2119407A1020	Coretec injection 5mg
Drug 2	22_Prescription/Injection Order or Administration	YJ Code	2119407A1MID	Generic name code
Drug 2	22_Prescription/Injection Order or Administration	YJ Code	2119407G1022	Coretec injection SB 9mg
Drug 2	22_Prescription/Injection Order or Administration	YJ Code	2119407G1MID	Generic name code
Drug 2	22_Prescription/Injection Order or Administration	YJ Code	2119408A1024	Milrila injection 10mg
Drug 2	22_Prescription/Injection Order or Administration	YJ Code	2119408A1032	Milrinone injection 10 [KN]
Drug 2	22_Prescription/Injection Order or Administration	YJ Code	2119408A1040	Milrinone injection 10mg [TAKATA]
Drug 2	22_Prescription/Injection Order or Administration	YJ Code	2119408A1059	Milrinone injection 10mg [TAIYO]
Drug 2	22_Prescription/Injection Order or Administration	YJ Code	2119408A1MID	Generic name code
Drug 2	22_Prescription/Injection Order or Administration	YJ Code	2119408A2020	Milrinone injection 10mg [F]
Drug 2	22_Prescription/Injection Order or Administration	YJ Code	2119408A2039	Milrinone injection 10mg [SANDOZ]
Drug 2	22_Prescription/Injection Order or Administration	YJ Code	2119408A2MID	Generic name code
Drug 2	22_Prescription/Injection Order or Administration	YJ Code	2119408A3027	Milrinone injection 22.5mg [F]
Drug 2	22_Prescription/Injection Order or Administration	YJ Code	2119408A3MID	Generic name code
Drug 2	22_Prescription/Injection Order or Administration	YJ Code	2119408G1027	Milrila-K injection 22.5mg
Drug 2	22_Prescription/Injection Order or Administration	YJ Code	2119408G1035	Milrinone injection 22.5mg bag [TAKATA]
Drug 2	22_Prescription/Injection Order or Administration	YJ Code	2119408G1MID	Generic name code
Drug 2	22_Prescription/Injection Order or Administration	YJ Code	2119408G2023	Milrinone injection syringe 10mg [HK]
Drug 2	22_Prescription/Injection Order or Administration	YJ Code	2119408G2MID	Generic name code
Drug 2	22_Prescription/Injection Order or Administration	YJ Code	2119409D1025	Adehl injection
Drug 2	22_Prescription/Injection Order or Administration	YJ Code	2119409D1033	Adehl injection 5mg
Drug 2	22_Prescription/Injection Order or Administration	YJ Code	2119409D1MID	Generic name code
Drug 2	22_Prescription/Injection Order or Administration	YJ Code	2119409D2021	Adehl injection
Drug 2	22_Prescription/Injection Order or Administration	YJ Code	2119409D2030	Adehl injection 10mg
Drug 2	22_Prescription/Injection Order or Administration	YJ Code	2119409D2MID	Generic name code
Drug 2	22_Prescription/Injection Order or Administration	YJ Code	21194A1A1042	Dopamine hydrochloride injection 100mg [TSURUHARA]
Drug 2	22_Prescription/Injection Order or Administration	YJ Code	21194A1A1050	Dopamine hydrochloride injection 100mg [KCC]
Drug 2	22_Prescription/Injection Order or Administration	YJ Code	21194A1A1MID	Generic name code
Drug 2	22_Prescription/Injection Order or Administration	YJ Code	21194A1G1045	Dopamine hydrochloride injection 200mg kit [KCC]
Drug 2	22_Prescription/Injection Order or Administration	YJ Code	21194A1G1MID	Generic name code
Drug 2	22_Prescription/Injection Order or Administration	YJ Code	21194A1G2041	Dopamine hydrochloride injection 600mg kit [KCC]
Drug 2	22_Prescription/Injection Order or Administration	YJ Code	21194A1G2MID	Generic name code
Drug 2	22_Prescription/Injection Order or Administration	YJ Code	21194A2G3026	Dobutamine injection 50mg syringe [KKC]
Drug 2	22_Prescription/Injection Order or Administration	YJ Code	21194A2G3MID	Generic name code
Drug 2	22_Prescription/Injection Order or Administration	YJ Code	21194A2G4022	Dobutamine injection 150mg syringe [KKC]
Drug 2	22_Prescription/Injection Order or Administration	YJ Code	21194A2G4MID	Generic name code
Drug 2	22_Prescription/Injection Order or Administration	YJ Code	21194A2G5029	Dobutamine injection 300mg syringe [KKC]
Drug 2	22_Prescription/Injection Order or Administration	YJ Code	21194A2G5MID	Generic name code
Drug 2	22_Prescription/Injection Order or Administration	YJ Code	2451400A1021	Bosmin injection
Drug 2	22_Prescription/Injection Order or Administration	YJ Code	2451400A1030	Bosmin injection 1mg
Drug 2	22_Prescription/Injection Order or Administration	YJ Code	2451400A1MID	Generic name code
Drug 2	22_Prescription/Injection Order or Administration	YJ Code	2451401A1026	Nor-Adrenaline
Drug 2	22_Prescription/Injection Order or Administration	YJ Code	2451401A1034	Nor-Adrenaline injection 1mg
Drug 2	22_Prescription/Injection Order or Administration	YJ Code	2451401A1MID	Generic name code
Drug 2	22_Prescription/Injection Order or Administration	YJ Code	2451402G1023	Epiquick 0.1% injection syringe
Drug 2	22_Prescription/Injection Order or Administration	YJ Code	2451402G1031	Epinephrine injection 0.1% syringe [TERUMO]
Drug 2	22_Prescription/Injection Order or Administration	YJ Code	2451402G1040	Adrenaline injection 0.1% syringe [TERUMO]
Drug 2	22_Prescription/Injection Order or Administration	YJ Code	2451402G1MID	Generic name code
Drug 2	22_Prescription/Injection Order or Administration	YJ Code	2451402G2020	EpiPen injection 0.15mg
Drug 2	22_Prescription/Injection Order or Administration	YJ Code	2451402G2MID	Generic name code
Drug 2	22_Prescription/Injection Order or Administration	YJ Code	2451402G3026	EpiPen injection 0.3mg
Drug 2	22_Prescription/Injection Order or Administration	YJ Code	2451402G3MID	Generic name code
Drug 2	22_Prescription/Injection Order or Administration	YJ Code	245140AG1029	EpiPen injection 0.3mg
Drug 2	22_Prescription/Injection Order or Administration	YJ Code	245140AG1MID	Generic name code
Drug 2	22_Prescription/Injection Order or Administration	YJ Code	245140AG2025	EpiPen injection 0.15mg
Drug 2	22_Prescription/Injection Order or Administration	YJ Code	245140AG2MID	Generic name code
Drug 2	23_DPC or Receipt Pharmaceutical Information	Electronic medical claims processing code	620001305	Dopamine injection 2% [KN] 100mg 5ml
Drug 2	23_DPC or Receipt Pharmaceutical Information	Electronic medical claims processing code	620002174	Inovan injection 50mg 2.5ml
Drug 2	23_DPC or Receipt Pharmaceutical Information	Electronic medical claims processing code	620002175	Inovan injection 100mg 5ml
Drug 2	23_DPC or Receipt Pharmaceutical Information	Electronic medical claims processing code	620002176	Inovan injection 200mg 10ml
Drug 2	23_DPC or Receipt Pharmaceutical Information	Electronic medical claims processing code	620002179	Dopamine hydrochloride injection kit 200 0.1% 200ml
Drug 2	23_DPC or Receipt Pharmaceutical Information	Electronic medical claims processing code	620002180	Dopamine hydrochloride injection kit 600 0.3% 200ml
Drug 2	23_DPC or Receipt Pharmaceutical Information	Electronic medical claims processing code	620002181	Dobutamine hydrochloride injection 100mg
Drug 2	23_DPC or Receipt Pharmaceutical Information	Electronic medical claims processing code	620002437	Coretec injection SB 9mg 150ml
Drug 2	23_DPC or Receipt Pharmaceutical Information	Electronic medical claims processing code	620002566	Epinephrine injection 0.1% syringe [TERUMO] 1ml
Drug 2	23_DPC or Receipt Pharmaceutical Information	Electronic medical claims processing code	620003097	Doparalmin injection 100mg 5ml
Drug 2	23_DPC or Receipt Pharmaceutical Information	Electronic medical claims processing code	620003194	Inovan injection 0.1% syringe 50ml
Drug 2	23_DPC or Receipt Pharmaceutical Information	Electronic medical claims processing code	620003195	Inovan injection 0.3% syringe 50ml
Drug 2	23_DPC or Receipt Pharmaceutical Information	Electronic medical claims processing code	620003204	Kakodin-D injection 0.1% 200ml
Drug 2	23_DPC or Receipt Pharmaceutical Information	Electronic medical claims processing code	620003205	Kakodin D-injection 0.1% 200ml
Drug 2	23_DPC or Receipt Pharmaceutical Information	Electronic medical claims processing code	620003206	Kakodin D-injection 0.3% 200ml
Drug 2	23_DPC or Receipt Pharmaceutical Information	Electronic medical claims processing code	620003207	Kakodin D-injection 0.3% 200ml
Drug 2	23_DPC or Receipt Pharmaceutical Information	Electronic medical claims processing code	620003208	Catabon Hi injection 600mg 0.3% 200ml
Drug 2	23_DPC or Receipt Pharmaceutical Information	Electronic medical claims processing code	620003209	Catabon Low injection 200mg 0.1% 200ml
Drug 2	23_DPC or Receipt Pharmaceutical Information	Electronic medical claims processing code	620003223	Dobutrex injection 200mg bag 0.1% 200ml
Drug 2	23_DPC or Receipt Pharmaceutical Information	Electronic medical claims processing code	620003224	Dobutrex injection 600mg bag 0.3% 200ml
Drug 2	23_DPC or Receipt Pharmaceutical Information	Electronic medical claims processing code	620003225	Dobupum injection 0.1% syringe 50ml
Drug 2	23_DPC or Receipt Pharmaceutical Information	Electronic medical claims processing code	620003226	Dobupum injection 0.3% syringe 50ml
Drug 2	23_DPC or Receipt Pharmaceutical Information	Electronic medical claims processing code	620003227	Dopmin injection 100mg
Drug 2	23_DPC or Receipt Pharmaceutical Information	Electronic medical claims processing code	620003427	Kakodin injection 100mg 5ml
Drug 2	23_DPC or Receipt Pharmaceutical Information	Electronic medical claims processing code	620003428	Gabans injection 100mg 5ml
Drug 2	23_DPC or Receipt Pharmaceutical Information	Electronic medical claims processing code	620003769	Dominin injection 40mg 2ml
Drug 2	23_DPC or Receipt Pharmaceutical Information	Electronic medical claims processing code	620003770	Dominin injection 100mg 5ml
Drug 2	23_DPC or Receipt Pharmaceutical Information	Electronic medical claims processing code	620003771	Dominin injection 200mg 10ml
Drug 2	23_DPC or Receipt Pharmaceutical Information	Electronic medical claims processing code	620003824	Milrinone injection syringe 10mg [HK] 10ml
Drug 2	23_DPC or Receipt Pharmaceutical Information	Electronic medical claims processing code	620004105	Inovan injection 0.6% syringe 50ml
Drug 2	23_DPC or Receipt Pharmaceutical Information	Electronic medical claims processing code	620004160	Dopamine liquid 600 [TOWA] 0.3% 200ml
Drug 2	23_DPC or Receipt Pharmaceutical Information	Electronic medical claims processing code	620004161	Dobupum injection 0.6% syringe 50ml
Drug 2	23_DPC or Receipt Pharmaceutical Information	Electronic medical claims processing code	620004201	Milrinone injection 10mg [F] 10ml
Drug 2	23_DPC or Receipt Pharmaceutical Information	Electronic medical claims processing code	620004202	Milrinone injection 22.5mg [F] 150ml
Drug 2	23_DPC or Receipt Pharmaceutical Information	Electronic medical claims processing code	620004203	Milrinone injection [KN] 10mg 10ml
Drug 2	23_DPC or Receipt Pharmaceutical Information	Electronic medical claims processing code	620004204	Milrinone injection 10mg [TAKATA] 10ml
Drug 2	23_DPC or Receipt Pharmaceutical Information	Electronic medical claims processing code	620004205	Milrinone injection 22.5mg bag [TAKATA] 150ml
Drug 2	23_DPC or Receipt Pharmaceutical Information	Electronic medical claims processing code	620004410	Martburn injection 100mg 5ml
Drug 2	23_DPC or Receipt Pharmaceutical Information	Electronic medical claims processing code	620004749	Bubucin injection 100mg
Drug 2	23_DPC or Receipt Pharmaceutical Information	Electronic medical claims processing code	620005187	Dobutamine injection 100mg [IROM]
Drug 2	23_DPC or Receipt Pharmaceutical Information	Electronic medical claims processing code	620005188	Dobutrex kit injection 200mg 0.1% 200ml
Drug 2	23_DPC or Receipt Pharmaceutical Information	Electronic medical claims processing code	620005189	Dobutrex kit injection 600mg 0.3% 200ml
Drug 2	23_DPC or Receipt Pharmaceutical Information	Electronic medical claims processing code	620005804	Dopamine hydrochloride injection 100mg [IROM] 5ml
Drug 2	23_DPC or Receipt Pharmaceutical Information	Electronic medical claims processing code	620005858	Dopamine hydrochloride injection 100mg [TAIYO] 5ml
Drug 2	23_DPC or Receipt Pharmaceutical Information	Electronic medical claims processing code	620006754	Dobutamine hydrochloride 100mg injection
Drug 2	23_DPC or Receipt Pharmaceutical Information	Electronic medical claims processing code	620007163	Actosin injection 300mg (with solution)
Drug 2	23_DPC or Receipt Pharmaceutical Information	Electronic medical claims processing code	620007387	Doputamin injection 100mg
Drug 2	23_DPC or Receipt Pharmaceutical Information	Electronic medical claims processing code	620007388	Dobux injection 100mg
Drug 2	23_DPC or Receipt Pharmaceutical Information	Electronic medical claims processing code	620007550	Retamex injection 100mg
Drug 2	23_DPC or Receipt Pharmaceutical Information	Electronic medical claims processing code	620008233	Milrinone injection 10mg [TAIYO] 10ml
Drug 2	23_DPC or Receipt Pharmaceutical Information	Electronic medical claims processing code	620008380	Dopamine hydrochloride injection 50mg [KN] 2.5ml
Drug 2	23_DPC or Receipt Pharmaceutical Information	Electronic medical claims processing code	620008381	Dopamine hydrochloride injection 100mg [KN] 5ml
Drug 2	23_DPC or Receipt Pharmaceutical Information	Electronic medical claims processing code	620008382	Dopamine hydrochloride injection 200mg [KN] 10ml
Drug 2	23_DPC or Receipt Pharmaceutical Information	Electronic medical claims processing code	620008384	Nor-Adrenaline injection 1mg 0.1% 1ml
Drug 2	23_DPC or Receipt Pharmaceutical Information	Electronic medical claims processing code	620008402	Yaelista injection 100mg 5ml
Drug 2	23_DPC or Receipt Pharmaceutical Information	Electronic medical claims processing code	620008926	Proternol-L injection 0.2mg 0.02% 1ml

Drug 2	23_DPC or Receipt Pharmaceutical Information	Electronic medical claims processing code	620008927	Proternol-L injection 1mg 0.02% 5ml
Drug 2	23_DPC or Receipt Pharmaceutical Information	Electronic medical claims processing code	620009260	Evetant injection 100mg 5ml
Drug 2	23_DPC or Receipt Pharmaceutical Information	Electronic medical claims processing code	620244701	Dopamine hydrochloride injection 100mg [ISEI] 5ml
Drug 2	23_DPC or Receipt Pharmaceutical Information	Electronic medical claims processing code	620244702	Dopamine hydrochloride injection 100mg [TSURUHARA] 5ml
Drug 2	23_DPC or Receipt Pharmaceutical Information	Electronic medical claims processing code	620244707	Dopamine hydrochloride injection 100mg [KCC] 5ml
Drug 2	23_DPC or Receipt Pharmaceutical Information	Electronic medical claims processing code	620244711	Critpan injection 100mg 5ml
Drug 2	23_DPC or Receipt Pharmaceutical Information	Electronic medical claims processing code	620244718	Dopamine hydrochloride injection 100mg [NP] 5ml
Drug 2	23_DPC or Receipt Pharmaceutical Information	Electronic medical claims processing code	620244722	Tsurudopami injection 100mg 5ml
Drug 2	23_DPC or Receipt Pharmaceutical Information	Electronic medical claims processing code	620244732	Dopamine hydrochloride injection 100mg [PFIZER] 5ml
Drug 2	23_DPC or Receipt Pharmaceutical Information	Electronic medical claims processing code	620244734	Dopamine hydrochloride injection 100mg [NIG] 5ml
Drug 2	23_DPC or Receipt Pharmaceutical Information	Electronic medical claims processing code	620245102	Tsurudopami injection 200mg 10ml
Drug 2	23_DPC or Receipt Pharmaceutical Information	Electronic medical claims processing code	620246104	Dopamine hydrochloride injection 200mg kit [PFIZER] 0.1% 200ml
Drug 2	23_DPC or Receipt Pharmaceutical Information	Electronic medical claims processing code	620246201	Dopamine hydrochloride injection 200mg bag [NICHIYAKU] 0.1% 200ml
Drug 2	23_DPC or Receipt Pharmaceutical Information	Electronic medical claims processing code	620246305	Dopamine hydrochloride injection 200mg bag [TAKEDA TEVA] 0.1% 200ml
Drug 2	23_DPC or Receipt Pharmaceutical Information	Electronic medical claims processing code	620246306	Dopamine hydrochloride injection 200mg bag [NIG] 0.1% 200ml
Drug 2	23_DPC or Receipt Pharmaceutical Information	Electronic medical claims processing code	620246404	Dopamine hydrochloride injection 600mg kit [PFIZER] 0.3% 200ml
Drug 2	23_DPC or Receipt Pharmaceutical Information	Electronic medical claims processing code	620246501	Dopamine hydrochloride injection 600mg bag [NICHIYAKU] 0.3% 200ml
Drug 2	23_DPC or Receipt Pharmaceutical Information	Electronic medical claims processing code	620246605	Dopamine hydrochloride injection 600mg bag [TAKEDA TEVA] 0.3% 200ml
Drug 2	23_DPC or Receipt Pharmaceutical Information	Electronic medical claims processing code	620246606	Dopamine hydrochloride injection 600mg bag [NIG] 0.3% 200ml
Drug 2	23_DPC or Receipt Pharmaceutical Information	Electronic medical claims processing code	620247903	Dobutamine injection 200mg kit [PFIZER] 0.1% 200ml
Drug 2	23_DPC or Receipt Pharmaceutical Information	Electronic medical claims processing code	620248003	Dobutamine injection 600mg kit [PFIZER] 0.3% 200ml
Drug 2	23_DPC or Receipt Pharmaceutical Information	Electronic medical claims processing code	620248901	Adehl injection 5mg
Drug 2	23_DPC or Receipt Pharmaceutical Information	Electronic medical claims processing code	620249001	Adehl injection 10mg
Drug 2	23_DPC or Receipt Pharmaceutical Information	Electronic medical claims processing code	620517902	Bosmin injection 1mg 0.1% 1ml
Drug 2	23_DPC or Receipt Pharmaceutical Information	Electronic medical claims processing code	621365306	Dobutamine injection 100mg [AFP]
Drug 2	23_DPC or Receipt Pharmaceutical Information	Electronic medical claims processing code	621365314	Dobutamine hydrochloride injection 100mg [SAWAI]
Drug 2	23_DPC or Receipt Pharmaceutical Information	Electronic medical claims processing code	621365316	Dobutamine injection 100mg [F]
Drug 2	23_DPC or Receipt Pharmaceutical Information	Electronic medical claims processing code	621365317	Dobutamine hydrochloride injection 100mg [KN]
Drug 2	23_DPC or Receipt Pharmaceutical Information	Electronic medical claims processing code	621365321	Dobutamine injection 100mg [PFIZER]
Drug 2	23_DPC or Receipt Pharmaceutical Information	Electronic medical claims processing code	621371901	Adrenaline injection 0.1% syringe [TERUMO] 1ml
Drug 2	23_DPC or Receipt Pharmaceutical Information	Electronic medical claims processing code	621399003	Critpan injection 50mg 2.5ml
Drug 2	23_DPC or Receipt Pharmaceutical Information	Electronic medical claims processing code	621399005	Evetant injection 50mg 2.5ml
Drug 2	23_DPC or Receipt Pharmaceutical Information	Electronic medical claims processing code	621399006	Dopamine hydrochloride injection 50mg [IROM] 2.5ml
Drug 2	23_DPC or Receipt Pharmaceutical Information	Electronic medical claims processing code	621399007	Yaelista injection 50mg 2.5ml
Drug 2	23_DPC or Receipt Pharmaceutical Information	Electronic medical claims processing code	621399008	Dopamine hydrochloride injection 50mg [TAIYO] 2.5ml
Drug 2	23_DPC or Receipt Pharmaceutical Information	Electronic medical claims processing code	621399009	Martburn injection 50mg 2.5ml
Drug 2	23_DPC or Receipt Pharmaceutical Information	Electronic medical claims processing code	621399010	Kakodin injection 50mg 2.5ml
Drug 2	23_DPC or Receipt Pharmaceutical Information	Electronic medical claims processing code	621399011	Tsurudopami injection 50mg 2.5ml
Drug 2	23_DPC or Receipt Pharmaceutical Information	Electronic medical claims processing code	621399012	Doparalmin injection 50mg 2.5ml
Drug 2	23_DPC or Receipt Pharmaceutical Information	Electronic medical claims processing code	621399013	Dopamine hydrochloride injection 50mg [NP] 2.5ml
Drug 2	23_DPC or Receipt Pharmaceutical Information	Electronic medical claims processing code	621399014	Dopamine hydrochloride injection 50mg [NIG] 2.5ml
Drug 2	23_DPC or Receipt Pharmaceutical Information	Electronic medical claims processing code	621512001	Dobutamine injection 50mg syringe [KKC] 0.1% 50ml
Drug 2	23_DPC or Receipt Pharmaceutical Information	Electronic medical claims processing code	621512101	Dobutamine injection 150mg syringe [KKC] 0.3% 50ml
Drug 2	23_DPC or Receipt Pharmaceutical Information	Electronic medical claims processing code	621644502	Dopamine hydrochloride injection 200mg kit [KCC] 0.1% 200ml
Drug 2	23_DPC or Receipt Pharmaceutical Information	Electronic medical claims processing code	621644602	Dopamine hydrochloride injection 600mg kit [KCC] 0.3% 200ml
Drug 2	23_DPC or Receipt Pharmaceutical Information	Electronic medical claims processing code	621757901	Dobutamine injection 300mg syringe [KKC] 0.6% 50ml
Drug 2	23_DPC or Receipt Pharmaceutical Information	Electronic medical claims processing code	621958101	Critpan injection 200mg 10ml
Drug 2	23_DPC or Receipt Pharmaceutical Information	Electronic medical claims processing code	621967601	Milrinone injection 10mg [SANDOZ] 10ml
Drug 2	23_DPC or Receipt Pharmaceutical Information	Electronic medical claims processing code	622014401	Evetant injection 200mg 10ml
Drug 2	23_DPC or Receipt Pharmaceutical Information	Electronic medical claims processing code	622033601	Doparalmin injection 200mg 10ml
Drug 2	23_DPC or Receipt Pharmaceutical Information	Electronic medical claims processing code	622033602	Dopamine hydrochloride injection 200mg [NP] 10ml
Drug 2	23_DPC or Receipt Pharmaceutical Information	Electronic medical claims processing code	622043701	Dopamine hydrochloride injection 200mg [IROM] 10ml
Drug 2	23_DPC or Receipt Pharmaceutical Information	Electronic medical claims processing code	622051801	Yaelista injection 200mg 10ml
Drug 2	23_DPC or Receipt Pharmaceutical Information	Electronic medical claims processing code	622060501	Dopamine hydrochloride injection 200mg [TAIYO] 10ml
Drug 2	23_DPC or Receipt Pharmaceutical Information	Electronic medical claims processing code	622060503	Dopamine hydrochloride injection 200mg [NIG] 10ml
Drug 2	23_DPC or Receipt Pharmaceutical Information	Electronic medical claims processing code	622067301	Martburn injection 200mg 10ml
Drug 2	23_DPC or Receipt Pharmaceutical Information	Electronic medical claims processing code	622084701	Kakodin injection 200mg 10ml
Drug 2	23_DPC or Receipt Pharmaceutical Information	Electronic medical claims processing code	622749400	Dopamine hydrochloride 50mg 2.5ml injection
Drug 2	23_DPC or Receipt Pharmaceutical Information	Electronic medical claims processing code	622749500	Dopamine hydrochloride 0.1% 200ml kit
Drug 2	23_DPC or Receipt Pharmaceutical Information	Electronic medical claims processing code	622749600	Dopamine hydrochloride 0.3% 200m lkit
Drug 2	23_DPC or Receipt Pharmaceutical Information	Electronic medical claims processing code	622749700	Dobutamine hydrochloride 100mg injection
Drug 2	23_DPC or Receipt Pharmaceutical Information	Electronic medical claims processing code	622749800	Dobutamine hydrochloride 0.1% 200ml kit
Drug 2	23_DPC or Receipt Pharmaceutical Information	Electronic medical claims processing code	622749900	Dobutamine hydrochloride 0.3% 200m lkit
Drug 2	23_DPC or Receipt Pharmaceutical Information	Electronic medical claims processing code	622750000	Dobutamine hydrochloride 0.1% 50ml kit
Drug 2	23_DPC or Receipt Pharmaceutical Information	Electronic medical claims processing code	622750100	Dobutamine hydrochloride 0.3% 50m lkit
Drug 2	23_DPC or Receipt Pharmaceutical Information	Electronic medical claims processing code	622750200	Dobutamine hydrochloride 0.6% 50ml kit
Drug 2	23_DPC or Receipt Pharmaceutical Information	Electronic medical claims processing code	622750300	Milrinone 10mg 10ml injection
Drug 2	23_DPC or Receipt Pharmaceutical Information	Electronic medical claims processing code	622750400	Milrinone 22.5mg 150ml injection
Drug 2	23_DPC or Receipt Pharmaceutical Information	Electronic medical claims processing code	622750500	Milrinone 22.5mg 150ml kit
Drug 2	23_DPC or Receipt Pharmaceutical Information	Electronic medical claims processing code	628704702	EpiPen injection 0.15mg
Drug 2	23_DPC or Receipt Pharmaceutical Information	Electronic medical claims processing code	628704802	EpiPen injection 0.3mg
Drug 2	23_DPC or Receipt Pharmaceutical Information	Electronic medical claims processing code	640406128	Doputamin H injection 100mg
Drug 2	23_DPC or Receipt Pharmaceutical Information	Electronic medical claims processing code	640406181	Generic dobutamine hydrochloride injection 100mg (discontinued drug)
Drug 2	23_DPC or Receipt Pharmaceutical Information	Electronic medical claims processing code	640407199	Dopmin Kinjection 200 0.1% 200ml
Drug 2	23_DPC or Receipt Pharmaceutical Information	Electronic medical claims processing code	640407200	Dopmin Kinjection 600 0.3% 200ml
Drug 2	23_DPC or Receipt Pharmaceutical Information	Electronic medical claims processing code	640408079	Coretec injection 5mg 5ml
Drug 2	23_DPC or Receipt Pharmaceutical Information	Electronic medical claims processing code	640408133	Milrila injection 10mg 10ml
Drug 2	23_DPC or Receipt Pharmaceutical Information	Electronic medical claims processing code	640421064	Milrila-K injection 22.5mg 150ml
Drug 2	23_DPC or Receipt Pharmaceutical Information	Electronic medical claims processing code	640421068	Adehl injection 5mg
Drug 2	23_DPC or Receipt Pharmaceutical Information	Electronic medical claims processing code	640421069	Adehl injection 10mg
Drug 2	23_DPC or Receipt Pharmaceutical Information	Electronic medical claims processing code	640421089	Dopamine liquid 200 [TOWA] 0.1% 200ml
Drug 2	23_DPC or Receipt Pharmaceutical Information	Electronic medical claims processing code	640421090	Dopamine liquid 600 [TOWA] 0.3% 200ml
Drug 2	23_DPC or Receipt Pharmaceutical Information	Electronic medical claims processing code	640431041	Taiadopa injection 100mg 5ml
Drug 2	23_DPC or Receipt Pharmaceutical Information	Electronic medical claims processing code	640461007	Dopamine hydrochloride 50mg 2.5ml injection
Drug 2	23_DPC or Receipt Pharmaceutical Information	Electronic medical claims processing code	640461008	Dopamine hydrochloride 100mg 5ml injection
Drug 2	23_DPC or Receipt Pharmaceutical Information	Electronic medical claims processing code	640461009	Dopamine hydrochloride injection 200mg 5ml
Drug 2	23_DPC or Receipt Pharmaceutical Information	Electronic medical claims processing code	640461010	Dopamine hydrochloride 200mg 10ml injection
Drug 2	23_DPC or Receipt Pharmaceutical Information	Electronic medical claims processing code	642110041	Proternol-L injection 0.02% 1ml
Drug 2	23_DPC or Receipt Pharmaceutical Information	Electronic medical claims processing code	642110049	Catherine injection 100mg 5ml
Drug 2	23_DPC or Receipt Pharmaceutical Information	Electronic medical claims processing code	642110050	Critpan injection 100mg 5ml
Drug 2	23_DPC or Receipt Pharmaceutical Information	Electronic medical claims processing code	642110052	Tronjin injection 100mg 5ml
Drug 2	23_DPC or Receipt Pharmaceutical Information	Electronic medical claims processing code	642110058	Proternol-L injection 0.02% 5ml
Drug 2	23_DPC or Receipt Pharmaceutical Information	Electronic medical claims processing code	642110071	Actosin injection 300mg (with solution)
Drug 2	23_DPC or Receipt Pharmaceutical Information	Electronic medical claims processing code	642110074	Actopamin injection 100mg 5ml
Drug 2	23_DPC or Receipt Pharmaceutical Information	Electronic medical claims processing code	642110075	Dopamine hydrochloride injection [KOBAYASHI] 100mg 5ml
Drug 2	23_DPC or Receipt Pharmaceutical Information	Electronic medical claims processing code	642110080	Tsurudopami injection 100mg 5ml
Drug 2	23_DPC or Receipt Pharmaceutical Information	Electronic medical claims processing code	642110081	Tsurudopami injection 200mg 10ml
Drug 2	23_DPC or Receipt Pharmaceutical Information	Electronic medical claims processing code	642110084	Dobutrex injection 100mg
Drug 2	23_DPC or Receipt Pharmaceutical Information	Electronic medical claims processing code	642110085	Dolpamil injection 100mg 5ml
Drug 2	23_DPC or Receipt Pharmaceutical Information	Electronic medical claims processing code	642110088	Martburn injection 100mg 5ml
Drug 2	23_DPC or Receipt Pharmaceutical Information	Electronic medical claims processing code	642110090	Evetant injection 100mg 5ml
Drug 2	23_DPC or Receipt Pharmaceutical Information	Electronic medical claims processing code	642110096	Yaelista injection 100mg 5ml
Drug 2	23_DPC or Receipt Pharmaceutical Information	Electronic medical claims processing code	642110110	Retamex 100mg
Drug 2	23_DPC or Receipt Pharmaceutical Information	Electronic medical claims processing code	642110125	Dopamine hydrochloride injection kit 200 0.1% 200ml
Drug 2	23_DPC or Receipt Pharmaceutical Information	Electronic medical claims processing code	642110126	Dopamine hydrochloride injection kit 600 0.3% 200ml
Drug 2	23_DPC or Receipt Pharmaceutical Information	Electronic medical claims processing code	642110127	Generic dobutamine hydrochloride injection 100mg (discontinued drug)
Drug 2	23_DPC or Receipt Pharmaceutical Information	Electronic medical claims processing code	642110130	Dobux injection 100mg
Drug 2	23_DPC or Receipt Pharmaceutical Information	Electronic medical claims processing code	642110133	Predopa injection 200 0.1% 200ml
Drug 2	23_DPC or Receipt Pharmaceutical Information	Electronic medical claims processing code	642110134	Predopa injection 600 0.3% 200ml
Drug 2	23_DPC or Receipt Pharmaceutical Information	Electronic medical claims processing code	642110135	Amcoral injection 50 0.5% 10ml
Drug 2	23_DPC or Receipt Pharmaceutical Information	Electronic medical claims processing code	642110136	Amcoral injection 100 0.5% 20ml
Drug 2	23_DPC or Receipt Pharmaceutical Information	Electronic medical claims processing code	642110138	Cartonic injection 100mg 0.5% 20ml
Drug 2	23_DPC or Receipt Pharmaceutical Information	Electronic medical claims processing code	642450005	Adrenaline injection 0.1% 1ml
Drug 2	23_DPC or Receipt Pharmaceutical Information	Electronic medical claims processing code	642450071	Nor-Adrenaline injection 0.1% 1ml
Drug 2	23_DPC or Receipt Pharmaceutical Information	Electronic medical claims processing code	642450164	Bosmin injection 0.1% 1ml
Drug 2	23_DPC or Receipt Pharmaceutical Information	Electronic medical claims processing code	642450165	Nor-Adrenaline 0.1% 1ml
Drug 2	22_Prescription/Injection Order or Administration	YJ Code	2119003C1070	Neucul granule
Drug 2	22_Prescription/Injection Order or Administration	YJ Code	2119003C1MID	Generic name code
Drug 2	22_Prescription/Injection Order or Administration	YJ Code	2119003C2026	Generic ubidecarenone granule 20 (discontinued drug)
Drug 2	22_Prescription/Injection Order or Administration	YJ Code	2119003C2MID	Generic name code
Drug 2	22_Prescription/Injection Order or Administration	YJ Code	2119003D1025	Inokiten granule
Drug 2	22_Prescription/Injection Order or Administration	YJ Code	2119003D1050	Tridemin G
Drug 2	22_Prescription/Injection Order or Administration	YJ Code	2119003D1068	Neuquinon granule
Drug 2	22_Prescription/Injection Order or Administration	YJ Code	2119003D1122	Yube-Q granule
Drug 2	22_Prescription/Injection Order or Administration	YJ Code	2119003D1130	Tridemin granule 1%
Drug 2	22_Prescription/Injection Order or Administration	YJ Code	2119003D1149	Neuquinon granule 1%
Drug 2	22_Prescription/Injection Order or Administration	YJ Code	2119003D1157	Inokiten granule 1%
Drug 2	22_Prescription/Injection Order or Administration	YJ Code	2119003D1165	Yube-Q granule
Drug 2	22_Prescription/Injection Order or Administration	YJ Code	2119003D1173	Ubidecarenone granule 1% [TSURUHARA]
Drug 2	22_Prescription/Injection Order or Administration	YJ Code	2119003D1MID	Generic name code
Drug 2	22_Prescription/Injection Order or Administration	YJ Code	2119003D2030	Enazym granule (2%)
Drug 2	22_Prescription/Injection Order or Administration	YJ Code	2119003D2MID	Generic name code
Drug 2	22_Prescription/Injection Order or Administration	YJ Code	2119003F1042	Udekinon tablet5mg
Drug 2	22_Prescription/Injection Order or Administration	YJ Code	2119003F1093	Neuquinon tablet 5mg
Drug 2	22_Prescription/Injection Order or Administration	YJ Code	2119003F1115	Adelir tablet 5
Drug 2	22_Prescription/Injection Order or Administration	YJ Code	2119003F1123	Kaitron tablet 5mg
Drug 2	22_Prescription/Injection Order or Administration	YJ Code	2119003F1131	Ubidecarenone tablet 5mg [SAWAI]
Drug 2	22_Prescription/Injection Order or Administration	YJ Code	2119003F1MID	Generic name code
Drug 2	22_Prescription/Injection Order or Administration	YJ Code	2119003F2022	Generic ubidecarenone tablet 10 (discontinued drug)
Drug 2	22_Prescription/Injection Order or Administration	YJ Code	2119003F2030	Adelir tablet 10
Drug 2	22_Prescription/Injection Order or Administration	YJ Code	2119003F2057	Inokiten
Drug 2	22_Prescription/Injection Order or Administration	YJ Code	2119003F2065	Udekinon tablet 10mg
Drug 2	22_Prescription/Injection Order or Administration	YJ Code	2119003F2073	Emitolon tablet
Drug 2	22_Prescription/Injection Order or Administration	YJ Code	2119003F2103	Omquinone tablet 10mg
Drug 2	22_Prescription/Injection Order or Administration	YJ Code	2119003F2111	Kaitron-T 10
Drug 2	22_Prescription/Injection Order or Administration	YJ Code	2119003F2138	Cardic Q tablet 10mg
Drug 2	22_Prescription/Injection Order or Administration	YJ Code	2119003F2146	Carbiquinone tablet
Drug 2	22_Prescription/Injection Order or Administration	YJ Code	2119003F2162	Generic ubidecarenone tablet 10mg (discontinued drug)
Drug 2	22_Prescription/Injection Order or Administration	YJ Code	2119003F2189	Coloquinon tablet 10mg

Drug 2	22_Prescription/Injection Order or Administration	YJ Code	2119003F2197	Sanquinone tablet 10
Drug 2	22_Prescription/Injection Order or Administration	YJ Code	2119003F2219	Justquinon tablet 10
Drug 2	22_Prescription/Injection Order or Administration	YJ Code	2119003F2227	Sukinon 10
Drug 2	22_Prescription/Injection Order or Administration	YJ Code	2119003F2260	Soranekinon tablet 10
Drug 2	22_Prescription/Injection Order or Administration	YJ Code	2119003F2308	Decantoin tablet 10
Drug 2	22_Prescription/Injection Order or Administration	YJ Code	2119003F2316	Tridemin
Drug 2	22_Prescription/Injection Order or Administration	YJ Code	2119003F2324	Generic ubidecarenone tablet 10 (discontinued drug)
Drug 2	22_Prescription/Injection Order or Administration	YJ Code	2119003F2332	Neuquinon tablet 10mg
Drug 2	22_Prescription/Injection Order or Administration	YJ Code	2119003F2340	Neuquinon tablet 10mg
Drug 2	22_Prescription/Injection Order or Administration	YJ Code	2119003F2359	Generic ubidecarenone tablet 10 (discontinued drug)
Drug 2	22_Prescription/Injection Order or Administration	YJ Code	2119003F2367	Heartcin tablet 10
Drug 2	22_Prescription/Injection Order or Administration	YJ Code	2119003F2430	Frupanon tablet
Drug 2	22_Prescription/Injection Order or Administration	YJ Code	2119003F2472	Ubidecarenone tablet 10 [HOKUEI]
Drug 2	22_Prescription/Injection Order or Administration	YJ Code	2119003F2502	Yube-Q
Drug 2	22_Prescription/Injection Order or Administration	YJ Code	2119003F2510	Youbiquinon tablet 10
Drug 2	22_Prescription/Injection Order or Administration	YJ Code	2119003F2529	Lacobile tablet
Drug 2	22_Prescription/Injection Order or Administration	YJ Code	2119003F2537	Rasanen tablet 10
Drug 2	22_Prescription/Injection Order or Administration	YJ Code	2119003F2545	Generic ubidecarenone tablet 10 (discontinued drug)
Drug 2	22_Prescription/Injection Order or Administration	YJ Code	2119003F2553	Frupanon tablet 10mg
Drug 2	22_Prescription/Injection Order or Administration	YJ Code	2119003F2561	Tridemin tablet 10mg
Drug 2	22_Prescription/Injection Order or Administration	YJ Code	2119003F2570	Kaitron tablet 10mg
Drug 2	22_Prescription/Injection Order or Administration	YJ Code	2119003F2588	Inokiten tablet 10mg
Drug 2	22_Prescription/Injection Order or Administration	YJ Code	2119003F2596	Ubidecarenone tablet 10mg [TOWA]
Drug 2	22_Prescription/Injection Order or Administration	YJ Code	2119003F2600	Yube-Q tablet
Drug 2	22_Prescription/Injection Order or Administration	YJ Code	2119003F2618	Carbiquinone tablet 10mg
Drug 2	22_Prescription/Injection Order or Administration	YJ Code	2119003F2626	Lacobile tablet 10mg
Drug 2	22_Prescription/Injection Order or Administration	YJ Code	2119003F2634	Ubidecarenone tablet 10mg [NISSIN]
Drug 2	22_Prescription/Injection Order or Administration	YJ Code	2119003F2642	Ubidecarenone tablet 10mg [TSURUHARA]
Drug 2	22_Prescription/Injection Order or Administration	YJ Code	2119003F2650	Ubidecarenone tablet 10mg [SAWAI]
Drug 2	22_Prescription/Injection Order or Administration	YJ Code	2119003F2669	Ubidecarenone tablet 10mg [ISEI]
Drug 2	22_Prescription/Injection Order or Administration	YJ Code	2119003F2MID	Generic name code
Drug 2	22_Prescription/Injection Order or Administration	YJ Code	2119003M1033	Inokiten capsule
Drug 2	22_Prescription/Injection Order or Administration	YJ Code	2119003M1084	Generic ubidecarenone tablet (discontinued drug)
Drug 2	22_Prescription/Injection Order or Administration	YJ Code	2119003M1122	Neuquinon capsule
Drug 2	22_Prescription/Injection Order or Administration	YJ Code	2119003M1165	Yube-Q
Drug 2	22_Prescription/Injection Order or Administration	YJ Code	2119003M1173	Youbiquinon [capsule]
Drug 2	22_Prescription/Injection Order or Administration	YJ Code	2119003M1203	Neuquinon capsule 5mg
Drug 2	22_Prescription/Injection Order or Administration	YJ Code	2119003M1211	Inokiten capsule 5mg
Drug 2	22_Prescription/Injection Order or Administration	YJ Code	2119003M1220	Yube-Q capsule 5mg
Drug 2	22_Prescription/Injection Order or Administration	YJ Code	2119003M1238	Ubidecarenone capsule 5mg [TOWA]
Drug 2	22_Prescription/Injection Order or Administration	YJ Code	2119003M1246	Carbiquinone capsule 5mg
Drug 2	22_Prescription/Injection Order or Administration	YJ Code	2119003M1254	Ubidecarenone capsule 5mg [KYORIN]
Drug 2	22_Prescription/Injection Order or Administration	YJ Code	2119003M1262	Ubequinon capsule 5mg
Drug 2	22_Prescription/Injection Order or Administration	YJ Code	2119003M1270	Tridemin capsule 5mg
Drug 2	22_Prescription/Injection Order or Administration	YJ Code	2119003M1289	Ubidecarenone capsule 5mg [NISSIN]
Drug 2	22_Prescription/Injection Order or Administration	YJ Code	2119003M1297	Ubidecarenone capsule 5mg [TSURUHARA]
Drug 2	22_Prescription/Injection Order or Administration	YJ Code	2119003M1MID	Generic name code
Drug 2	22_Prescription/Injection Order or Administration	YJ Code	2119003M2030	Udekinon capsule 10mg
Drug 2	22_Prescription/Injection Order or Administration	YJ Code	2119003M2048	Enazymcapsule (10mg)
Drug 2	22_Prescription/Injection Order or Administration	YJ Code	2119003M2072	Dekasoft
Drug 2	22_Prescription/Injection Order or Administration	YJ Code	2119003M2099	Tokeel capsule 10
Drug 2	22_Prescription/Injection Order or Administration	YJ Code	2119003M2102	Neucul capsule 10
Drug 2	22_Prescription/Injection Order or Administration	YJ Code	2119003M2161	Ubequinon [capsule]
Drug 2	22_Prescription/Injection Order or Administration	YJ Code	2119003M2218	Ubequinon capsule 10mg
Drug 2	22_Prescription/Injection Order or Administration	YJ Code	2119003M2226	Dekasoft capsule 10mg
Drug 2	22_Prescription/Injection Order or Administration	YJ Code	2119003M2234	Ubidecarenone capsule 10mg [KYORIN]
Drug 2	22_Prescription/Injection Order or Administration	YJ Code	2119003M2MID	Generic name code
Drug 2	22_Prescription/Injection Order or Administration	YJ Code	2119004C1024	Kalgut granule
Drug 2	22_Prescription/Injection Order or Administration	YJ Code	2119004C1032	Kalgut granule 5%
Drug 2	22_Prescription/Injection Order or Administration	YJ Code	2119004C1MID	Generic name code
Drug 2	22_Prescription/Injection Order or Administration	YJ Code	2119004F1020	Kalgut tablet 5
Drug 2	22_Prescription/Injection Order or Administration	YJ Code	2119004F1039	Denopameal tablet 5
Drug 2	22_Prescription/Injection Order or Administration	YJ Code	2119004F1047	Herpamine tablet 5
Drug 2	22_Prescription/Injection Order or Administration	YJ Code	2119004F1055	Denopamine tablet 5mg [NICHI-IKO]
Drug 2	22_Prescription/Injection Order or Administration	YJ Code	2119004F1MID	Generic name code
Drug 2	22_Prescription/Injection Order or Administration	YJ Code	2119004F2027	Kalgut tablet 10
Drug 2	22_Prescription/Injection Order or Administration	YJ Code	2119004F2035	Denopameal tablet 10
Drug 2	22_Prescription/Injection Order or Administration	YJ Code	2119004F2043	Herpamine tablet 10
Drug 2	22_Prescription/Injection Order or Administration	YJ Code	2119004F2051	Denopamine tablet 10mg [NICHI-IKO]
Drug 2	22_Prescription/Injection Order or Administration	YJ Code	2119004F2MID	Generic name code
Drug 2	22_Prescription/Injection Order or Administration	YJ Code	2119006F1020	Pimobendan tablet 1.25mg [TE]
Drug 2	22_Prescription/Injection Order or Administration	YJ Code	2119006F1MID	Generic name code
Drug 2	22_Prescription/Injection Order or Administration	YJ Code	2119006F2026	Pimobendan tablet 2.5mg [TE]
Drug 2	22_Prescription/Injection Order or Administration	YJ Code	2119006F2MID	Generic name code
Drug 2	22_Prescription/Injection Order or Administration	YJ Code	2119006F3022	Pimobendan tablet 0.625mg [TE]
Drug 2	22_Prescription/Injection Order or Administration	YJ Code	2119006F3MID	Generic name code
Drug 2	22_Prescription/Injection Order or Administration	YJ Code	2119006M1029	Acardi capsule 1.25
Drug 2	22_Prescription/Injection Order or Administration	YJ Code	2119006M1MID	Generic name code
Drug 2	22_Prescription/Injection Order or Administration	YJ Code	2119006M2025	Acardi capsule 2.5
Drug 2	22_Prescription/Injection Order or Administration	YJ Code	2119006M2MID	Generic name code
Drug 2	22_Prescription/Injection Order or Administration	YJ Code	2119007D1023	Tanadopa granule
Drug 2	22_Prescription/Injection Order or Administration	YJ Code	2119007D1031	Tanadopa granule 75%
Drug 2	22_Prescription/Injection Order or Administration	YJ Code	2119007D1MID	Generic name code
Drug 2	23_DPC or Receipt Pharmaceutical Information	Electronic medical claims processing code	610406380	Acardi capsule 1.25mg
Drug 2	23_DPC or Receipt Pharmaceutical Information	Electronic medical claims processing code	610406381	Acardi capsule 2.5mg
Drug 2	23_DPC or Receipt Pharmaceutical Information	Electronic medical claims processing code	610406396	Tanadopa granule 75%
Drug 2	23_DPC or Receipt Pharmaceutical Information	Electronic medical claims processing code	610453079	Denopameal tablet 5mg
Drug 2	23_DPC or Receipt Pharmaceutical Information	Electronic medical claims processing code	610453080	Denopameal tablet 10mg
Drug 2	23_DPC or Receipt Pharmaceutical Information	Electronic medical claims processing code	610453115	Herpamine tablet 5mg
Drug 2	23_DPC or Receipt Pharmaceutical Information	Electronic medical claims processing code	610453116	Herpamine tablet 10mg
Drug 2	23_DPC or Receipt Pharmaceutical Information	Electronic medical claims processing code	612110004	Adelir tablet 10mg
Drug 2	23_DPC or Receipt Pharmaceutical Information	Electronic medical claims processing code	612110016	Inokiten 10mg
Drug 2	23_DPC or Receipt Pharmaceutical Information	Electronic medical claims processing code	612110018	Inokiten capsule 5mg
Drug 2	23_DPC or Receipt Pharmaceutical Information	Electronic medical claims processing code	612110021	Inokiten granule 1%
Drug 2	23_DPC or Receipt Pharmaceutical Information	Electronic medical claims processing code	612110024	Udekinon tablet 10mg
Drug 2	23_DPC or Receipt Pharmaceutical Information	Electronic medical claims processing code	612110031	Emitolon tablet 10mg
Drug 2	23_DPC or Receipt Pharmaceutical Information	Electronic medical claims processing code	612110055	Carbiquinone tablet 10mg
Drug 2	23_DPC or Receipt Pharmaceutical Information	Electronic medical claims processing code	612110084	Coloquinon tablet 10mg
Drug 2	23_DPC or Receipt Pharmaceutical Information	Electronic medical claims processing code	612110087	Sanquinone tablet 10mg
Drug 2	23_DPC or Receipt Pharmaceutical Information	Electronic medical claims processing code	612110093	Generic ubidecarenone tablet 10mg (discontinued drug)
Drug 2	23_DPC or Receipt Pharmaceutical Information	Electronic medical claims processing code	612110119	Soranekinon tablet 10mg
Drug 2	23_DPC or Receipt Pharmaceutical Information	Electronic medical claims processing code	612110136	Dekasoft 10mg
Drug 2	23_DPC or Receipt Pharmaceutical Information	Electronic medical claims processing code	612110139	Decarenone granule 1%
Drug 2	23_DPC or Receipt Pharmaceutical Information	Electronic medical claims processing code	612110142	Decantoin tablet 10mg
Drug 2	23_DPC or Receipt Pharmaceutical Information	Electronic medical claims processing code	612110154	Neuquinon tablet 10mg
Drug 2	23_DPC or Receipt Pharmaceutical Information	Electronic medical claims processing code	612110155	Neuquinon tablet 5mg
Drug 2	23_DPC or Receipt Pharmaceutical Information	Electronic medical claims processing code	612110156	Neuquinon sugar coating tablet 10mg
Drug 2	23_DPC or Receipt Pharmaceutical Information	Electronic medical claims processing code	612110169	Heartcin tablet 10mg
Drug 2	23_DPC or Receipt Pharmaceutical Information	Electronic medical claims processing code	612110240	Yube-Q 5mg
Drug 2	23_DPC or Receipt Pharmaceutical Information	Electronic medical claims processing code	612110242	Yube-Q 10mg
Drug 2	23_DPC or Receipt Pharmaceutical Information	Electronic medical claims processing code	612110243	Yube-Q granule
Drug 2	23_DPC or Receipt Pharmaceutical Information	Electronic medical claims processing code	612110247	Youbiquinon tablet 10mg
Drug 2	23_DPC or Receipt Pharmaceutical Information	Electronic medical claims processing code	612110248	Lacobile tablet 10mg
Drug 2	23_DPC or Receipt Pharmaceutical Information	Electronic medical claims processing code	612110269	Rasanen tablet 10mg
Drug 2	23_DPC or Receipt Pharmaceutical Information	Electronic medical claims processing code	612110342	Kalgut granule 5%
Drug 2	23_DPC or Receipt Pharmaceutical Information	Electronic medical claims processing code	612110343	Kalgut tablet 5mg
Drug 2	23_DPC or Receipt Pharmaceutical Information	Electronic medical claims processing code	612110344	Kalgut tablet 10mg
Drug 2	23_DPC or Receipt Pharmaceutical Information	Electronic medical claims processing code	620003590	Neuquinon capsule 5mg
Drug 2	23_DPC or Receipt Pharmaceutical Information	Electronic medical claims processing code	620003607	Frupanon tablet 10mg
Drug 2	23_DPC or Receipt Pharmaceutical Information	Electronic medical claims processing code	620003637	Ubequinon capsule 10mg
Drug 2	23_DPC or Receipt Pharmaceutical Information	Electronic medical claims processing code	620004466	Kaitron tablet 10mg
Drug 2	23_DPC or Receipt Pharmaceutical Information	Electronic medical claims processing code	620004535	Tridemin granule 1%
Drug 2	23_DPC or Receipt Pharmaceutical Information	Electronic medical claims processing code	620004536	Tridemin tablet 10mg
Drug 2	23_DPC or Receipt Pharmaceutical Information	Electronic medical claims processing code	620004544	Neuquinon granule 1%
Drug 2	23_DPC or Receipt Pharmaceutical Information	Electronic medical claims processing code	620004930	Inokiten tablet 10mg
Drug 2	23_DPC or Receipt Pharmaceutical Information	Electronic medical claims processing code	620004965	Kalgut granule 5%
Drug 2	23_DPC or Receipt Pharmaceutical Information	Electronic medical claims processing code	620005037	Tanadopa granule 75%
Drug 2	23_DPC or Receipt Pharmaceutical Information	Electronic medical claims processing code	620005918	Inokiten capsule 5mg
Drug 2	23_DPC or Receipt Pharmaceutical Information	Electronic medical claims processing code	620005919	Inokiten granule 1%
Drug 2	23_DPC or Receipt Pharmaceutical Information	Electronic medical claims processing code	620006150	Ubidecarenone tablet 10mg [TOWA]
Drug 2	23_DPC or Receipt Pharmaceutical Information	Electronic medical claims processing code	620006151	Yube-Q capsule 5mg
Drug 2	23_DPC or Receipt Pharmaceutical Information	Electronic medical claims processing code	620006152	Yube-Q granule
Drug 2	23_DPC or Receipt Pharmaceutical Information	Electronic medical claims processing code	620006153	Yube-Q tablet 10mg
Drug 2	23_DPC or Receipt Pharmaceutical Information	Electronic medical claims processing code	620006883	Carbiquinone tablet 10mg
Drug 2	23_DPC or Receipt Pharmaceutical Information	Electronic medical claims processing code	620006984	Dekasoft capsule 10mg
Drug 2	23_DPC or Receipt Pharmaceutical Information	Electronic medical claims processing code	620008499	Pimobendan tablet 1.25mg [TE]
Drug 2	23_DPC or Receipt Pharmaceutical Information	Electronic medical claims processing code	620008500	Pimobendan tablet 2.5mg [TE]
Drug 2	23_DPC or Receipt Pharmaceutical Information	Electronic medical claims processing code	620241604	Ubidecarenone granule 1% [TSURUHARA]
Drug 2	23_DPC or Receipt Pharmaceutical Information	Electronic medical claims processing code	620242301	Lacobile tablet 10mg
Drug 2	23_DPC or Receipt Pharmaceutical Information	Electronic medical claims processing code	620242303	Ubidecarenone tablet 10mg [ISEI]
Drug 2	23_DPC or Receipt Pharmaceutical Information	Electronic medical claims processing code	620242309	Ubidecarenone tablet 10mg [SAWAI]
Drug 2	23_DPC or Receipt Pharmaceutical Information	Electronic medical claims processing code	620242329	Ubidecarenone tablet 10mg [TSURUHARA]
Drug 2	23_DPC or Receipt Pharmaceutical Information	Electronic medical claims processing code	620242343	Ubidecarenone tablet 10mg [NISSIN]
Drug 2	23_DPC or Receipt Pharmaceutical Information	Electronic medical claims processing code	620242409	Ubidecarenone capsule 5mg [TSURUHARA]
Drug 2	23_DPC or Receipt Pharmaceutical Information	Electronic medical claims processing code	620242904	Ubidecarenone capsule 10mg [KYORIN]
Drug 2	23_DPC or Receipt Pharmaceutical Information	Electronic medical claims processing code	621398701	Denopamine tablet 5mg [NICHI-IKO]
Drug 2	23_DPC or Receipt Pharmaceutical Information	Electronic medical claims processing code	621398901	Denopamine tablet 10mg [NICHI-IKO]
Drug 2	23_DPC or Receipt Pharmaceutical Information	Electronic medical claims processing code	622012801	Ubidecarenone capsule 5mg [TOWA]
Drug 2	23_DPC or Receipt Pharmaceutical Information	Electronic medical claims processing code	622018901	Adelir tablet 5mg

Drug 2	23_DPC or Receipt Pharmaceutical Information	Electronic medical claims processing code	622052701	Kaitron tablet 5mg
Drug 2	23_DPC or Receipt Pharmaceutical Information	Electronic medical claims processing code	622052702	Ubidecarenone tablet 5mg [SAWAI]
Drug 2	23_DPC or Receipt Pharmaceutical Information	Electronic medical claims processing code	622064101	Carbiquinone capsule 5mg
Drug 2	23_DPC or Receipt Pharmaceutical Information	Electronic medical claims processing code	622064102	Ubidecarenone capsule 5mg [NISSIN]
Drug 2	23_DPC or Receipt Pharmaceutical Information	Electronic medical claims processing code	622079001	Ubequinon capsule 5mg
Drug 2	23_DPC or Receipt Pharmaceutical Information	Electronic medical claims processing code	622080601	Ubidecarenone capsule 5mg [KYORIN]
Drug 2	23_DPC or Receipt Pharmaceutical Information	Electronic medical claims processing code	622123401	Tridemin capsule 5mg
Drug 2	23_DPC or Receipt Pharmaceutical Information	Electronic medical claims processing code	622288301	Pimobendan tablet 0.625mg [TE]
Drug 2	23_DPC or Receipt Pharmaceutical Information	Electronic medical claims processing code	622324600	Ubidecarenone 1% granule
Drug 2	23_DPC or Receipt Pharmaceutical Information	Electronic medical claims processing code	622324700	Ubidecarenone 10mg tablet
Drug 2	23_DPC or Receipt Pharmaceutical Information	Electronic medical claims processing code	622718100	Ubidecarenone 1% granule
Drug 2	23_DPC or Receipt Pharmaceutical Information	Electronic medical claims processing code	622718200	Ubidecarenone 5mg tablet
Drug 2	23_DPC or Receipt Pharmaceutical Information	Electronic medical claims processing code	622718300	Ubidecarenone 10mg tablet
Drug 2	23_DPC or Receipt Pharmaceutical Information	Electronic medical claims processing code	622718400	Ubidecarenone 5mg capsule
Drug 2	23_DPC or Receipt Pharmaceutical Information	Electronic medical claims processing code	622718500	Ubidecarenone 10mg capsule
Drug 2	23_DPC or Receipt Pharmaceutical Information	Electronic medical claims processing code	622718600	Denopamine 5mg tablet
Drug 2	23_DPC or Receipt Pharmaceutical Information	Electronic medical claims processing code	622718700	Denopamine 10mg tablet
Drug 3	22_Prescription/Injection Order or Administration	YJ Code	2133001C1038	Aldactone-A granule
Drug 3	22_Prescription/Injection Order or Administration	YJ Code	2133001C1070	Merlactone granule
Drug 3	22_Prescription/Injection Order or Administration	YJ Code	2133001C1089	Lacalmin granule
Drug 3	22_Prescription/Injection Order or Administration	YJ Code	2133001C1097	Aldactone-A granule 10%
Drug 3	22_Prescription/Injection Order or Administration	YJ Code	2133001C1100	Merlactone granule 10%
Drug 3	22_Prescription/Injection Order or Administration	YJ Code	2133001C1MID	Generic name code
Drug 3	22_Prescription/Injection Order or Administration	YJ Code	2133001F1050	Aporasnon tablet
Drug 3	22_Prescription/Injection Order or Administration	YJ Code	2133001F1069	Aldactone-A tablet
Drug 3	22_Prescription/Injection Order or Administration	YJ Code	2133001F1085	Almatol tablet
Drug 3	22_Prescription/Injection Order or Administration	YJ Code	2133001F1093	Alexan tablet
Drug 3	22_Prescription/Injection Order or Administration	YJ Code	2133001F1107	Urusonin tablet
Drug 3	22_Prescription/Injection Order or Administration	YJ Code	2133001F1166	Spilactone tablet [SANKEI]
Drug 3	22_Prescription/Injection Order or Administration	YJ Code	2133001F1182	Spironolactone tablet [OHKURA]
Drug 3	22_Prescription/Injection Order or Administration	YJ Code	2133001F1190	Spironolactone tablet [TOWA]
Drug 3	22_Prescription/Injection Order or Administration	YJ Code	2133001F1255	Dira tablet
Drug 3	22_Prescription/Injection Order or Administration	YJ Code	2133001F1280	Noidouble tablet
Drug 3	22_Prescription/Injection Order or Administration	YJ Code	2133001F1301	Bastolebon-A tablet
Drug 3	22_Prescription/Injection Order or Administration	YJ Code	2133001F1328	Pirolacton tablet
Drug 3	22_Prescription/Injection Order or Administration	YJ Code	2133001F1352	Macacy-A tablet
Drug 3	22_Prescription/Injection Order or Administration	YJ Code	2133001F1387	Merlactone tablet
Drug 3	22_Prescription/Injection Order or Administration	YJ Code	2133001F1395	Youlactone tablet 25
Drug 3	22_Prescription/Injection Order or Administration	YJ Code	2133001F1409	Rakudeen
Drug 3	22_Prescription/Injection Order or Administration	YJ Code	2133001F1425	Lacalmin tablet
Drug 3	22_Prescription/Injection Order or Administration	YJ Code	2133001F1441	Generic ubidecarenone tablet 25 (discontinued drug)
Drug 3	22_Prescription/Injection Order or Administration	YJ Code	2133001F1450	Macacy-A tablet 25mg
Drug 3	22_Prescription/Injection Order or Administration	YJ Code	2133001F1468	Alexan tablet 25mg
Drug 3	22_Prescription/Injection Order or Administration	YJ Code	2133001F1476	Spironolactone tablet 25mg [TOWA]
Drug 3	22_Prescription/Injection Order or Administration	YJ Code	2133001F1484	Noidouble tablet 25mg
Drug 3	22_Prescription/Injection Order or Administration	YJ Code	2133001F1492	Pirolacton tablet 25mg
Drug 3	22_Prescription/Injection Order or Administration	YJ Code	2133001F1506	Lacalmin tablet 25mg
Drug 3	22_Prescription/Injection Order or Administration	YJ Code	2133001F1514	Aporasnon tablet 25mg
Drug 3	22_Prescription/Injection Order or Administration	YJ Code	2133001F1522	Aldactone-A tablet 25mg
Drug 3	22_Prescription/Injection Order or Administration	YJ Code	2133001F1530	Merlactone tablet 25mg
Drug 3	22_Prescription/Injection Order or Administration	YJ Code	2133001F1549	Urusonin tablet 25mg
Drug 3	22_Prescription/Injection Order or Administration	YJ Code	2133001F1557	Almatol tablet 25mg
Drug 3	22_Prescription/Injection Order or Administration	YJ Code	2133001F1565	Spilactone tablet 25mg [SANKEI]
Drug 3	22_Prescription/Injection Order or Administration	YJ Code	2133001F1573	Rakudeen tablet 25mg
Drug 3	22_Prescription/Injection Order or Administration	YJ Code	2133001F1581	Spironolactone tablet 25mg [TANABE]
Drug 3	22_Prescription/Injection Order or Administration	YJ Code	2133001F1590	Spironolactone tablet 25mg [NICHI-IKO]
Drug 3	22_Prescription/Injection Order or Administration	YJ Code	2133001F1603	Spironolactone tablet 25mg [YD]
Drug 3	22_Prescription/Injection Order or Administration	YJ Code	2133001F1611	Spironolactone tablet 25mg [TEVA]
Drug 3	22_Prescription/Injection Order or Administration	YJ Code	2133001F1620	Spironolactone tablet 25mg [CH]
Drug 3	22_Prescription/Injection Order or Administration	YJ Code	2133001F1638	Spironolactone tablet 25mg [TSURUHARA]
Drug 3	22_Prescription/Injection Order or Administration	YJ Code	2133001F1646	Spironolactone tablet 25mg [TCK]
Drug 3	22_Prescription/Injection Order or Administration	YJ Code	2133001F1654	Spironolactone tablet 25mg [NP]
Drug 3	22_Prescription/Injection Order or Administration	YJ Code	2133001F1662	Spironolactone tablet 25mg [KYORIN]
Drug 3	22_Prescription/Injection Order or Administration	YJ Code	2133001F1MID	Generic name code
Drug 3	22_Prescription/Injection Order or Administration	YJ Code	2133001F2057	Aldactone-A tablet 50mg
Drug 3	22_Prescription/Injection Order or Administration	YJ Code	2133001F2065	Almatol tablet 50mg
Drug 3	22_Prescription/Injection Order or Administration	YJ Code	2133001F2111	Bastolebon-A tablet 50
Drug 3	22_Prescription/Injection Order or Administration	YJ Code	2133001F2154	Youlactone tablet 50
Drug 3	22_Prescription/Injection Order or Administration	YJ Code	2133001F2162	Lacalmin tablet [50]
Drug 3	22_Prescription/Injection Order or Administration	YJ Code	2133001F2170	Spironolactone tablet 50mg [TANABE]
Drug 3	22_Prescription/Injection Order or Administration	YJ Code	2133001F2189	Spironolactone tablet 50mg [YD]
Drug 3	22_Prescription/Injection Order or Administration	YJ Code	2133001F2197	Spironolactone tablet 50mg [CH]
Drug 3	22_Prescription/Injection Order or Administration	YJ Code	2133001F2MID	Generic name code
Drug 3	22_Prescription/Injection Order or Administration	YJ Code	2133002D1020	Diuteren granule
Drug 3	22_Prescription/Injection Order or Administration	YJ Code	2133002D1MID	Generic name code
Drug 3	22_Prescription/Injection Order or Administration	YJ Code	2133002F1055	Diuteren tablet
Drug 3	22_Prescription/Injection Order or Administration	YJ Code	2133002F1080	Triamterene tablet [ISEI]
Drug 3	22_Prescription/Injection Order or Administration	YJ Code	2133002F1179	Diuteren tablet 30mg
Drug 3	22_Prescription/Injection Order or Administration	YJ Code	2133002F1187	Triamterene tablet 30mg [ISEI]
Drug 3	22_Prescription/Injection Order or Administration	YJ Code	2133002F1MID	Generic name code
Drug 3	22_Prescription/Injection Order or Administration	YJ Code	2133002F2035	Generic triamterene tablet (discontinued drug)
Drug 3	22_Prescription/Injection Order or Administration	YJ Code	2133002F2MID	Generic name code
Drug 3	22_Prescription/Injection Order or Administration	YJ Code	2133002M1054	Triteren capsule
Drug 3	22_Prescription/Injection Order or Administration	YJ Code	2133002M1062	Triteren capsule 50mg
Drug 3	22_Prescription/Injection Order or Administration	YJ Code	2133002M1MID	Generic name code
Drug 3	22_Prescription/Injection Order or Administration	YJ Code	21330A0F1025	Spironolactone tablet 25mg [KYORIN]
Drug 3	22_Prescription/Injection Order or Administration	YJ Code	21330A0F1MID	Generic name code
Drug 3	22_Prescription/Injection Order or Administration	YJ Code	2133400D1023	Soldactone 100mg
Drug 3	22_Prescription/Injection Order or Administration	YJ Code	2133400D1031	Generic canrenoate potassium injection (discontinued drug)
Drug 3	22_Prescription/Injection Order or Administration	YJ Code	2133400D1058	Narmyloninjection 100
Drug 3	22_Prescription/Injection Order or Administration	YJ Code	2133400D1066	Venectomin injection
Drug 3	22_Prescription/Injection Order or Administration	YJ Code	2133400D1074	Venectomin injection 100mg
Drug 3	22_Prescription/Injection Order or Administration	YJ Code	2133400D1082	Soldactoneinjection 100mg
Drug 3	22_Prescription/Injection Order or Administration	YJ Code	2133400D1MID	Generic name code
Drug 3	22_Prescription/Injection Order or Administration	YJ Code	2133400D2020	Gascool injection 200
Drug 3	22_Prescription/Injection Order or Administration	YJ Code	2133400D2038	Soldactone 200mg
Drug 3	22_Prescription/Injection Order or Administration	YJ Code	2133400D2046	Generic canrenoate potassium injection (discontinued drug)
Drug 3	22_Prescription/Injection Order or Administration	YJ Code	2133400D2062	Narmylon injection 200
Drug 3	22_Prescription/Injection Order or Administration	YJ Code	2133400D2070	Venectomin injection
Drug 3	22_Prescription/Injection Order or Administration	YJ Code	2133400D2089	Venectomin injection 200mg
Drug 3	22_Prescription/Injection Order or Administration	YJ Code	2133400D2097	Soldactone injection 200mg
Drug 3	22_Prescription/Injection Order or Administration	YJ Code	2133400D2MID	Generic name code
Drug 3	22_Prescription/Injection Order or Administration	YJ Code	2133400D3026	Gascool injection 200mg
Drug 3	22_Prescription/Injection Order or Administration	YJ Code	2133400D3034	Potassium canrenoate injection 200mg [SAWAI]
Drug 3	22_Prescription/Injection Order or Administration	YJ Code	2133400D3MID	Generic name code
Drug 3	22_Prescription/Injection Order or Administration	YJ Code	2133400D4022	Potassium canrenoate injection 100mg [SAWAI]
Drug 3	22_Prescription/Injection Order or Administration	YJ Code	2133400D4MID	Generic name code
Drug 3	23_DPC or Receipt Pharmaceutical Information	Electronic medical claims processing code	610461153	Spironolactone 10% granule
Drug 3	23_DPC or Receipt Pharmaceutical Information	Electronic medical claims processing code	610461156	Spironolactone 50mg tablet
Drug 3	23_DPC or Receipt Pharmaceutical Information	Electronic medical claims processing code	612130037	Aldactone-A granule 10%
Drug 3	23_DPC or Receipt Pharmaceutical Information	Electronic medical claims processing code	612130038	Aldactone-A tablet 25mg
Drug 3	23_DPC or Receipt Pharmaceutical Information	Electronic medical claims processing code	612130039	Aldactone-A tablet 50mg
Drug 3	23_DPC or Receipt Pharmaceutical Information	Electronic medical claims processing code	612130043	Almatol tablet 25mg
Drug 3	23_DPC or Receipt Pharmaceutical Information	Electronic medical claims processing code	612130054	Urusonin tablet 25mg
Drug 3	23_DPC or Receipt Pharmaceutical Information	Electronic medical claims processing code	612130104	Diuteren tablet 30mg
Drug 3	23_DPC or Receipt Pharmaceutical Information	Electronic medical claims processing code	612130105	Diuteren granule 33.33%
Drug 3	23_DPC or Receipt Pharmaceutical Information	Electronic medical claims processing code	612130110	Spilactone tablet [SANKEI] 25mg
Drug 3	23_DPC or Receipt Pharmaceutical Information	Electronic medical claims processing code	612130156	Triamterene tablet [ISEI] 30mg
Drug 3	23_DPC or Receipt Pharmaceutical Information	Electronic medical claims processing code	612130169	Triteren capsule 50mg
Drug 3	23_DPC or Receipt Pharmaceutical Information	Electronic medical claims processing code	612130260	Merlactone granule 10%
Drug 3	23_DPC or Receipt Pharmaceutical Information	Electronic medical claims processing code	612130261	Merlactone tablet 25mg
Drug 3	23_DPC or Receipt Pharmaceutical Information	Electronic medical claims processing code	612130267	Youlactone tablet 25mg
Drug 3	23_DPC or Receipt Pharmaceutical Information	Electronic medical claims processing code	612130268	Rakudeen 25mg
Drug 3	23_DPC or Receipt Pharmaceutical Information	Electronic medical claims processing code	612130342	Youlactone tablet 50mg
Drug 3	23_DPC or Receipt Pharmaceutical Information	Electronic medical claims processing code	620003083	Macacy-A tablet 25mg
Drug 3	23_DPC or Receipt Pharmaceutical Information	Electronic medical claims processing code	620003510	Alexan tablet 25mg
Drug 3	23_DPC or Receipt Pharmaceutical Information	Electronic medical claims processing code	620003560	Spironolactone tablet 25mg [TOWA]
Drug 3	23_DPC or Receipt Pharmaceutical Information	Electronic medical claims processing code	620003591	Noidouble tablet 25mg
Drug 3	23_DPC or Receipt Pharmaceutical Information	Electronic medical claims processing code	620003807	Venectomin injection 200mg
Drug 3	23_DPC or Receipt Pharmaceutical Information	Electronic medical claims processing code	620004182	Venectomin injection 100mg
Drug 3	23_DPC or Receipt Pharmaceutical Information	Electronic medical claims processing code	620004443	Aporasnon tablet 25mg
Drug 3	23_DPC or Receipt Pharmaceutical Information	Electronic medical claims processing code	620004558	Pirolacton tablet 25mg
Drug 3	23_DPC or Receipt Pharmaceutical Information	Electronic medical claims processing code	620004615	Lacalmin tablet 25mg
Drug 3	23_DPC or Receipt Pharmaceutical Information	Electronic medical claims processing code	620004650	Gascool injection 200mg
Drug 3	23_DPC or Receipt Pharmaceutical Information	Electronic medical claims processing code	620004914	Aldactone-A granule 10%
Drug 3	23_DPC or Receipt Pharmaceutical Information	Electronic medical claims processing code	620004915	Aldactone-A tablet 25mg
Drug 3	23_DPC or Receipt Pharmaceutical Information	Electronic medical claims processing code	620006051	Triteren capsule 50mg
Drug 3	23_DPC or Receipt Pharmaceutical Information	Electronic medical claims processing code	620006149	Merlactone tablet 25mg
Drug 3	23_DPC or Receipt Pharmaceutical Information	Electronic medical claims processing code	620006857	Urusonin tablet 25mg
Drug 3	23_DPC or Receipt Pharmaceutical Information	Electronic medical claims processing code	620008345	Merlactone granule 10%
Drug 3	23_DPC or Receipt Pharmaceutical Information	Electronic medical claims processing code	620008863	Soldactone injection 100mg
Drug 3	23_DPC or Receipt Pharmaceutical Information	Electronic medical claims processing code	620008864	Soldactone injection 200mg
Drug 3	23_DPC or Receipt Pharmaceutical Information	Electronic medical claims processing code	620266102	Spironolactone tablet 25mg [TSURUHARA]
Drug 3	23_DPC or Receipt Pharmaceutical Information	Electronic medical claims processing code	620266110	Spironolactone tablet 25mg [TEVA]
Drug 3	23_DPC or Receipt Pharmaceutical Information	Electronic medical claims processing code	620266112	Spironolactone tablet 25mg [TCK]
Drug 3	23_DPC or Receipt Pharmaceutical Information	Electronic medical claims processing code	620266119	Spironolactone tablet 25mg [NP]
Drug 3	23_DPC or Receipt Pharmaceutical Information	Electronic medical claims processing code	620266125	Rakudeen tablet 25mg
Drug 3	23_DPC or Receipt Pharmaceutical Information	Electronic medical claims processing code	620266131	Spilactone tablet 25mg [SANKEI]
Drug 3	23_DPC or Receipt Pharmaceutical Information	Electronic medical claims processing code	620266132	Spironolactone tablet 25mg [NICHI-IKO]

Drug 3	23_DPC or Receipt Pharmaceutical Information	Electronic medical claims processing code	620266138	Spironolactone tablet 25mg [YD]
Drug 3	23_DPC or Receipt Pharmaceutical Information	Electronic medical claims processing code	620266148	Spironolactone tablet 25mg [KYORIN]
Drug 3	23_DPC or Receipt Pharmaceutical Information	Electronic medical claims processing code	620266301	Almatol tablet 25mg
Drug 3	23_DPC or Receipt Pharmaceutical Information	Electronic medical claims processing code	620266304	Spironolactone tablet 25mg [TANABE]
Drug 3	23_DPC or Receipt Pharmaceutical Information	Electronic medical claims processing code	620266305	Spironolactone tablet 25mg [CH]
Drug 3	23_DPC or Receipt Pharmaceutical Information	Electronic medical claims processing code	620266409	Spironolactone tablet 50mg [YD]
Drug 3	23_DPC or Receipt Pharmaceutical Information	Electronic medical claims processing code	620266412	Spironolactone tablet 50mg [TANABE]
Drug 3	23_DPC or Receipt Pharmaceutical Information	Electronic medical claims processing code	620266413	Spironolactone tablet 50mg [CH]
Drug 3	23_DPC or Receipt Pharmaceutical Information	Electronic medical claims processing code	620266901	Diuteren tablet 30mg
Drug 3	23_DPC or Receipt Pharmaceutical Information	Electronic medical claims processing code	620267001	Triamterene tablet 30mg [ISEI]
Drug 3	23_DPC or Receipt Pharmaceutical Information	Electronic medical claims processing code	620267805	Potassium canrenoate injection 200mg [SAWAI]
Drug 3	23_DPC or Receipt Pharmaceutical Information	Electronic medical claims processing code	622098801	Potassium canrenoate injection 100mg [SAWAI]
Drug 3	23_DPC or Receipt Pharmaceutical Information	Electronic medical claims processing code	622315800	Spironolactone 25mg tablet
Drug 3	23_DPC or Receipt Pharmaceutical Information	Electronic medical claims processing code	622327300	Potassium canrenoate 100mg injection
Drug 3	23_DPC or Receipt Pharmaceutical Information	Electronic medical claims processing code	622327400	Potassium canrenoate 200mg injection
Drug 3	23_DPC or Receipt Pharmaceutical Information	Electronic medical claims processing code	622750700	Potassium canrenoate 200mg injection
Drug 3	23_DPC or Receipt Pharmaceutical Information	Electronic medical claims processing code	622750800	Potassium canrenoate 100mg injection
Drug 3	23_DPC or Receipt Pharmaceutical Information	Electronic medical claims processing code	642130005	Soldactone 100mg
Drug 3	23_DPC or Receipt Pharmaceutical Information	Electronic medical claims processing code	642130006	Soldactone 200mg
Drug 3	23_DPC or Receipt Pharmaceutical Information	Electronic medical claims processing code	642130022	Narmylon injection 100mg
Drug 3	23_DPC or Receipt Pharmaceutical Information	Electronic medical claims processing code	642130026	Narmylon injection 200mg
Drug 4	22_Prescription/Injection Order or Administration	YJ Code	2113001F1023	Digitoxin tablet [SHIONOGI] 0.025mg
Drug 4	22_Prescription/Injection Order or Administration	YJ Code	2113001F1MID	Generic name code
Drug 4	22_Prescription/Injection Order or Administration	YJ Code	2113001F2020	Digitoxin tablet [SHIONOGI] 0.1mg
Drug 4	22_Prescription/Injection Order or Administration	YJ Code	2113001F2038	Digitoxin tablet [FUJISAWA]
Drug 4	22_Prescription/Injection Order or Administration	YJ Code	2113001F2MID	Generic name code
Drug 4	22_Prescription/Injection Order or Administration	YJ Code	2113003F1022	Digoxin [SANDOZ]
Drug 4	22_Prescription/Injection Order or Administration	YJ Code	2113003F1030	Digoxin tablet [YAMANOUCHI]
Drug 4	22_Prescription/Injection Order or Administration	YJ Code	2113003F1049	Digosin tablet
Drug 4	22_Prescription/Injection Order or Administration	YJ Code	2113003F1057	Digosin tablet 0.25mg
Drug 4	22_Prescription/Injection Order or Administration	YJ Code	2113003F1065	Digoxin-KY tablet 0.25
Drug 4	22_Prescription/Injection Order or Administration	YJ Code	2113003F1073	Digoxin tablet 0.25mg [HD]
Drug 4	22_Prescription/Injection Order or Administration	YJ Code	2113003F1081	Digoxin tablet [TAIYO] 0.25mg
Drug 4	22_Prescription/Injection Order or Administration	YJ Code	2113003F1090	Digoxin tablet 0.25mg [AFP]
Drug 4	22_Prescription/Injection Order or Administration	YJ Code	2113003F1103	Digoxin tablet 0.25mg [NP]
Drug 4	22_Prescription/Injection Order or Administration	YJ Code	2113003F1MID	Generic name code
Drug 4	22_Prescription/Injection Order or Administration	YJ Code	2113003F2029	Halfdigoxin-KY tablet 0.125
Drug 4	22_Prescription/Injection Order or Administration	YJ Code	2113003F2037	Digoxin tablet [TAIYO] 0.125mg
Drug 4	22_Prescription/Injection Order or Administration	YJ Code	2113003F2045	Digosin tablet 0.125mg
Drug 4	22_Prescription/Injection Order or Administration	YJ Code	2113003F2053	Digohan tablet 0.125mg
Drug 4	22_Prescription/Injection Order or Administration	YJ Code	2113003F2061	Digoxin tablet 0.125mg [AFP]
Drug 4	22_Prescription/Injection Order or Administration	YJ Code	2113003F2070	Digoxin tablet 0.125mg [NP]
Drug 4	22_Prescription/Injection Order or Administration	YJ Code	2113003F2MID	Generic name code
Drug 4	22_Prescription/Injection Order or Administration	YJ Code	2113003F3025	Digoxin tablet 0.0625 [KYO]
Drug 4	22_Prescription/Injection Order or Administration	YJ Code	2113003F3MID	Generic name code
Drug 4	22_Prescription/Injection Order or Administration	YJ Code	2113004B1025	Digosin 0.1% powder
Drug 4	22_Prescription/Injection Order or Administration	YJ Code	2113004B1033	Digosin powder 0.1%
Drug 4	22_Prescription/Injection Order or Administration	YJ Code	2113004B1MID	Generic name code
Drug 4	22_Prescription/Injection Order or Administration	YJ Code	2113004S1025	Digosin elixir
Drug 4	22_Prescription/Injection Order or Administration	YJ Code	2113004S1033	Digosin elixir 0.05mg/ml
Drug 4	22_Prescription/Injection Order or Administration	YJ Code	2113004S1MID	Generic name code
Drug 4	22_Prescription/Injection Order or Administration	YJ Code	2113005F1021	Lanirapid tablet
Drug 4	22_Prescription/Injection Order or Administration	YJ Code	2113005F1030	Lanirapid tablet 0.1mg
Drug 4	22_Prescription/Injection Order or Administration	YJ Code	2113005F1048	Metildigoxin tablet 0.1mg [TAIYO]
Drug 4	22_Prescription/Injection Order or Administration	YJ Code	2113005F1056	Metildigoxin tablet 0.1mg [NIG]
Drug 4	22_Prescription/Injection Order or Administration	YJ Code	2113005F1MID	Generic name code
Drug 4	22_Prescription/Injection Order or Administration	YJ Code	2113005F2028	Lanirapid tablet 0.05mg
Drug 4	22_Prescription/Injection Order or Administration	YJ Code	2113005F2036	Metildigoxin tablet 0.05mg [TAIYO]
Drug 4	22_Prescription/Injection Order or Administration	YJ Code	2113005F2044	Metildigoxin tablet 0.05mg [NIG]
Drug 4	22_Prescription/Injection Order or Administration	YJ Code	2113005F2MID	Generic name code
Drug 4	22_Prescription/Injection Order or Administration	YJ Code	2113006F1034	Digilanogen-C tablet
Drug 4	22_Prescription/Injection Order or Administration	YJ Code	2113006F1050	Generic lanatoside C tablet (discontinued drug)
Drug 4	22_Prescription/Injection Order or Administration	YJ Code	2113006F1MID	Generic name code
Drug 4	22_Prescription/Injection Order or Administration	YJ Code	211300XF2027	Digoxin tablet 0.0625 [KYO]
Drug 4	22_Prescription/Injection Order or Administration	YJ Code	211300XF2MID	Generic name code
Drug 4	22_Prescription/Injection Order or Administration	YJ Code	2113400A1024	Digosin injection
Drug 4	22_Prescription/Injection Order or Administration	YJ Code	2113400A1032	Digosin injection 0.25mg
Drug 4	22_Prescription/Injection Order or Administration	YJ Code	2113400A1MID	Generic name code
Drug 4	22_Prescription/Injection Order or Administration	YJ Code	2113401A1029	Digilanogen-C injection
Drug 4	22_Prescription/Injection Order or Administration	YJ Code	2113401A1045	Deslanosin injection
Drug 4	22_Prescription/Injection Order or Administration	YJ Code	2113401A1053	Digilanogen injection 0.4mg
Drug 4	22_Prescription/Injection Order or Administration	YJ Code	2113401A1MID	Generic name code
Drug 4	23_DPC or Receipt Pharmaceutical Information	Electronic medical claims processing code	610433147	Lanirapid tablet 0.05mg
Drug 4	23_DPC or Receipt Pharmaceutical Information	Electronic medical claims processing code	610454034	Digosin powder 0.1%
Drug 4	23_DPC or Receipt Pharmaceutical Information	Electronic medical claims processing code	610454089	Lanirapid table 0.1mg
Drug 4	23_DPC or Receipt Pharmaceutical Information	Electronic medical claims processing code	610462017	Digoxin tablet [TAIYO] 0.25mg
Drug 4	23_DPC or Receipt Pharmaceutical Information	Electronic medical claims processing code	610462018	Halfdigoxin-KY tablet 0.125mg
Drug 4	23_DPC or Receipt Pharmaceutical Information	Electronic medical claims processing code	610462019	Digoxin tablet [TAIYO] 0.125mg
Drug 4	23_DPC or Receipt Pharmaceutical Information	Electronic medical claims processing code	610462020	Digosin tablet 0.125mg
Drug 4	23_DPC or Receipt Pharmaceutical Information	Electronic medical claims processing code	610462021	Digohan tablet 0.125mg
Drug 4	23_DPC or Receipt Pharmaceutical Information	Electronic medical claims processing code	610463079	Digoxin tablet 0.125mg
Drug 4	23_DPC or Receipt Pharmaceutical Information	Electronic medical claims processing code	612110098	Digitoxin tablet 0.025mg
Drug 4	23_DPC or Receipt Pharmaceutical Information	Electronic medical claims processing code	612110099	Digitoxin tablet 0.1mg
Drug 4	23_DPC or Receipt Pharmaceutical Information	Electronic medical claims processing code	612110100	Digoxin tablet 0.25mg
Drug 4	23_DPC or Receipt Pharmaceutical Information	Electronic medical claims processing code	612110251	Lanatoside C tablet 0.25mg
Drug 4	23_DPC or Receipt Pharmaceutical Information	Electronic medical claims processing code	612110357	Digitoxin tablet [SHIONOGI] 0.025mg
Drug 4	23_DPC or Receipt Pharmaceutical Information	Electronic medical claims processing code	612110358	Digitoxin tablet [SHIONOGI] 0.1mg
Drug 4	23_DPC or Receipt Pharmaceutical Information	Electronic medical claims processing code	612110363	Digosin tablet 0.25mg
Drug 4	23_DPC or Receipt Pharmaceutical Information	Electronic medical claims processing code	620000519	Digoxin-KY tablet 0.25mg
Drug 4	23_DPC or Receipt Pharmaceutical Information	Electronic medical claims processing code	620000520	Digoxin tablet 0.25mg [HD]
Drug 4	23_DPC or Receipt Pharmaceutical Information	Electronic medical claims processing code	620003064	Digoxin tablet 0.125mg [AFP]
Drug 4	23_DPC or Receipt Pharmaceutical Information	Electronic medical claims processing code	620003065	Digoxin tablet 0.25mg [AFP]
Drug 4	23_DPC or Receipt Pharmaceutical Information	Electronic medical claims processing code	620003550	Digosin elixir 0.05mg/ml 0.005%
Drug 4	23_DPC or Receipt Pharmaceutical Information	Electronic medical claims processing code	620004320	Digosin injection 0.25mg 0.025% 1ml
Drug 4	23_DPC or Receipt Pharmaceutical Information	Electronic medical claims processing code	620005567	Metildigoxin tablet 0.05mg [TAIYO]
Drug 4	23_DPC or Receipt Pharmaceutical Information	Electronic medical claims processing code	620005568	Metildigoxin tablet 0.1mg [TAIYO]
Drug 4	23_DPC or Receipt Pharmaceutical Information	Electronic medical claims processing code	620006616	Digilanogen injection 0.4mg 0.02% 2ml
Drug 4	23_DPC or Receipt Pharmaceutical Information	Electronic medical claims processing code	620236706	Digoxin tablet 0.25mg [NP]
Drug 4	23_DPC or Receipt Pharmaceutical Information	Electronic medical claims processing code	621468501	Digoxin tablet 0.125mg [NP]
Drug 4	23_DPC or Receipt Pharmaceutical Information	Electronic medical claims processing code	621780103	Metildigoxin tablet 0.05mg [NIG]
Drug 4	23_DPC or Receipt Pharmaceutical Information	Electronic medical claims processing code	621780203	Metildigoxin tablet 0.1mg [NIG]
Drug 4	23_DPC or Receipt Pharmaceutical Information	Electronic medical claims processing code	622379401	Digoxin tablet [KYO] 0.0625mg
Drug 4	23_DPC or Receipt Pharmaceutical Information	Electronic medical claims processing code	622717900	Metildigoxin 0.1mg tablet
Drug 4	23_DPC or Receipt Pharmaceutical Information	Electronic medical claims processing code	622718000	Metildigoxin 0.05mg tablet
Drug 4	23_DPC or Receipt Pharmaceutical Information	Electronic medical claims processing code	642110026	Digoxin injection 0.025% 1ml
Drug 4	23_DPC or Receipt Pharmaceutical Information	Electronic medical claims processing code	642110032	Deslanoside injection 0.02% 2ml
Drug 4	23_DPC or Receipt Pharmaceutical Information	Electronic medical claims processing code	642110113	Digilanogen-C injection 0.02% 2ml
Drug 4	23_DPC or Receipt Pharmaceutical Information	Electronic medical claims processing code	642110115	Deslanosin injection 0.02% 2ml
Drug 5	22_Prescription/Injection Order or Administration	YJ Code	2139001Q1020	Menilet 70% jelly 20g
Drug 5	22_Prescription/Injection Order or Administration	YJ Code	2139001Q1039	Isosorbide jelly 70% 20g [NICHI-IKO]
Drug 5	22_Prescription/Injection Order or Administration	YJ Code	2139001Q1MID	Generic name code
Drug 5	22_Prescription/Injection Order or Administration	YJ Code	2139001Q2027	Menilet 70% jelly 30g
Drug 5	22_Prescription/Injection Order or Administration	YJ Code	2139001Q2035	Isosorbide jelly 70% 30g [NICHI-IKO]
Drug 5	22_Prescription/Injection Order or Administration	YJ Code	2139001Q2MID	Generic name code
Drug 5	22_Prescription/Injection Order or Administration	YJ Code	2139001S1028	Isobide
Drug 5	22_Prescription/Injection Order or Administration	YJ Code	2139001S1036	Generic isosorbide oral liquid70% (discontinued drug)
Drug 5	22_Prescription/Injection Order or Administration	YJ Code	2139001S1044	Isosorbide syrup 70% [TAIYO]
Drug 5	22_Prescription/Injection Order or Administration	YJ Code	2139001S1052	Isosorbide oral liquid 70% [ASUKA]
Drug 5	22_Prescription/Injection Order or Administration	YJ Code	2139001S1060	Isobide syrup 70%
Drug 5	22_Prescription/Injection Order or Administration	YJ Code	2139001S1079	Isosorbide oral liquid 70% [CEO]
Drug 5	22_Prescription/Injection Order or Administration	YJ Code	2139001S1MID	Generic name code
Drug 5	22_Prescription/Injection Order or Administration	YJ Code	2139001S2024	Isosorbide oral liquid 70% 30ml [ASUKA]
Drug 5	22_Prescription/Injection Order or Administration	YJ Code	2139001S2032	Isobide syrup 70% 30ml
Drug 5	22_Prescription/Injection Order or Administration	YJ Code	2139001S2040	Isosorbide oral liquid 70% 30ml [CEO]
Drug 5	22_Prescription/Injection Order or Administration	YJ Code	2139001S2MID	Generic name code
Drug 5	22_Prescription/Injection Order or Administration	YJ Code	2139001S3020	Isobide syrup 70% 20ml
Drug 5	22_Prescription/Injection Order or Administration	YJ Code	2139001S3MID	Generic name code
Drug 5	22_Prescription/Injection Order or Administration	YJ Code	2139001S4027	Isobide syrup 70% 23ml
Drug 5	22_Prescription/Injection Order or Administration	YJ Code	2139001S4MID	Generic name code
Drug 5	22_Prescription/Injection Order or Administration	YJ Code	2139001S5023	Isosorbide oral liquid 70% 40ml [CEO]
Drug 5	22_Prescription/Injection Order or Administration	YJ Code	2139001S5MID	Generic name code
Drug 5	22_Prescription/Injection Order or Administration	YJ Code	2139002F1024	Edecril tablet
Drug 5	22_Prescription/Injection Order or Administration	YJ Code	2139002F1MID	Generic name code
Drug 5	22_Prescription/Injection Order or Administration	YJ Code	2139004F1031	Lunetoron tablet
Drug 5	22_Prescription/Injection Order or Administration	YJ Code	2139004F1040	Lunetoron tablet 1mg
Drug 5	22_Prescription/Injection Order or Administration	YJ Code	2139004F1MID	Generic name code
Drug 5	22_Prescription/Injection Order or Administration	YJ Code	2139005C1021	Lasix granule
Drug 5	22_Prescription/Injection Order or Administration	YJ Code	2139005C1030	Radonna granule
Drug 5	22_Prescription/Injection Order or Administration	YJ Code	2139005C1048	Furosemide granule 4% [EMEC]
Drug 5	22_Prescription/Injection Order or Administration	YJ Code	2139005C1056	Generic furosemide granule (discontinued drug)
Drug 5	22_Prescription/Injection Order or Administration	YJ Code	2139005C1064	Lasix granule 4%
Drug 5	22_Prescription/Injection Order or Administration	YJ Code	2139005C1MID	Generic name code
Drug 5	22_Prescription/Injection Order or Administration	YJ Code	2139005F1028	Urex 20mg
Drug 5	22_Prescription/Injection Order or Administration	YJ Code	2139005F1036	Lasix 20mg tablet
Drug 5	22_Prescription/Injection Order or Administration	YJ Code	2139005F1044	Furosemide tablet 20 [TAIYO]
Drug 5	22_Prescription/Injection Order or Administration	YJ Code	2139005F1052	Lasix tablet 20mg
Drug 5	22_Prescription/Injection Order or Administration	YJ Code	2139005F1060	Furosemide tablet 20mg [NP]
Drug 5	22_Prescription/Injection Order or Administration	YJ Code	2139005F1079	Furosemide tablet 20mg [JG]
Drug 5	22_Prescription/Injection Order or Administration	YJ Code	2139005F1087	Furosemide tablet 20mg [TEVA]

Drug 5	22_Prescription/Injection Order or Administration	YJ Code	2139005F1095	Furosemide tablet 20mg [SN]
Drug 5	22_Prescription/Injection Order or Administration	YJ Code	2139005F1109	Furosemide tablet 20mg [TAKEDA TEVA]
Drug 5	22_Prescription/Injection Order or Administration	YJ Code	2139005F1MID	Generic name code
Drug 5	22_Prescription/Injection Order or Administration	YJ Code	2139005F2059	Anfuramide tablet
Drug 5	22_Prescription/Injection Order or Administration	YJ Code	2139005F2067	Urex 40mg
Drug 5	22_Prescription/Injection Order or Administration	YJ Code	2139005F2083	Katlex tablet
Drug 5	22_Prescription/Injection Order or Administration	YJ Code	2139005F2105	Tabilon-A tablet
Drug 5	22_Prescription/Injection Order or Administration	YJ Code	2139005F2130	Foliront
Drug 5	22_Prescription/Injection Order or Administration	YJ Code	2139005F2172	Frusemide tablet [HANKYU]
Drug 5	22_Prescription/Injection Order or Administration	YJ Code	2139005F2202	Furosemide tablet [ISEI]
Drug 5	22_Prescription/Injection Order or Administration	YJ Code	2139005F2245	Furosemide tablet [TOWA]
Drug 5	22_Prescription/Injection Order or Administration	YJ Code	2139005F2253	Furosemide tablet [NAKANO]
Drug 5	22_Prescription/Injection Order or Administration	YJ Code	2139005F2261	Furosemide tablet [MITA]
Drug 5	22_Prescription/Injection Order or Administration	YJ Code	2139005F2270	Promedes tablet
Drug 5	22_Prescription/Injection Order or Administration	YJ Code	2139005F2288	Maoread
Drug 5	22_Prescription/Injection Order or Administration	YJ Code	2139005F2296	Lasix tablet
Drug 5	22_Prescription/Injection Order or Administration	YJ Code	2139005F2318	Radonna tablet
Drug 5	22_Prescription/Injection Order or Administration	YJ Code	2139005F2334	Radiamin tablet
Drug 5	22_Prescription/Injection Order or Administration	YJ Code	2139005F2342	Lasix tablet 40mg
Drug 5	22_Prescription/Injection Order or Administration	YJ Code	2139005F2350	Tabilon tablet 40mg
Drug 5	22_Prescription/Injection Order or Administration	YJ Code	2139005F2369	Furosemide tablet 40 [TAIYO]
Drug 5	22_Prescription/Injection Order or Administration	YJ Code	2139005F2377	Furosemide tablet 40mg [FUSO]
Drug 5	22_Prescription/Injection Order or Administration	YJ Code	2139005F2385	Furosemide tablet 40mg [NP]
Drug 5	22_Prescription/Injection Order or Administration	YJ Code	2139005F2393	Furosemide tablet 40mg [MITA]
Drug 5	22_Prescription/Injection Order or Administration	YJ Code	2139005F2407	Furosemide tablet 40mg [TOWA]
Drug 5	22_Prescription/Injection Order or Administration	YJ Code	2139005F2415	Furosemide tablet 40mg [ISEI]
Drug 5	22_Prescription/Injection Order or Administration	YJ Code	2139005F2423	Maoread tablet 40mg
Drug 5	22_Prescription/Injection Order or Administration	YJ Code	2139005F2431	Furosemide tablet 40mg [JG]
Drug 5	22_Prescription/Injection Order or Administration	YJ Code	2139005F2440	Furosemide tablet 40mg [TEVA]
Drug 5	22_Prescription/Injection Order or Administration	YJ Code	2139005F2458	Furosemide tablet 40mg [SN]
Drug 5	22_Prescription/Injection Order or Administration	YJ Code	2139005F2466	Furosemide tablet 40mg [TAKEDA TEVA]
Drug 5	22_Prescription/Injection Order or Administration	YJ Code	2139005F2MID	Generic name code
Drug 5	22_Prescription/Injection Order or Administration	YJ Code	2139005F3020	Furosemide tablet 10mg [NP]
Drug 5	22_Prescription/Injection Order or Administration	YJ Code	2139005F3039	Lasix tablet 10mg
Drug 5	22_Prescription/Injection Order or Administration	YJ Code	2139005F3047	Furosemide tablet 10mg [SN]
Drug 5	22_Prescription/Injection Order or Administration	YJ Code	2139005F3055	Furosemide tablet 10mg [TAKEDA TEVA]
Drug 5	22_Prescription/Injection Order or Administration	YJ Code	2139005F3MID	Generic name code
Drug 5	22_Prescription/Injection Order or Administration	YJ Code	2139005N1022	Eutensin
Drug 5	22_Prescription/Injection Order or Administration	YJ Code	2139005N1030	Carmack TP capsule
Drug 5	22_Prescription/Injection Order or Administration	YJ Code	2139005N1057	Eutensin capsule 40mg
Drug 5	22_Prescription/Injection Order or Administration	YJ Code	2139005N1MID	Generic name code
Drug 5	22_Prescription/Injection Order or Administration	YJ Code	2139007F1027	Arelix 3mg tablet
Drug 5	22_Prescription/Injection Order or Administration	YJ Code	2139007F1MID	Generic name code
Drug 5	22_Prescription/Injection Order or Administration	YJ Code	2139007F2023	Arelix 6mg tablet
Drug 5	22_Prescription/Injection Order or Administration	YJ Code	2139007F2MID	Generic name code
Drug 5	22_Prescription/Injection Order or Administration	YJ Code	2139008F1021	Diart tablet
Drug 5	22_Prescription/Injection Order or Administration	YJ Code	2139008F1030	Daitalic tablet 60mg
Drug 5	22_Prescription/Injection Order or Administration	YJ Code	2139008F1048	Azoselic tablet 60mg
Drug 5	22_Prescription/Injection Order or Administration	YJ Code	2139008F1056	Diart tablet 60mg
Drug 5	22_Prescription/Injection Order or Administration	YJ Code	2139008F1064	Azosemide tablet 60mg [JG]
Drug 5	22_Prescription/Injection Order or Administration	YJ Code	2139008F1MID	Generic name code
Drug 5	22_Prescription/Injection Order or Administration	YJ Code	2139008F2028	Diart tablet 30mg
Drug 5	22_Prescription/Injection Order or Administration	YJ Code	2139008F2036	Daitalic tablet 30mg
Drug 5	22_Prescription/Injection Order or Administration	YJ Code	2139008F2044	Azosemide tablet 30mg [JG]
Drug 5	22_Prescription/Injection Order or Administration	YJ Code	2139008F2MID	Generic name code
Drug 5	22_Prescription/Injection Order or Administration	YJ Code	2139009F1026	Luprac tablet 4mg
Drug 5	22_Prescription/Injection Order or Administration	YJ Code	2139009F1034	Torasemide tablet 4mg [KO]
Drug 5	22_Prescription/Injection Order or Administration	YJ Code	2139009F1MID	Generic name code
Drug 5	22_Prescription/Injection Order or Administration	YJ Code	2139009F2022	Luprac tablet 8mg
Drug 5	22_Prescription/Injection Order or Administration	YJ Code	2139009F2030	Torasemide tablet 8mg [KO]
Drug 5	22_Prescription/Injection Order or Administration	YJ Code	2139009F2MID	Generic name code
Drug 5	22_Prescription/Injection Order or Administration	YJ Code	2139009F3029	Torasemide OD tablet 4mg [TE]
Drug 5	22_Prescription/Injection Order or Administration	YJ Code	2139009F3MID	Generic name code
Drug 5	22_Prescription/Injection Order or Administration	YJ Code	2139009F4025	Torasemide OD tablet 8mg [TE]
Drug 5	22_Prescription/Injection Order or Administration	YJ Code	2139009F4MID	Generic name code
Drug 5	22_Prescription/Injection Order or Administration	YJ Code	213900YF1021	Lasix tablet 10mg
Drug 5	22_Prescription/Injection Order or Administration	YJ Code	213900YF1MID	Generic name code
Drug 5	22_Prescription/Injection Order or Administration	YJ Code	2139010F1029	Physuline tablet 30mg
Drug 5	22_Prescription/Injection Order or Administration	YJ Code	2139010F1MID	Generic name code
Drug 5	22_Prescription/Injection Order or Administration	YJ Code	2139011D1022	Samsca granule 1%
Drug 5	22_Prescription/Injection Order or Administration	YJ Code	2139011D1MID	Generic name code
Drug 5	22_Prescription/Injection Order or Administration	YJ Code	2139011F1023	Samsca tablet 15mg
Drug 5	22_Prescription/Injection Order or Administration	YJ Code	2139011F1MID	Generic name code
Drug 5	22_Prescription/Injection Order or Administration	YJ Code	2139011F2020	Samsca tablet 7.5mg
Drug 5	22_Prescription/Injection Order or Administration	YJ Code	2139011F2MID	Generic name code
Drug 5	22_Prescription/Injection Order or Administration	YJ Code	2139011F3026	Samsca OD tablet 7.5mg
Drug 5	22_Prescription/Injection Order or Administration	YJ Code	2139011F3MID	Generic name code
Drug 5	22_Prescription/Injection Order or Administration	YJ Code	2139011F4022	Samsca OD tablet 15mg
Drug 5	22_Prescription/Injection Order or Administration	YJ Code	2139011F4MID	Generic name code
Drug 5	22_Prescription/Injection Order or Administration	YJ Code	213901XF2027	Samsca tablet 30mg
Drug 5	22_Prescription/Injection Order or Administration	YJ Code	213901XF2MID	Generic name code
Drug 5	22_Prescription/Injection Order or Administration	YJ Code	21390A0F1029	Azosemide tablet 30mg [JG]
Drug 5	22_Prescription/Injection Order or Administration	YJ Code	21390A0F1037	Azosemide tablet 30mg [DSEP]
Drug 5	22_Prescription/Injection Order or Administration	YJ Code	21390A0F1MID	Generic name code
Drug 5	22_Prescription/Injection Order or Administration	YJ Code	21390A0F2025	Azosemide tablet 60mg [JG]
Drug 5	22_Prescription/Injection Order or Administration	YJ Code	21390A0F2033	Azosemide tablet 60mg [DSEP]
Drug 5	22_Prescription/Injection Order or Administration	YJ Code	21390A0F2MID	Generic name code
Drug 5	22_Prescription/Injection Order or Administration	YJ Code	21390A1F1023	Furosemide tablet 20mg [SN]
Drug 5	22_Prescription/Injection Order or Administration	YJ Code	21390A1F1MID	Generic name code
Drug 5	22_Prescription/Injection Order or Administration	YJ Code	21390A1F2020	Furosemide tablet 40mg [SN]
Drug 5	22_Prescription/Injection Order or Administration	YJ Code	21390A1F2MID	Generic name code
Drug 5	22_Prescription/Injection Order or Administration	YJ Code	21390A1F3034	Furosemide tablet 10mg [TAKEDA TEVA]
Drug 5	22_Prescription/Injection Order or Administration	YJ Code	21390A1F3MID	Generic name code
Drug 5	22_Prescription/Injection Order or Administration	YJ Code	21390A2D1027	Samsca granule 1%
Drug 5	22_Prescription/Injection Order or Administration	YJ Code	21390A2D1MID	Generic name code
Drug 5	22_Prescription/Injection Order or Administration	YJ Code	21390A2F1028	Samsca OD tablet 7.5mg
Drug 5	22_Prescription/Injection Order or Administration	YJ Code	21390A2F1MID	Generic name code
Drug 5	22_Prescription/Injection Order or Administration	YJ Code	21390A2F2024	Samsca OD tablet 15mg
Drug 5	22_Prescription/Injection Order or Administration	YJ Code	21390A2F2MID	Generic name code
Drug 5	22_Prescription/Injection Order or Administration	YJ Code	21390A3F1022	Torasemide tablet 4mg [KO]
Drug 5	22_Prescription/Injection Order or Administration	YJ Code	21390A3F1MID	Generic name code
Drug 5	22_Prescription/Injection Order or Administration	YJ Code	21390A3F2029	Torasemide tablet 8mg [KO]
Drug 5	22_Prescription/Injection Order or Administration	YJ Code	21390A3F2MID	Generic name code
Drug 5	22_Prescription/Injection Order or Administration	YJ Code	21390A3F3025	Torasemide OD table t4mg [TE]
Drug 5	22_Prescription/Injection Order or Administration	YJ Code	21390A3F3MID	Generic name code
Drug 5	22_Prescription/Injection Order or Administration	YJ Code	21390A3F4021	Torasemide OD tablet 8mg [TE]
Drug 5	22_Prescription/Injection Order or Administration	YJ Code	21390A3F4MID	Generic name code
Drug 5	22_Prescription/Injection Order or Administration	YJ Code	2139400A1020	Lunetoron injection
Drug 5	22_Prescription/Injection Order or Administration	YJ Code	2139400A1039	Lunetoron injection 0.5mg
Drug 5	22_Prescription/Injection Order or Administration	YJ Code	2139400A1MID	Generic name code
Drug 5	22_Prescription/Injection Order or Administration	YJ Code	2139401A1025	Lasix 100mg injection
Drug 5	22_Prescription/Injection Order or Administration	YJ Code	2139401A1033	Lasix injection 100mg
Drug 5	22_Prescription/Injection Order or Administration	YJ Code	2139401A1MID	Generic name code
Drug 5	22_Prescription/Injection Order or Administration	YJ Code	2139401A2048	Fuluvamide injection
Drug 5	22_Prescription/Injection Order or Administration	YJ Code	2139401A2056	Furosemide injection [MITA]
Drug 5	22_Prescription/Injection Order or Administration	YJ Code	2139401A2064	Promedes injection
Drug 5	22_Prescription/Injection Order or Administration	YJ Code	2139401A2072	Lasix injection
Drug 5	22_Prescription/Injection Order or Administration	YJ Code	2139401A2099	Radonna injection
Drug 5	22_Prescription/Injection Order or Administration	YJ Code	2139401A2102	Lowpston injection
Drug 5	22_Prescription/Injection Order or Administration	YJ Code	2139401A2110	Anfuramide injection
Drug 5	22_Prescription/Injection Order or Administration	YJ Code	2139401A2129	Floxaid injection 20
Drug 5	22_Prescription/Injection Order or Administration	YJ Code	2139401A2137	Lasix injection 20mg
Drug 5	22_Prescription/Injection Order or Administration	YJ Code	2139401A2145	Furosemide injection [MITA] 20mg
Drug 5	22_Prescription/Injection Order or Administration	YJ Code	2139401A2153	Lowpston injection 20mg
Drug 5	22_Prescription/Injection Order or Administration	YJ Code	2139401A2161	Furosemide injection 20mg [TOWA]
Drug 5	22_Prescription/Injection Order or Administration	YJ Code	2139401A2170	Furosemide injection 20mg [FUSO]
Drug 5	22_Prescription/Injection Order or Administration	YJ Code	2139401A2188	Furosemide injection 20mg [TAIYO]
Drug 5	22_Prescription/Injection Order or Administration	YJ Code	2139401A2196	Furosemide injection 20mg [NICHI-IKO]
Drug 5	22_Prescription/Injection Order or Administration	YJ Code	2139401A2200	Furosemide injection 20mg [TEVA]
Drug 5	22_Prescription/Injection Order or Administration	YJ Code	2139401A2218	Furosemide injection 20mg [SN]
Drug 5	22_Prescription/Injection Order or Administration	YJ Code	2139401A2226	Furosemide injection 20mg [TAKEDA TEVA]
Drug 5	22_Prescription/Injection Order or Administration	YJ Code	2139401A2MID	Generic name code
Drug 5	22_Prescription/Injection Order or Administration	YJ Code	2139401G1028	Furosemide injection 20mg syringe [TAIYO]
Drug 5	22_Prescription/Injection Order or Administration	YJ Code	2139401G1036	Furosemide injection 20mg syringe [TEVA]
Drug 5	22_Prescription/Injection Order or Administration	YJ Code	2139401G1MID	Generic name code
Drug 5	22_Prescription/Injection Order or Administration	YJ Code	2139402A1020	Arelix 6mg injection
Drug 5	22_Prescription/Injection Order or Administration	YJ Code	2139402A1MID	Generic name code
Drug 5	22_Prescription/Injection Order or Administration	YJ Code	21394A0A1024	Furosemide injection 20mg [SN]
Drug 5	22_Prescription/Injection Order or Administration	YJ Code	21394A0A1MID	Generic name code
Drug 5	22_Prescription/Injection Order or Administration	YJ Code	2499012F1022	Samsca tablet 30mg
Drug 5	22_Prescription/Injection Order or Administration	YJ Code	2499012F1MID	Generic name code
Drug 5	22_Prescription/Injection Order or Administration	YJ Code	2499012F2029	Samsca OD tablet 30mg
Drug 5	22_Prescription/Injection Order or Administration	YJ Code	2499012F2MID	Generic name code
Drug 5	22_Prescription/Injection Order or Administration	YJ Code	24990A2F1022	Samsca OD tablet 30mg
Drug 5	22_Prescription/Injection Order or Administration	YJ Code	24990A2F1MID	Generic name code
Drug 5	23_DPC or Receipt Pharmaceutical Information	Electronic medical claims processing code	610431025	Furosemide granule 4% [EMEC]
Drug 5	23_DPC or Receipt Pharmaceutical Information	Electronic medical claims processing code	610432034	Luprac tablet 4mg
Drug 5	23_DPC or Receipt Pharmaceutical Information	Electronic medical claims processing code	610432035	Luprac tablet 8mg
Drug 5	23_DPC or Receipt Pharmaceutical Information	Electronic medical claims processing code	610433078	Daitalic tablet 60mg

Drug 5	23_DPC or Receipt Pharmaceutical Information	Electronic medical claims processing code	610433104	Furosemide tablet [TAIYO] 20mg
Drug 5	23_DPC or Receipt Pharmaceutical Information	Electronic medical claims processing code	610453012	Azoselic tablet 60mg
Drug 5	23_DPC or Receipt Pharmaceutical Information	Electronic medical claims processing code	610454048	Diart tablet 60mg
Drug 5	23_DPC or Receipt Pharmaceutical Information	Electronic medical claims processing code	612130050	Isobide 70%
Drug 5	23_DPC or Receipt Pharmaceutical Information	Electronic medical claims processing code	612130071	Eutensin 40mg
Drug 5	23_DPC or Receipt Pharmaceutical Information	Electronic medical claims processing code	612130202	Foliront 40mg
Drug 5	23_DPC or Receipt Pharmaceutical Information	Electronic medical claims processing code	612130217	Furosemide tablet [ISEI] 40mg
Drug 5	23_DPC or Receipt Pharmaceutical Information	Electronic medical claims processing code	612130220	Furosemide tablet [TOWA] 40mg
Drug 5	23_DPC or Receipt Pharmaceutical Information	Electronic medical claims processing code	612130223	Furosemide tablet [MITA] 40mg
Drug 5	23_DPC or Receipt Pharmaceutical Information	Electronic medical claims processing code	612130249	Maoread 40mg
Drug 5	23_DPC or Receipt Pharmaceutical Information	Electronic medical claims processing code	612130280	Lunetoron tablet 1mg
Drug 5	23_DPC or Receipt Pharmaceutical Information	Electronic medical claims processing code	612130313	Arelix 3mg tablet
Drug 5	23_DPC or Receipt Pharmaceutical Information	Electronic medical claims processing code	612130314	Arelix 6mg tablet
Drug 5	23_DPC or Receipt Pharmaceutical Information	Electronic medical claims processing code	612130353	Diart tablet 30mg
Drug 5	23_DPC or Receipt Pharmaceutical Information	Electronic medical claims processing code	620000167	Lasix tablet 20mg
Drug 5	23_DPC or Receipt Pharmaceutical Information	Electronic medical claims processing code	620000168	Lasix tablet 40mg
Drug 5	23_DPC or Receipt Pharmaceutical Information	Electronic medical claims processing code	620000339	Lasix injection 20mg
Drug 5	23_DPC or Receipt Pharmaceutical Information	Electronic medical claims processing code	620000340	Lasix injection 100mg
Drug 5	23_DPC or Receipt Pharmaceutical Information	Electronic medical claims processing code	620002860	Menilet 70% jelly 20g
Drug 5	23_DPC or Receipt Pharmaceutical Information	Electronic medical claims processing code	620002861	Menilet 70% jelly 30g
Drug 5	23_DPC or Receipt Pharmaceutical Information	Electronic medical claims processing code	620003182	Lasix granule 4%
Drug 5	23_DPC or Receipt Pharmaceutical Information	Electronic medical claims processing code	620003238	Furosemide injection [MITA] 20mg
Drug 5	23_DPC or Receipt Pharmaceutical Information	Electronic medical claims processing code	620003439	Tabilon tablet 40mg
Drug 5	23_DPC or Receipt Pharmaceutical Information	Electronic medical claims processing code	620003440	Furosemide tablet [TAIYO] 40mg
Drug 5	23_DPC or Receipt Pharmaceutical Information	Electronic medical claims processing code	620003612	Furosemide tablet 40mg [FUSO]
Drug 5	23_DPC or Receipt Pharmaceutical Information	Electronic medical claims processing code	620003836	Lowpston injection 20mg
Drug 5	23_DPC or Receipt Pharmaceutical Information	Electronic medical claims processing code	620003889	Isobide dry syrup 7g
Drug 5	23_DPC or Receipt Pharmaceutical Information	Electronic medical claims processing code	620004041	Furosemide tablet 10mg [NP]
Drug 5	23_DPC or Receipt Pharmaceutical Information	Electronic medical claims processing code	620004042	Furosemide tablet 20mg [NP]
Drug 5	23_DPC or Receipt Pharmaceutical Information	Electronic medical claims processing code	620004043	Furosemide tablet 40mg [NP]
Drug 5	23_DPC or Receipt Pharmaceutical Information	Electronic medical claims processing code	620004356	Physuline tablet 30mg
Drug 5	23_DPC or Receipt Pharmaceutical Information	Electronic medical claims processing code	620004758	Furosemide injection 20mg [TOWA]
Drug 5	23_DPC or Receipt Pharmaceutical Information	Electronic medical claims processing code	620004759	Furosemide injection 20mg [FUSO]
Drug 5	23_DPC or Receipt Pharmaceutical Information	Electronic medical claims processing code	620005097	Furosemide tablet 40mg [MITA]
Drug 5	23_DPC or Receipt Pharmaceutical Information	Electronic medical claims processing code	620005221	Furosemide injection 20mg syringe [TAIYO] 2ml
Drug 5	23_DPC or Receipt Pharmaceutical Information	Electronic medical claims processing code	620005362	Isosorbide syrup 70% [TAIYO]
Drug 5	23_DPC or Receipt Pharmaceutical Information	Electronic medical claims processing code	620005550	Furosemide tablet 20mg [JG]
Drug 5	23_DPC or Receipt Pharmaceutical Information	Electronic medical claims processing code	620006110	Furosemide tablet 40mg [TOWA]
Drug 5	23_DPC or Receipt Pharmaceutical Information	Electronic medical claims processing code	620007070	Furosemide tablet 40mg [ISEI]
Drug 5	23_DPC or Receipt Pharmaceutical Information	Electronic medical claims processing code	620007103	Maoread tablet 40mg
Drug 5	23_DPC or Receipt Pharmaceutical Information	Electronic medical claims processing code	620007146	Lunetoron tablet 1mg
Drug 5	23_DPC or Receipt Pharmaceutical Information	Electronic medical claims processing code	620007548	Lunetoron injection 0.5mg
Drug 5	23_DPC or Receipt Pharmaceutical Information	Electronic medical claims processing code	620007899	Isosorbideoral liquid 70% [ASUKA]
Drug 5	23_DPC or Receipt Pharmaceutical Information	Electronic medical claims processing code	620007900	Isosorbideoral liquid 70% 30ml [ASUKA]
Drug 5	23_DPC or Receipt Pharmaceutical Information	Electronic medical claims processing code	620009199	Furosemide injection 20mg [TAIYO]
Drug 5	23_DPC or Receipt Pharmaceutical Information	Electronic medical claims processing code	620269701	Furosemide tablet 20mg [TEVA]
Drug 5	23_DPC or Receipt Pharmaceutical Information	Electronic medical claims processing code	620269703	Furosemide tablet 20mg [TAKEDA TEVA]
Drug 5	23_DPC or Receipt Pharmaceutical Information	Electronic medical claims processing code	620269809	Furosemide tablet 40mg [TEVA]
Drug 5	23_DPC or Receipt Pharmaceutical Information	Electronic medical claims processing code	620269833	Furosemide tablet 40mg [TAKEDA TEVA]
Drug 5	23_DPC or Receipt Pharmaceutical Information	Electronic medical claims processing code	620270101	Eutensin capsule 40mg
Drug 5	23_DPC or Receipt Pharmaceutical Information	Electronic medical claims processing code	620270703	Azosemide tablet 60mg [JG]
Drug 5	23_DPC or Receipt Pharmaceutical Information	Electronic medical claims processing code	620271902	Furosemide injection 20mg [NICHI-IKO]
Drug 5	23_DPC or Receipt Pharmaceutical Information	Electronic medical claims processing code	620272101	Furosemide injection 20mg [TEVA]
Drug 5	23_DPC or Receipt Pharmaceutical Information	Electronic medical claims processing code	620272102	Furosemide injection 20mg [TAKEDA TEVA]
Drug 5	23_DPC or Receipt Pharmaceutical Information	Electronic medical claims processing code	621692703	Isosorbide jelly 70% 20g [NICHI-IKO]
Drug 5	23_DPC or Receipt Pharmaceutical Information	Electronic medical claims processing code	621692803	Isosorbide jelly 70% 30g [NICHI-IKO]
Drug 5	23_DPC or Receipt Pharmaceutical Information	Electronic medical claims processing code	621782401	Furosemide injection 20mg syringe [TEVA] 2ml
Drug 5	23_DPC or Receipt Pharmaceutical Information	Electronic medical claims processing code	621859002	Isosorbide oral liquid 70% [CEO]
Drug 5	23_DPC or Receipt Pharmaceutical Information	Electronic medical claims processing code	621859102	Isosorbide oral liquid 70% 30ml [CEO]
Drug 5	23_DPC or Receipt Pharmaceutical Information	Electronic medical claims processing code	622007201	Samsca tablet 15mg
Drug 5	23_DPC or Receipt Pharmaceutical Information	Electronic medical claims processing code	622065501	Isobide syrup 70%
Drug 5	23_DPC or Receipt Pharmaceutical Information	Electronic medical claims processing code	622065601	Isobide syrup 70% 20ml
Drug 5	23_DPC or Receipt Pharmaceutical Information	Electronic medical claims processing code	622065701	Isobide syrup 70% 23ml
Drug 5	23_DPC or Receipt Pharmaceutical Information	Electronic medical claims processing code	622065801	Isobide syrup 70% 30ml
Drug 5	23_DPC or Receipt Pharmaceutical Information	Electronic medical claims processing code	622077601	Lasix tablet 10mg
Drug 5	23_DPC or Receipt Pharmaceutical Information	Electronic medical claims processing code	622109301	Furosemide tablet 40mg [JG]
Drug 5	23_DPC or Receipt Pharmaceutical Information	Electronic medical claims processing code	622161501	Daitalic tablet 30mg
Drug 5	23_DPC or Receipt Pharmaceutical Information	Electronic medical claims processing code	622161502	Azosemide tablet 30mg [JG]
Drug 5	23_DPC or Receipt Pharmaceutical Information	Electronic medical claims processing code	622174301	Samsca tablet 7.5mg
Drug 5	23_DPC or Receipt Pharmaceutical Information	Electronic medical claims processing code	622184801	Isosorbide oral liquid 70% 40ml [CEO]
Drug 5	23_DPC or Receipt Pharmaceutical Information	Electronic medical claims processing code	622281901	Samsca tablet 30mg
Drug 5	23_DPC or Receipt Pharmaceutical Information	Electronic medical claims processing code	622531501	Furosemide injection 20mg [SN]
Drug 5	23_DPC or Receipt Pharmaceutical Information	Electronic medical claims processing code	622531601	Furosemide tablet 20mg [SN]
Drug 5	23_DPC or Receipt Pharmaceutical Information	Electronic medical claims processing code	622531701	Furosemide tablet 40mg [SN]
Drug 5	23_DPC or Receipt Pharmaceutical Information	Electronic medical claims processing code	622537701	Samsca granule 1%
Drug 5	23_DPC or Receipt Pharmaceutical Information	Electronic medical claims processing code	622548901	Furosemide tablet 10mg [TAKEDA TEVA]
Drug 5	23_DPC or Receipt Pharmaceutical Information	Electronic medical claims processing code	622567401	Furosemide tablet 10mg [SN]
Drug 5	23_DPC or Receipt Pharmaceutical Information	Electronic medical claims processing code	622601001	Torasemide tablet 4mg [KO]
Drug 5	23_DPC or Receipt Pharmaceutical Information	Electronic medical claims processing code	622601101	Torasemide tablet 8mg [KO]
Drug 5	23_DPC or Receipt Pharmaceutical Information	Electronic medical claims processing code	622609402	Torasemide OD tablet 4mg [TE]
Drug 5	23_DPC or Receipt Pharmaceutical Information	Electronic medical claims processing code	622609502	Torasemide OD tablet 8mg [TE]
Drug 5	23_DPC or Receipt Pharmaceutical Information	Electronic medical claims processing code	622694301	Samsca OD tablet 7.5mg
Drug 5	23_DPC or Receipt Pharmaceutical Information	Electronic medical claims processing code	622694401	Samsca OD tablet 15mg
Drug 5	23_DPC or Receipt Pharmaceutical Information	Electronic medical claims processing code	622694501	Samsca OD tablet 30mg
Drug 5	23_DPC or Receipt Pharmaceutical Information	Electronic medical claims processing code	622720900	Isosorbide 70% 20g jelly
Drug 5	23_DPC or Receipt Pharmaceutical Information	Electronic medical claims processing code	622721000	Isosorbide 70% 30g jelly
Drug 5	23_DPC or Receipt Pharmaceutical Information	Electronic medical claims processing code	622721100	Isosorbide 70% liquid
Drug 5	23_DPC or Receipt Pharmaceutical Information	Electronic medical claims processing code	622721200	Isosorbide 70% 40ml liquid
Drug 5	23_DPC or Receipt Pharmaceutical Information	Electronic medical claims processing code	622721300	Azosemide 60mg tablet
Drug 5	23_DPC or Receipt Pharmaceutical Information	Electronic medical claims processing code	622721400	Azosemide 30mg tablet
Drug 5	23_DPC or Receipt Pharmaceutical Information	Electronic medical claims processing code	640412004	Anfuramide injection 20mg
Drug 5	23_DPC or Receipt Pharmaceutical Information	Electronic medical claims processing code	640433068	Floxaid injection 20mg
Drug 5	23_DPC or Receipt Pharmaceutical Information	Electronic medical claims processing code	642130015	Lunetoron injection 0.5mg
Drug 5	23_DPC or Receipt Pharmaceutical Information	Electronic medical claims processing code	642130019	Arelix 6mg injection
Drug 6	22_Prescription/Injection Order or Administration	YJ Code	2139004F1031	Lunetoron tablet
Drug 6	22_Prescription/Injection Order or Administration	YJ Code	2139004F1040	Lunetoron tablet 1mg
Drug 6	22_Prescription/Injection Order or Administration	YJ Code	2139004F1MID	Generic name code
Drug 6	22_Prescription/Injection Order or Administration	YJ Code	2139005C1021	Lasix granule
Drug 6	22_Prescription/Injection Order or Administration	YJ Code	2139005C1030	Radonna granule
Drug 6	22_Prescription/Injection Order or Administration	YJ Code	2139005C1048	Furosemide granule 4% [EMEC]
Drug 6	22_Prescription/Injection Order or Administration	YJ Code	2139005C1056	Generic furosemide granule (discontinued drug)
Drug 6	22_Prescription/Injection Order or Administration	YJ Code	2139005C1064	Lasix granule 4%
Drug 6	22_Prescription/Injection Order or Administration	YJ Code	2139005C1MID	Generic name code
Drug 6	22_Prescription/Injection Order or Administration	YJ Code	2139005F1028	Urex 20mg
Drug 6	22_Prescription/Injection Order or Administration	YJ Code	2139005F1036	Lasix20mg tablet
Drug 6	22_Prescription/Injection Order or Administration	YJ Code	2139005F1044	Furosemide tablet 20 [TAIYO]
Drug 6	22_Prescription/Injection Order or Administration	YJ Code	2139005F1052	Lasix tablet 20mg
Drug 6	22_Prescription/Injection Order or Administration	YJ Code	2139005F1060	Furosemide tablet 20mg [NP]
Drug 6	22_Prescription/Injection Order or Administration	YJ Code	2139005F1079	Furosemide tablet 20mg [JG]
Drug 6	22_Prescription/Injection Order or Administration	YJ Code	2139005F1087	Furosemide tablet 20mg [TEVA]
Drug 6	22_Prescription/Injection Order or Administration	YJ Code	2139005F1095	Furosemide tablet 20mg [SN]
Drug 6	22_Prescription/Injection Order or Administration	YJ Code	2139005F1109	Furosemide tablet 20mg [TAKEDA TEVA]
Drug 6	22_Prescription/Injection Order or Administration	YJ Code	2139005F1MID	Generic name code
Drug 6	22_Prescription/Injection Order or Administration	YJ Code	2139005F2059	Anfuramide tablet
Drug 6	22_Prescription/Injection Order or Administration	YJ Code	2139005F2067	Urex 40mg
Drug 6	22_Prescription/Injection Order or Administration	YJ Code	2139005F2083	Katlex tablet
Drug 6	22_Prescription/Injection Order or Administration	YJ Code	2139005F2105	Tabilon-A tablet
Drug 6	22_Prescription/Injection Order or Administration	YJ Code	2139005F2130	Foliront
Drug 6	22_Prescription/Injection Order or Administration	YJ Code	2139005F2172	Frusemide tablet [HANKYU]
Drug 6	22_Prescription/Injection Order or Administration	YJ Code	2139005F2202	Furosemide tablet [ISEI]
Drug 6	22_Prescription/Injection Order or Administration	YJ Code	2139005F2245	Furosemide tablet [TOWA]
Drug 6	22_Prescription/Injection Order or Administration	YJ Code	2139005F2253	Furosemide tablet [NAKANO]
Drug 6	22_Prescription/Injection Order or Administration	YJ Code	2139005F2261	Furosemide tablet [MITA]
Drug 6	22_Prescription/Injection Order or Administration	YJ Code	2139005F2270	Promedes tablet
Drug 6	22_Prescription/Injection Order or Administration	YJ Code	2139005F2288	Maoread
Drug 6	22_Prescription/Injection Order or Administration	YJ Code	2139005F2296	Lasix tablet
Drug 6	22_Prescription/Injection Order or Administration	YJ Code	2139005F2318	Radonna tablet
Drug 6	22_Prescription/Injection Order or Administration	YJ Code	2139005F2334	Radiamin tablet
Drug 6	22_Prescription/Injection Order or Administration	YJ Code	2139005F2342	Lasix tablet 40mg
Drug 6	22_Prescription/Injection Order or Administration	YJ Code	2139005F2350	Tabilon tablet 40mg
Drug 6	22_Prescription/Injection Order or Administration	YJ Code	2139005F2369	Furosemide tablet 40 [TAIYO]
Drug 6	22_Prescription/Injection Order or Administration	YJ Code	2139005F2377	Furosemide tablet 40mg [FUSO]
Drug 6	22_Prescription/Injection Order or Administration	YJ Code	2139005F2385	Furosemide tablet 40mg [NP]
Drug 6	22_Prescription/Injection Order or Administration	YJ Code	2139005F2393	Furosemide tablet 40mg [MITA]
Drug 6	22_Prescription/Injection Order or Administration	YJ Code	2139005F2407	Furosemide tablet 40mg [TOWA]
Drug 6	22_Prescription/Injection Order or Administration	YJ Code	2139005F2415	Furosemide tablet 40mg [ISEI]
Drug 6	22_Prescription/Injection Order or Administration	YJ Code	2139005F2423	Maoread tablet 40mg
Drug 6	22_Prescription/Injection Order or Administration	YJ Code	2139005F2431	Furosemide tablet 40mg [JG]
Drug 6	22_Prescription/Injection Order or Administration	YJ Code	2139005F2440	Furosemide tablet 40mg [TEVA]
Drug 6	22_Prescription/Injection Order or Administration	YJ Code	2139005F2458	Furosemide tablet 40mg [SN]
Drug 6	22_Prescription/Injection Order or Administration	YJ Code	2139005F2466	Furosemide tablet 40mg [TAKEDA TEVA]
Drug 6	22_Prescription/Injection Order or Administration	YJ Code	2139005F2MID	Generic name code
Drug 6	22_Prescription/Injection Order or Administration	YJ Code	2139005F3020	Furosemide tablet 10mg [NP]
Drug 6	22_Prescription/Injection Order or Administration	YJ Code	2139005F3039	Lasix tablet 10mg
Drug 6	22_Prescription/Injection Order or Administration	YJ Code	2139005F3047	Furosemide tablet 10mg [SN]
Drug 6	22_Prescription/Injection Order or Administration	YJ Code	2139005F3055	Furosemide tablet 10mg [TAKEDA TEVA]
Drug 6	22_Prescription/Injection Order or Administration	YJ Code	2139005F3MID	Generic name code
Drug 6	22_Prescription/Injection Order or Administration	YJ Code	2139005N1022	Eutensin
Drug 6	22_Prescription/Injection Order or Administration	YJ Code	2139005N1030	Carmack TP capsule

Drug 6	22_Prescription/Injection Order or Administration	YJ Code	2139005N1057	Eutensin capsule 40mg
Drug 6	22_Prescription/Injection Order or Administration	YJ Code	2139005N1MID	Generic name code
Drug 6	22_Prescription/Injection Order or Administration	YJ Code	2139007F1027	Arelix 3mg tablet
Drug 6	22_Prescription/Injection Order or Administration	YJ Code	2139007F1MID	Generic name code
Drug 6	22_Prescription/Injection Order or Administration	YJ Code	2139007F2023	Arelix 6mg tablet
Drug 6	22_Prescription/Injection Order or Administration	YJ Code	2139007F2MID	Generic name code
Drug 6	22_Prescription/Injection Order or Administration	YJ Code	2139008F1021	Diart tablet
Drug 6	22_Prescription/Injection Order or Administration	YJ Code	2139008F1030	Daitalic tablet 60mg
Drug 6	22_Prescription/Injection Order or Administration	YJ Code	2139008F1048	Azoselic tablet 60mg
Drug 6	22_Prescription/Injection Order or Administration	YJ Code	2139008F1056	Diart tablet 60mg
Drug 6	22_Prescription/Injection Order or Administration	YJ Code	2139008F1064	Azosemide tablet 60mg [JG]
Drug 6	22_Prescription/Injection Order or Administration	YJ Code	2139008F1MID	Generic name code
Drug 6	22_Prescription/Injection Order or Administration	YJ Code	2139008F2028	Diart tablet 30mg
Drug 6	22_Prescription/Injection Order or Administration	YJ Code	2139008F2036	Daitalic tablet 30mg
Drug 6	22_Prescription/Injection Order or Administration	YJ Code	2139008F2044	Azosemide tablet 30mg [JG]
Drug 6	22_Prescription/Injection Order or Administration	YJ Code	2139008F2MID	Generic name code
Drug 6	22_Prescription/Injection Order or Administration	YJ Code	2139009F1026	Luprac tablet 4mg
Drug 6	22_Prescription/Injection Order or Administration	YJ Code	2139009F1034	Torasemide tablet 4mg [KO]
Drug 6	22_Prescription/Injection Order or Administration	YJ Code	2139009F1MID	Generic name code
Drug 6	22_Prescription/Injection Order or Administration	YJ Code	2139009F2022	Luprac tablet 8mg
Drug 6	22_Prescription/Injection Order or Administration	YJ Code	2139009F2030	Torasemide tablet 8mg [KO]
Drug 6	22_Prescription/Injection Order or Administration	YJ Code	2139009F2MID	Generic name code
Drug 6	22_Prescription/Injection Order or Administration	YJ Code	2139009F3029	Torasemide OD tablet 4mg [TE]
Drug 6	22_Prescription/Injection Order or Administration	YJ Code	2139009F3MID	Generic name code
Drug 6	22_Prescription/Injection Order or Administration	YJ Code	2139009F4025	Torasemide OD tablet 8mg [TE]
Drug 6	22_Prescription/Injection Order or Administration	YJ Code	2139009F4MID	Generic name code
Drug 6	22_Prescription/Injection Order or Administration	YJ Code	213900YF1021	Lasix tablet 10mg
Drug 6	22_Prescription/Injection Order or Administration	YJ Code	213900YF1MID	Generic name code
Drug 6	22_Prescription/Injection Order or Administration	YJ Code	21390A0F1029	Azosemide tablet 30mg [JG]
Drug 6	22_Prescription/Injection Order or Administration	YJ Code	21390A0F1037	Azosemide tablet 30mg [DSEP]
Drug 6	22_Prescription/Injection Order or Administration	YJ Code	21390A0F1MID	Generic name code
Drug 6	22_Prescription/Injection Order or Administration	YJ Code	21390A0F2025	Azosemide tablet 60mg [JG]
Drug 6	22_Prescription/Injection Order or Administration	YJ Code	21390A0F2033	Azosemide tablet 60mg [DSEP]
Drug 6	22_Prescription/Injection Order or Administration	YJ Code	21390A0F2MID	Generic name code
Drug 6	22_Prescription/Injection Order or Administration	YJ Code	21390A1F1023	Furosemide tablet 20mg [SN]
Drug 6	22_Prescription/Injection Order or Administration	YJ Code	21390A1F1MID	Generic name code
Drug 6	22_Prescription/Injection Order or Administration	YJ Code	21390A1F2020	Furosemide tablet 40mg [SN]
Drug 6	22_Prescription/Injection Order or Administration	YJ Code	21390A1F2MID	Generic name code
Drug 6	22_Prescription/Injection Order or Administration	YJ Code	21390A1F3034	Furosemide tablet 10mg [TAKEDA TEVA]
Drug 6	22_Prescription/Injection Order or Administration	YJ Code	21390A1F3MID	Generic name code
Drug 6	22_Prescription/Injection Order or Administration	YJ Code	21390A3F1022	Torasemide tablet 4mg [KO]
Drug 6	22_Prescription/Injection Order or Administration	YJ Code	21390A3F1MID	Generic name code
Drug 6	22_Prescription/Injection Order or Administration	YJ Code	21390A3F2029	Torasemide tablet 8mg [KO]
Drug 6	22_Prescription/Injection Order or Administration	YJ Code	21390A3F2MID	Generic name code
Drug 6	22_Prescription/Injection Order or Administration	YJ Code	21390A3F3025	Torasemide OD tablet 4mg [TE]
Drug 6	22_Prescription/Injection Order or Administration	YJ Code	21390A3F3MID	Generic name code
Drug 6	22_Prescription/Injection Order or Administration	YJ Code	21390A3F4021	Torasemide OD tablet 8mg [TE]
Drug 6	22_Prescription/Injection Order or Administration	YJ Code	21390A3F4MID	Generic name code
Drug 6	22_Prescription/Injection Order or Administration	YJ Code	2139400A1020	Lunetoron injection
Drug 6	22_Prescription/Injection Order or Administration	YJ Code	2139400A1039	Lunetoron injection 0.5mg
Drug 6	22_Prescription/Injection Order or Administration	YJ Code	2139400A1MID	Generic name code
Drug 6	22_Prescription/Injection Order or Administration	YJ Code	2139401A1025	Lasix 100mg injection
Drug 6	22_Prescription/Injection Order or Administration	YJ Code	2139401A1033	Lasix injection 100mg
Drug 6	22_Prescription/Injection Order or Administration	YJ Code	2139401A1MID	Generic name code
Drug 6	22_Prescription/Injection Order or Administration	YJ Code	2139401A2048	Fuluvamide injection
Drug 6	22_Prescription/Injection Order or Administration	YJ Code	2139401A2056	Furosemide injection [MITA]
Drug 6	22_Prescription/Injection Order or Administration	YJ Code	2139401A2064	Promedes injection
Drug 6	22_Prescription/Injection Order or Administration	YJ Code	2139401A2072	Lasix injection
Drug 6	22_Prescription/Injection Order or Administration	YJ Code	2139401A2099	Radonna injection
Drug 6	22_Prescription/Injection Order or Administration	YJ Code	2139401A2102	Lowpston injection
Drug 6	22_Prescription/Injection Order or Administration	YJ Code	2139401A2110	Anfuramide injection
Drug 6	22_Prescription/Injection Order or Administration	YJ Code	2139401A2129	Floxaid injection 20
Drug 6	22_Prescription/Injection Order or Administration	YJ Code	2139401A2137	Lasix injection 20mg
Drug 6	22_Prescription/Injection Order or Administration	YJ Code	2139401A2145	Furosemide injection [MITA] 20mg
Drug 6	22_Prescription/Injection Order or Administration	YJ Code	2139401A2153	Lowpston injection 20mg
Drug 6	22_Prescription/Injection Order or Administration	YJ Code	2139401A2161	Furosemide injection 20mg [TOWA]
Drug 6	22_Prescription/Injection Order or Administration	YJ Code	2139401A2170	Furosemide injection 20mg [FUSO]
Drug 6	22_Prescription/Injection Order or Administration	YJ Code	2139401A2188	Furosemide injection 20mg [TAIYO]
Drug 6	22_Prescription/Injection Order or Administration	YJ Code	2139401A2196	Furosemide injection 20mg [NICHI-IKO]
Drug 6	22_Prescription/Injection Order or Administration	YJ Code	2139401A2200	Furosemide injection 20mg [TEVA]
Drug 6	22_Prescription/Injection Order or Administration	YJ Code	2139401A2218	Furosemide injection 20mg [SN]
Drug 6	22_Prescription/Injection Order or Administration	YJ Code	2139401A2226	Furosemide injection 20mg [TAKEDA TEVA]
Drug 6	22_Prescription/Injection Order or Administration	YJ Code	2139401A2MID	Generic name code
Drug 6	22_Prescription/Injection Order or Administration	YJ Code	2139401G1028	Furosemide injection 20mg syringe [TAIYO]
Drug 6	22_Prescription/Injection Order or Administration	YJ Code	2139401G1036	Furosemide injection 20mg syringe [TEVA]
Drug 6	22_Prescription/Injection Order or Administration	YJ Code	2139401G1MID	Generic name code
Drug 6	22_Prescription/Injection Order or Administration	YJ Code	2139402A1020	Arelix 6mg injection
Drug 6	22_Prescription/Injection Order or Administration	YJ Code	2139402A1MID	Generic name code
Drug 6	22_Prescription/Injection Order or Administration	YJ Code	21394A0A1024	Furosemide injection 20mg [SN]
Drug 6	22_Prescription/Injection Order or Administration	YJ Code	21394A0A1MID	Generic name code
Drug 6	23_DPC or Receipt Pharmaceutical Information	Electronic medical claims processing code	610431025	Furosemide granule 4% [EMEC]
Drug 6	23_DPC or Receipt Pharmaceutical Information	Electronic medical claims processing code	610432034	Luprac tablet 4mg
Drug 6	23_DPC or Receipt Pharmaceutical Information	Electronic medical claims processing code	610432035	Luprac tablet 8mg
Drug 6	23_DPC or Receipt Pharmaceutical Information	Electronic medical claims processing code	610433078	Daitalic tablet 60mg
Drug 6	23_DPC or Receipt Pharmaceutical Information	Electronic medical claims processing code	610433104	Furosemide tablet [TAIYO] 20mg
Drug 6	23_DPC or Receipt Pharmaceutical Information	Electronic medical claims processing code	610453012	Azoselic tablet 60mg
Drug 6	23_DPC or Receipt Pharmaceutical Information	Electronic medical claims processing code	610454048	Diart tablet 60mg
Drug 6	23_DPC or Receipt Pharmaceutical Information	Electronic medical claims processing code	612130071	Eutensin 40mg
Drug 6	23_DPC or Receipt Pharmaceutical Information	Electronic medical claims processing code	612130202	Foliront 40mg
Drug 6	23_DPC or Receipt Pharmaceutical Information	Electronic medical claims processing code	612130217	Furosemide tablet [ISEI] 40mg
Drug 6	23_DPC or Receipt Pharmaceutical Information	Electronic medical claims processing code	612130220	Furosemide tablet [TOWA] 40mg
Drug 6	23_DPC or Receipt Pharmaceutical Information	Electronic medical claims processing code	612130223	Furosemide tablet [MITA] 40mg
Drug 6	23_DPC or Receipt Pharmaceutical Information	Electronic medical claims processing code	612130249	Maoread 40mg
Drug 6	23_DPC or Receipt Pharmaceutical Information	Electronic medical claims processing code	612130280	Lunetoron tablet 1mg
Drug 6	23_DPC or Receipt Pharmaceutical Information	Electronic medical claims processing code	612130313	Arelix 3mg tablet
Drug 6	23_DPC or Receipt Pharmaceutical Information	Electronic medical claims processing code	612130314	Arelix 6mg tablet
Drug 6	23_DPC or Receipt Pharmaceutical Information	Electronic medical claims processing code	612130353	Diart tablet 30mg
Drug 6	23_DPC or Receipt Pharmaceutical Information	Electronic medical claims processing code	620000167	Lasix tablet 20mg
Drug 6	23_DPC or Receipt Pharmaceutical Information	Electronic medical claims processing code	620000168	Lasix tablet 40mg
Drug 6	23_DPC or Receipt Pharmaceutical Information	Electronic medical claims processing code	620000339	Lasix injection 20mg
Drug 6	23_DPC or Receipt Pharmaceutical Information	Electronic medical claims processing code	620000340	Lasix injection 100mg
Drug 6	23_DPC or Receipt Pharmaceutical Information	Electronic medical claims processing code	620003182	Lasix granule 4%
Drug 6	23_DPC or Receipt Pharmaceutical Information	Electronic medical claims processing code	620003238	Furosemide injection [MITA] 20mg
Drug 6	23_DPC or Receipt Pharmaceutical Information	Electronic medical claims processing code	620003439	Tabilon tablet 40mg
Drug 6	23_DPC or Receipt Pharmaceutical Information	Electronic medical claims processing code	620003440	Furosemide tablet [TAIYO] 40mg
Drug 6	23_DPC or Receipt Pharmaceutical Information	Electronic medical claims processing code	620003612	Furosemide tablet 40mg [FUSO]
Drug 6	23_DPC or Receipt Pharmaceutical Information	Electronic medical claims processing code	620003836	Lowpston injection 20mg
Drug 6	23_DPC or Receipt Pharmaceutical Information	Electronic medical claims processing code	620004041	Furosemide tablet 10mg [NP]
Drug 6	23_DPC or Receipt Pharmaceutical Information	Electronic medical claims processing code	620004042	Furosemide tablet 20mg [NP]
Drug 6	23_DPC or Receipt Pharmaceutical Information	Electronic medical claims processing code	620004043	Furosemide tablet 40mg [NP]
Drug 6	23_DPC or Receipt Pharmaceutical Information	Electronic medical claims processing code	620004758	Furosemide injection 20mg [TOWA]
Drug 6	23_DPC or Receipt Pharmaceutical Information	Electronic medical claims processing code	620004759	Furosemide injection 20mg [FUSO]
Drug 6	23_DPC or Receipt Pharmaceutical Information	Electronic medical claims processing code	620005097	Furosemide tablet 40mg [MITA]
Drug 6	23_DPC or Receipt Pharmaceutical Information	Electronic medical claims processing code	620005221	Furosemide injection 20mg syringe [TAIYO] 2ml
Drug 6	23_DPC or Receipt Pharmaceutical Information	Electronic medical claims processing code	620005550	Furosemide tablet 20mg [JG]
Drug 6	23_DPC or Receipt Pharmaceutical Information	Electronic medical claims processing code	620006110	Furosemide tablet 40mg [TOWA]
Drug 6	23_DPC or Receipt Pharmaceutical Information	Electronic medical claims processing code	620007070	Furosemide tablet 40mg [ISEI]
Drug 6	23_DPC or Receipt Pharmaceutical Information	Electronic medical claims processing code	620007103	Maoread tablet 40mg
Drug 6	23_DPC or Receipt Pharmaceutical Information	Electronic medical claims processing code	620007146	Lunetoron tablet 1mg
Drug 6	23_DPC or Receipt Pharmaceutical Information	Electronic medical claims processing code	620007548	Lunetoron injection 0.5mg
Drug 6	23_DPC or Receipt Pharmaceutical Information	Electronic medical claims processing code	620009199	Furosemide injection 20mg [TAIYO]
Drug 6	23_DPC or Receipt Pharmaceutical Information	Electronic medical claims processing code	620269701	Furosemide tablet 20mg [TEVA]
Drug 6	23_DPC or Receipt Pharmaceutical Information	Electronic medical claims processing code	620269703	Furosemide tablet 20mg [TAKEDA TEVA]
Drug 6	23_DPC or Receipt Pharmaceutical Information	Electronic medical claims processing code	620269809	Furosemide tablet 40mg [TEVA]
Drug 6	23_DPC or Receipt Pharmaceutical Information	Electronic medical claims processing code	620269833	Furosemide tablet 40mg [TAKEDA TEVA]
Drug 6	23_DPC or Receipt Pharmaceutical Information	Electronic medical claims processing code	620270101	Eutensin capsule 40mg
Drug 6	23_DPC or Receipt Pharmaceutical Information	Electronic medical claims processing code	620270703	Azosemide tablet 60mg [JG]
Drug 6	23_DPC or Receipt Pharmaceutical Information	Electronic medical claims processing code	620271902	Furosemide injection 20mg [NICHI-IKO]
Drug 6	23_DPC or Receipt Pharmaceutical Information	Electronic medical claims processing code	620272101	Furosemide injection 20mg [TEVA]
Drug 6	23_DPC or Receipt Pharmaceutical Information	Electronic medical claims processing code	620272102	Furosemide injection 20mg [TAKEDA TEVA]
Drug 6	23_DPC or Receipt Pharmaceutical Information	Electronic medical claims processing code	621782401	Furosemide injection 20mg syringe [TEVA] 2ml
Drug 6	23_DPC or Receipt Pharmaceutical Information	Electronic medical claims processing code	622077601	Lasix tablet 10mg
Drug 6	23_DPC or Receipt Pharmaceutical Information	Electronic medical claims processing code	622109301	Furosemide tablet 40mg [JG]
Drug 6	23_DPC or Receipt Pharmaceutical Information	Electronic medical claims processing code	622161501	Daitalic tablet 30mg
Drug 6	23_DPC or Receipt Pharmaceutical Information	Electronic medical claims processing code	622161502	Azosemide tablet 30mg [JG]
Drug 6	23_DPC or Receipt Pharmaceutical Information	Electronic medical claims processing code	622531501	Furosemide injection 20mg [SN]
Drug 6	23_DPC or Receipt Pharmaceutical Information	Electronic medical claims processing code	622531601	Furosemide tablet 20mg [SN]
Drug 6	23_DPC or Receipt Pharmaceutical Information	Electronic medical claims processing code	622531701	Furosemide tablet 40mg [SN]
Drug 6	23_DPC or Receipt Pharmaceutical Information	Electronic medical claims processing code	622548901	Furosemide tablet 10mg [TAKEDA TEVA]
Drug 6	23_DPC or Receipt Pharmaceutical Information	Electronic medical claims processing code	622567401	Furosemide tablet 10mg [SN]
Drug 6	23_DPC or Receipt Pharmaceutical Information	Electronic medical claims processing code	622601001	Torasemide tablet 4mg [KO]
Drug 6	23_DPC or Receipt Pharmaceutical Information	Electronic medical claims processing code	622601101	Torasemide tablet 8mg [KO]
Drug 6	23_DPC or Receipt Pharmaceutical Information	Electronic medical claims processing code	622609402	Torasemide OD tablet 4mg [TE]
Drug 6	23_DPC or Receipt Pharmaceutical Information	Electronic medical claims processing code	622609502	Torasemide OD tablet 8mg [TE]
Drug 6	23_DPC or Receipt Pharmaceutical Information	Electronic medical claims processing code	622721300	Azosemide 60mg tablet
Drug 6	23_DPC or Receipt Pharmaceutical Information	Electronic medical claims processing code	622721400	Azosemide 30mg tablet
Drug 6	23_DPC or Receipt Pharmaceutical Information	Electronic medical claims processing code	640412004	Anfuramide injection 20mg
Drug 6	23_DPC or Receipt Pharmaceutical Information	Electronic medical claims processing code	640433068	Floxaid injection 20mg
Drug 6	23_DPC or Receipt Pharmaceutical Information	Electronic medical claims processing code	642130015	Lunetoron injection 0.5mg

Drug 6	23_DPC or Receipt Pharmaceutical Information	Electronic medical claims processing code	642130019	Arelix 6mg injection
Drug 7	22_Prescription/Injection Order or Administration	YJ Code	2139011D1022	Samsca granule 1%
Drug 7	22_Prescription/Injection Order or Administration	YJ Code	2139011D1MID	Generic name code
Drug 7	22_Prescription/Injection Order or Administration	YJ Code	2139011F1023	Samsca tablet 15mg
Drug 7	22_Prescription/Injection Order or Administration	YJ Code	2139011F1MID	Generic name code
Drug 7	22_Prescription/Injection Order or Administration	YJ Code	2139011F2020	Samsca tablet 7.5mg
Drug 7	22_Prescription/Injection Order or Administration	YJ Code	2139011F2MID	Generic name code
Drug 7	22_Prescription/Injection Order or Administration	YJ Code	2139011F3026	Samsca OD tablet 7.5mg
Drug 7	22_Prescription/Injection Order or Administration	YJ Code	2139011F3MID	Generic name code
Drug 7	22_Prescription/Injection Order or Administration	YJ Code	2139011F4022	Samsca OD tablet 15mg
Drug 7	22_Prescription/Injection Order or Administration	YJ Code	2139011F4MID	Generic name code
Drug 7	22_Prescription/Injection Order or Administration	YJ Code	213901XF2027	Samsca tablet 30mg
Drug 7	22_Prescription/Injection Order or Administration	YJ Code	213901XF2MID	Generic name code
Drug 7	22_Prescription/Injection Order or Administration	YJ Code	21390A2D1027	Samsca granule 1%
Drug 7	22_Prescription/Injection Order or Administration	YJ Code	21390A2D1MID	Generic name code
Drug 7	22_Prescription/Injection Order or Administration	YJ Code	21390A2F1028	Samsca OD tablet 7.5mg
Drug 7	22_Prescription/Injection Order or Administration	YJ Code	21390A2F1MID	Generic name code
Drug 7	22_Prescription/Injection Order or Administration	YJ Code	21390A2F2024	Samsca OD tablet 15mg
Drug 7	22_Prescription/Injection Order or Administration	YJ Code	21390A2F2MID	Generic name code
Drug 7	22_Prescription/Injection Order or Administration	YJ Code	2499012F1022	Samsca tablet 30mg
Drug 7	22_Prescription/Injection Order or Administration	YJ Code	2499012F1MID	Generic name code
Drug 7	22_Prescription/Injection Order or Administration	YJ Code	2499012F2029	Samsca OD tablet 30mg
Drug 7	22_Prescription/Injection Order or Administration	YJ Code	2499012F2MID	Generic name code
Drug 7	22_Prescription/Injection Order or Administration	YJ Code	24990A2F1022	Samsca OD tablet 30mg
Drug 7	22_Prescription/Injection Order or Administration	YJ Code	24990A2F1MID	Generic name code
Drug 7	23_DPC or Receipt Pharmaceutical Information	Electronic medical claims processing code	622007201	Samsca tablet 15mg
Drug 7	23_DPC or Receipt Pharmaceutical Information	Electronic medical claims processing code	622174301	Samsca tablet 7.5mg
Drug 7	23_DPC or Receipt Pharmaceutical Information	Electronic medical claims processing code	622281901	Samsca tablet 30mg
Drug 7	23_DPC or Receipt Pharmaceutical Information	Electronic medical claims processing code	622537701	Samsca granule 1%
Drug 7	23_DPC or Receipt Pharmaceutical Information	Electronic medical claims processing code	622694301	Samsca OD tablet 7.5mg
Drug 7	23_DPC or Receipt Pharmaceutical Information	Electronic medical claims processing code	622694401	Samsca OD tablet 15mg
Drug 7	23_DPC or Receipt Pharmaceutical Information	Electronic medical claims processing code	622694501	Samsca OD tablet 30mg
Drug 8	22_Prescription/Injection Order or Administration	YJ Code	2119406A1025	Amcoral injection 50
Drug 8	22_Prescription/Injection Order or Administration	YJ Code	2119406A1033	Cartonic injection 50mg
Drug 8	22_Prescription/Injection Order or Administration	YJ Code	2119406A1MID	Generic name code
Drug 8	22_Prescription/Injection Order or Administration	YJ Code	2119406A2021	Amcoral injection 100
Drug 8	22_Prescription/Injection Order or Administration	YJ Code	2119406A2030	Cartonic injection 100mg
Drug 8	22_Prescription/Injection Order or Administration	YJ Code	2119406A2MID	Generic name code
Drug 8	22_Prescription/Injection Order or Administration	YJ Code	2119407A1020	Coretec injection 5mg
Drug 8	22_Prescription/Injection Order or Administration	YJ Code	2119407A1MID	Generic name code
Drug 8	22_Prescription/Injection Order or Administration	YJ Code	2119407G1022	Coretec injection SB 9mg
Drug 8	22_Prescription/Injection Order or Administration	YJ Code	2119407G1MID	Generic name code
Drug 8	22_Prescription/Injection Order or Administration	YJ Code	2119408A1024	Milrila injection 10mg
Drug 8	22_Prescription/Injection Order or Administration	YJ Code	2119408A1032	Milrinone injection 10 [KN]
Drug 8	22_Prescription/Injection Order or Administration	YJ Code	2119408A1040	Milrinone injection 10mg [TAKATA]
Drug 8	22_Prescription/Injection Order or Administration	YJ Code	2119408A1059	Milrinone injection 10mg [TAIYO]
Drug 8	22_Prescription/Injection Order or Administration	YJ Code	2119408A1MID	Generic name code
Drug 8	22_Prescription/Injection Order or Administration	YJ Code	2119408A2020	Milrinone injection 10mg [F]
Drug 8	22_Prescription/Injection Order or Administration	YJ Code	2119408A2039	Milrinone injection 10mg [SANDOZ]
Drug 8	22_Prescription/Injection Order or Administration	YJ Code	2119408A2MID	Generic name code
Drug 8	22_Prescription/Injection Order or Administration	YJ Code	2119408A3027	Milrinone injection 22.5mg [F]
Drug 8	22_Prescription/Injection Order or Administration	YJ Code	2119408A3MID	Generic name code
Drug 8	22_Prescription/Injection Order or Administration	YJ Code	2119408G1027	Milrila-K injection 22.5mg
Drug 8	22_Prescription/Injection Order or Administration	YJ Code	2119408G1035	Milrinone injection 22.5mg bag [TAKATA]
Drug 8	22_Prescription/Injection Order or Administration	YJ Code	2119408G1MID	Generic name code
Drug 8	22_Prescription/Injection Order or Administration	YJ Code	2119408G2023	Milrinone injection syringe 10mg [HK]
Drug 8	22_Prescription/Injection Order or Administration	YJ Code	2119408G2MID	Generic name code
Drug 8	23_DPC or Receipt Pharmaceutical Information	Electronic medical claims processing code	620002437	Coretec injection SB 9mg 150ml
Drug 8	23_DPC or Receipt Pharmaceutical Information	Electronic medical claims processing code	620003824	Milrinone injection syringe 10mg [HK] 10ml
Drug 8	23_DPC or Receipt Pharmaceutical Information	Electronic medical claims processing code	620004201	Milrinone injection 10mg [F] 10ml
Drug 8	23_DPC or Receipt Pharmaceutical Information	Electronic medical claims processing code	620004202	Milrinone injection 22.5mg [F] 150ml
Drug 8	23_DPC or Receipt Pharmaceutical Information	Electronic medical claims processing code	620004203	Milrinone injection [KN] 10mg 10ml
Drug 8	23_DPC or Receipt Pharmaceutical Information	Electronic medical claims processing code	620004204	Milrinone injection 10mg [TAKATA] 10ml
Drug 8	23_DPC or Receipt Pharmaceutical Information	Electronic medical claims processing code	620004205	Milrinone injection 22.5mg bag [TAKATA] 150ml
Drug 8	23_DPC or Receipt Pharmaceutical Information	Electronic medical claims processing code	620008233	Milrinone injection 10mg [TAIYO] 10ml
Drug 8	23_DPC or Receipt Pharmaceutical Information	Electronic medical claims processing code	621967601	Milrinone injection 10mg [SANDOZ] 10ml
Drug 8	23_DPC or Receipt Pharmaceutical Information	Electronic medical claims processing code	622750300	Milrinone 10mg 10ml injection
Drug 8	23_DPC or Receipt Pharmaceutical Information	Electronic medical claims processing code	622750400	Milrinone 22.5mg 150m linjection
Drug 8	23_DPC or Receipt Pharmaceutical Information	Electronic medical claims processing code	622750500	Milrinone 22.5mg 150ml kit
Drug 8	23_DPC or Receipt Pharmaceutical Information	Electronic medical claims processing code	640408079	Coretec injection 5mg 5ml
Drug 8	23_DPC or Receipt Pharmaceutical Information	Electronic medical claims processing code	640408133	Milrila injection 10mg 10ml
Drug 8	23_DPC or Receipt Pharmaceutical Information	Electronic medical claims processing code	640421064	Milrila-K injection 22.5mg 150ml
Drug 8	23_DPC or Receipt Pharmaceutical Information	Electronic medical claims processing code	642110135	Amcoral injection 50 0.5% 10ml
Drug 8	23_DPC or Receipt Pharmaceutical Information	Electronic medical claims processing code	642110136	Amcoral injection 100 0.5% 20ml
Drug 8	23_DPC or Receipt Pharmaceutical Information	Electronic medical claims processing code	642110138	Cartonic injection 100mg 0.5% 20ml
Blood test 1, 2 and 3	05_Clinical Laboratory Test Information	JLAC10 Code	4Z271000002200001	Brain natriuretic peptide (BNP), plasma, quantitative value
Blood test 1, 2 and 3	05_Clinical Laboratory Test Information	JLAC10 Code	4Z271000002200601	Brain natriuretic peptide (BNP), plasma, radioimmunoassay (RIA), quantitative value
Blood test 1, 2 and 3	05_Clinical Laboratory Test Information	JLAC10 Code	4Z271000002202301	Brain natriuretic peptide (BNP), plasma, enzyme immunoassay (EIA), quantitative value
Blood test 1, 2 and 3	05_Clinical Laboratory Test Information	JLAC10 Code	4Z271000002202501	Brain natriuretic peptide (BNP), plasma, enzyme immunoassay (EIA), quantitative value
Blood test 1, 2 and 3	05_Clinical Laboratory Test Information	JLAC10 Code	4Z271000002205101	Brain natriuretic peptide (BNP), plasma, chemiluminescent Immunoassay (CLIA), quantitative value
Blood test 1, 2 and 3	05_Clinical Laboratory Test Information	JLAC10 Code	4Z271000002205201	Brain natriuretic peptide (BNP), plasma, chemiluminescent enzyme immunoassay (CLEIA), quantitative value
Blood test 1, 2 and 3	05_Clinical Laboratory Test Information	JLAC10 Code	4Z271000002205301	Brain natriuretic peptide (BNP), plasma, electrochemiluminescence immunoassay (ECLIA), quantitative value
Blood test 1, 2 and 3	05_Clinical Laboratory Test Information	JLAC10 Code	4Z271000002227701	Brain natriuretic peptide (BNP), plasma, visible absorption photometry （other than JSCC、IFCC method）, quantitative value
Blood test 1, 2 and 3	05_Clinical Laboratory Test Information	JLAC10 Code	4Z271000002299801	Brain natriuretic peptide (BNP), plasma, any method, quantitative value
Blood test 1, 2 and 3	05_Clinical Laboratory Test Information	JLAC10 Code	4Z271000002299901	Brain natriuretic peptide (BNP), plasma, other method, quantitative value
Blood test 4, 5 and 6	05_Clinical Laboratory Test Information	JLAC10 Code	4Z272000002200001	N-terminal pro-brain natriuretic peptide (NT-proBNP), plasma, quantitative value
Blood test 4, 5 and 6	05_Clinical Laboratory Test Information	JLAC10 Code	4Z272000002205101	N-terminal pro-brain natriuretic peptide (NT-proBNP), plasma, chemiluminescent Immunoassay (CLIA), quantitative value
Blood test 4, 5 and 6	05_Clinical Laboratory Test Information	JLAC10 Code	4Z272000002205301	N-terminal pro-brain natriuretic peptide (NT-proBNP), plasma, electrochemiluminescence immunoassay (ECLIA), quantitative value
Blood test 4, 5 and 6	05_Clinical Laboratory Test Information	JLAC10 Code	4Z272000002300001	N-terminal pro-brain natriuretic peptide (NT-proBNP), serum, quantitative value
Blood test 4, 5 and 6	05_Clinical Laboratory Test Information	JLAC10 Code	4Z272000002302301	N-terminal pro-brain natriuretic peptide (NT-proBNP), serum, enzyme immunoassay (EIA), quantitative value
Blood test 4, 5 and 6	05_Clinical Laboratory Test Information	JLAC10 Code	4Z272000002304301	N-terminal pro-brain natriuretic peptide (NT-proBNP), serum, fluorescent immunoassay (FIA), quantitative value
Blood test 4, 5 and 6	05_Clinical Laboratory Test Information	JLAC10 Code	4Z272000002305201	N-terminal pro-brain natriuretic peptide (NT-proBNP), serum, chemiluminescent enzyme immunoassay (CLEIA), quantitative value
Blood test 4, 5 and 6	05_Clinical Laboratory Test Information	JLAC10 Code	4Z272000002305301	N-terminal pro-brain natriuretic peptide (NT-proBNP), serum, electrochemiluminescence immunoassay (ECLIA), quantitative value
Medical Practice 1	27_DPC or Receipt Medical Procedure Information	Electronic medical claims processing code	150342470	Intraoperative transesophageal echocardiography continuous monitoring surcharge (cardiac surgery or coronary artery disease/valvular disease)
Medical Practice 1	27_DPC or Receipt Medical Procedure Information	Electronic medical claims processing code	150395670	Intraoperative transesophageal echocardiography continuous monitoring surcharge (catheter-assisted percutaneous cardiac surgery)
Medical Practice 1	27_DPC or Receipt Medical Procedure Information	Electronic medical claims processing code	160072210	Ultrasound examination (tomography) (chest and abdomen)
Medical Practice 1	27_DPC or Receipt Medical Procedure Information	Electronic medical claims processing code	160072510	Ultrasound examination (cardiac ultrasound examination) (trans-thoracic echocardiography)
Medical Practice 1	27_DPC or Receipt Medical Procedure Information	Electronic medical claims processing code	160072610	Ultrasound examination (cardiac ultrasound examination) (M-mode method)
Medical Practice 1	27_DPC or Receipt Medical Procedure Information	Electronic medical claims processing code	160160410	Ultrasound examination (cardiac ultrasound examination) (transesophageal echocardiography)
Medical Practice 1	27_DPC or Receipt Medical Procedure Information	Electronic medical claims processing code	160165010	Ultrasound examination (tomography) (other)
Medical Practice 1	27_DPC or Receipt Medical Procedure Information	Electronic medical claims processing code	160198470	Intracardiac echocardiography surcharge
Medical Practice 1	27_DPC or Receipt Medical Procedure Information	Electronic medical claims processing code	160198810	Ultrasound examination (cardiac ultrasound examination) (stress echocardiography)
Medical Practice 1	27_DPC or Receipt Medical Procedure Information	Electronic medical claims processing code	160218310	Ultrasound examination (tomography) (visiting medical care)

※Up to four diagnoses each can be input into the categories ″comorbidities at the time of hospitalization″ and ″diseases that developed after hospitalization″
