# Supplementary material for: Establishment of reliable identification algorithms for acute heart failure or acute exacerbation of chronic heart failure using clinical data from a medical information database network
Source: Front Cardiovasc Med. 2025 Oct 15;12:1642323. doi: 10.3389/fcvm.2025.1642323 (PMC12568510; doi:10.3389/fcvm.2025.1642323)
Supplement: Supplementary file 2 [file Datasheet1.pdf]

Supplementary Table 2. PPV and sensitivity results for the three medical institutions in the main analysis

Institution	Possible Cases	True positives	False positives	False negatives	PPV (%)	95% confidence interval [Lower limit-Upper limit]	sensitivity (%)	95% confidence interval [Lower limit-Upper limit]
Algorithm of APC								
A	120	42	78	0	35.00	[26.52-44.24]	100.00	-
B	119	26	93	0	21.85	[14.80-30.35]	100.00	-
C	109	18	91	0	16.51	[10.09-24.84]	100.00	-
total	348	86	262	0	24.71	[20.27-29.59]	100.00	-
Algorithm 1								
A	57	28	29	14	49.12	[35.63-62.71]	66.67	[50.45-80.43]
B	50	23	27	3	46.00	[31.81-60.68]	88.46	[69.85-97.55]
C	53	17	36	1	32.08	[19.92-46.32]	94.44	[72.71-99.86]
total	160	68	92	18	42.50	[34.73-50.55]	79.07	[68.95-87.10]
Algorithm 2								
A	58	28	30	14	48.28	[34.95-61.78]	66.67	[50.45-80.43]
B	55	24	31	2	43.64	[30.30-57.68]	92.31	[74.87-99.05]
C	53	17	36	1	32.08	[19.92-46.32]	94.44	[72.71-99.86]
total	166	69	97	17	41.57	[33.98-49.46]	80.23	[70.25-88.04]
Algorithm 3								
A	56	28	28	14	50.00	[36.34-63.66]	66.67	[50.45-80.43]
B	51	23	28	3	45.10	[31.13-59.66]	88.46	[69.85-97.55]
C	50	16	34	2	32.00	[19.52-46.70]	88.89	[65.29-98.62]
total	157	67	90	19	42.68	[34.83-50.81]	77.91	[67.67-86.14]
Algorithm 4								
A	43	25	18	17	58.14	[42.13-72.99]	59.52	[43.28-74.37]
B	39	19	20	7	48.72	[32.42-65.22]	73.08	[52.21-88.43]
C	37	14	23	4	37.84	[22.46-55.24]	77.78	[52.36-93.59]
total	119	58	61	28	48.74	[39.47-58.07]	67.44	[56.48-77.16]
Algorithm 5								
A	33	15	18	27	45.46	[28.11-63.65]	35.71	[21.55-51.97]
B	34	17	17	9	50.00	[32.43-67.57]	65.38	[44.33-82.79]
C	30	13	17	5	43.33	[25.46-62.57]	72.22	[46.52-90.31]
total	97	45	52	41	46.39	[36.20-56.81]	52.33	[41.27-63.21]
Algorithm 6								
A	30	15	15	27	50.00	[31.30-68.70]	35.71	[21.55-51.97]
B	32	15	17	11	46.88	[29.09-65.26]	57.69	[36.92-76.65]
C	26	13	13	5	50.00	[29.93-70.07]	72.22	[46.52-90.31]
total	88	43	45	43	48.86	[38.05-59.75]	50.00	[39.02-60.98]
Algorithm 7								
A	35	25	10	17	71.43	[53.70-85.36]	59.52	[43.28-74.37]
B	23	13	10	13	56.52	[34.49-76.81]	50.00	[29.93-70.07]
C	36	16	20	2	44.44	[27.94-61.90]	88.89	[65.29-98.62]
total	94	54	40	32	57.45	[46.82-67.59]	62.79	[51.70-72.98]
Algorithm 8								
A	6	6	0	36	100.00	[54.07-100.00]	14.29	[5.43-28.54]
B	6	4	2	22	66.67	[22.28-95.67]	15.38	[4.36-34.87]
C	15	11	4	7	73.33	[44.90-92.21]	61.11	[35.75-82.70]
total	27	21	6	65	77.78	[57.74-91.38]	24.42	[15.80-34.87]

Algorithm 9								
A	69	38	31	4	55.07	[42.62-67.08]	90.48	[77.38-97.34]
B	62	23	39	3	37.10	[25.16-50.31]	88.46	[69.85-97.55]
C	58	16	42	2	27.59	[16.66-40.90]	88.89	[65.29-98.62]
total	189	77	112	9	40.74	[33.67-48.11]	89.53	[81.06-95.10]
Algorithm 10								
A	50	32	18	10	64.00	[49.19-77.08]	76.19	[60.55-87.95]
B	56	23	33	3	41.07	[28.10-55.02]	88.46	[69.85-97.55]
C	51	15	36	3	29.41	[17.49-43.83]	83.33	[58.58-96.42]
total	157	70	87	16	44.59	[36.66-52.72]	81.40	[71.55-88.98]
Algorithm 11								
A	45	30	15	12	66.67	[51.05-80.00]	71.43	[55.42-84.28]
B	48	19	29	7	39.58	[25.77-54.73]	73.08	[52.21-88.43]
C	48	15	33	3	31.25	[18.66-46.25]	83.33	[58.58-96.42]
total	141	64	77	22	45.39	[36.99-53.98]	74.42	[63.87-83.22]
Algorithm 12								
A	37	28	9	14	75.68	[58.80-88.23]	66.67	[50.45-80.43]
B	41	19	22	7	46.34	[30.66-62.58]	73.08	[52.21-88.43]
C	33	12	21	6	36.36	[20.40-54.88]	66.67	[40.99-86.66]
total	111	59	52	27	53.15	[43.45-62.69]	68.60	[57.70-78.19]
Algorithm 13								
A	50	32	18	10	64.00	[49.19-77.08]	76.19	[60.55-87.95]
B	51	20	31	6	39.22	[25.84-53.89]	76.92	[56.35-91.03]
C	50	15	35	3	30.00	[17.86-44.61]	83.33	[58.58-96.42]
total	151	67	84	19	44.37	[36.30-52.67]	77.91	[67.67-86.14]
Algorithm 14								
A	7	4	3	38	57.14	[18.41-90.10]	9.52	[2.66-22.62]
B	15	8	7	18	53.33	[26.59-78.73]	30.77	[14.33-51.79]
C	7	5	2	13	71.43	[29.04-96.33]	27.78	[9.69-53.48]
total	29	17	12	69	58.62	[38.94-76.48]	19.77	[11.96-29.75]
Algorithm 15								
A	27	15	12	27	55.56	[35.33-74.52]	35.71	[21.55-51.97]
B	32	16	16	10	50.00	[31.89-68.11]	61.54	[40.57-79.77]
C	27	11	16	7	40.74	[22.39-61.20]	61.11	[35.75-82.70]
total	86	42	44	44	48.84	[37.90-59.86]	48.84	[37.90-59.86]
Algorithm 16								
A	27	15	12	27	55.56	[35.33-74.52]	35.71	[21.55-51.97]
B	30	14	16	12	46.67	[28.34-65.67]	53.85	[33.37-73.41]
C	23	11	12	7	47.83	[26.82-69.41]	61.11	[35.75-82.70]
total	80	40	40	46	50.00	[38.60-61.40]	46.51	[35.68-57.59]
Algorithm 17								
A	35	27	8	15	77.14	[59.86-89.58]	64.29	[48.03-78.45]
B	28	13	15	13	46.43	[27.51-66.13]	50.00	[29.93-70.07]
C	32	15	17	3	46.88	[29.09-65.26]	83.33	[58.58-96.42]
total	95	55	40	31	57.89	[47.33-67.96]	63.95	[52.88-74.03]
Algorithm 18								
A	8	7	1	35	87.50	[47.35-99.68]	16.67	[6.97-31.36]
B	6	4	2	22	66.67	[22.28-95.67]	15.38	[4.36-34.87]
C	15	11	4	7	73.33	[44.90-92.21]	61.11	[35.75-82.70]
total	29	22	7	64	75.86	[56.46-89.70]	25.58	[16.78-36.13]

Supplementary Table 3. PPV and sensitivity results for the three medical institutions in the sensitivity analysis

Institution	Possible Cases	True positives	False positives	False negatives	PPV (%)	95% confidence interval [Lower limit-Upper limit]	sensitivity (%)	95% confidence interval [Lower limit-Upper limit]
Algorithm of APC								
A	120	46	74	0	38.33	[29.61-47.65]	100.00	-
B	119	28	91	0	23.53	[16.24-32.18]	100.00	-
C	109	21	88	0	19.27	[12.34-27.93]	100.00	-
total	348	95	253	0	27.30	[22.69-32.30]	100.00	-
Algorithm 1								
A	57	32	25	14	56.14	[42.36-69.26]	69.57	[54.25-82.26]
B	50	23	27	5	46.00	[31.81-60.68]	82.14	[63.11-93.94]
C	53	20	33	1	37.74	[24.79-52.11]	95.24	[76.18-99.88]
total	160	75	85	20	46.88	[38.95-54.92]	78.95	[69.38-86.64]
Algorithm 2								
A	58	32	26	14	55.17	[41.54-68.26]	69.57	[54.25-82.26]
B	55	24	31	4	43.64	[30.30-57.68]	85.71	[67.33-95.97]
C	53	20	33	1	37.74	[24.79-52.11]	95.24	[76.18-99.88]
total	166	76	90	19	45.78	[38.04-53.68]	80.00	[70.54-87.51]
Algorithm 3								
A	56	31	25	15	55.36	[41.47-68.66]	67.39	[51.98-80.47]
B	51	23	28	5	45.10	[31.13-59.66]	82.14	[63.11-93.94]
C	50	19	31	2	38.00	[24.65-52.83]	90.48	[69.62-98.83]
total	157	73	84	22	46.50	[38.51-54.62]	76.84	[67.06-84.88]
Algorithm 4								
A	43	28	15	18	65.12	[49.07-78.99]	60.87	[45.37-74.91]
B	39	19	20	9	48.72	[32.42-65.22]	67.86	[47.65-84.12]
C	37	17	20	4	45.95	[29.49-63.08]	80.95	[58.09-94.55]
total	119	64	55	31	53.78	[44.41-62.96]	67.37	[56.98-76.64]
Algorithm 5								
A	33	18	15	28	54.55	[36.35-71.89]	39.13	[25.09-54.63]
B	34	17	17	11	50.00	[32.43-67.57]	60.71	[40.58-78.50]
C	30	15	15	6	50.00	[31.30-68.70]	71.43	[47.82-88.72]
total	97	50	47	45	51.55	[41.18-61.82]	52.63	[42.12-62.97]
Algorithm 6								
A	30	18	12	28	60.00	[40.60-77.34]	39.13	[25.09-54.63]
B	32	15	17	13	46.88	[29.09-65.26]	53.57	[33.87-72.49]
C	26	14	12	7	53.85	[33.37-73.41]	66.67	[43.03-85.41]
total	88	47	41	48	53.41	[42.46-64.12]	49.47	[39.05-59.93]
Algorithm 7								
A	35	26	9	20	74.29	[56.74-87.51]	56.52	[41.11-71.07]
B	23	13	10	15	56.52	[34.49-76.81]	46.43	[27.51-66.13]
C	36	19	17	2	52.78	[35.49-69.59]	90.48	[69.62-98.83]
total	94	58	36	37	61.70	[51.10-71.54]	61.05	[50.50-70.89]
Algorithm 8								
A	6	6	0	40	100.00	[54.07-100.00]	13.04	[4.94-26.26]
B	6	4	2	24	66.67	[22.28-95.67]	14.29	[4.03-32.67]
C	15	12	3	9	80.00	[51.91-95.67]	57.14	[34.02-78.18]
total	27	22	5	73	81.48	[61.92-93.70]	23.16	[15.12-32.94]

Algorithm 9								
A	69	40	29	6	57.97	[45.48-69.76]	86.96	[73.74-95.06]
B	62	25	37	3	40.32	[28.05-53.55]	89.29	[71.77-97.73]
C	58	19	39	2	32.76	[21.01-46.34]	90.48	[69.62-98.83]
total	189	84	105	11	44.44	[37.23-51.83]	88.42	[80.23-94.08]
Algorithm 10								
A	50	33	17	13	66.00	[51.23-78.79]	71.74	[56.54-84.01]
B	56	25	31	3	44.64	[31.34-58.53]	89.29	[71.77-97.73]
C	51	18	33	3	35.29	[22.43-49.93]	85.71	[63.66-96.95]
total	157	76	81	19	48.41	[40.37-56.51]	80.00	[70.54-87.51]
Algorithm 11								
A	45	31	14	15	68.89	[53.35-81.83]	67.39	[51.98-80.47]
B	48	20	28	8	41.67	[27.61-56.79]	71.43	[51.33-86.78]
C	48	17	31	4	35.42	[22.16-50.54]	80.95	[58.09-94.55]
total	141	68	73	27	48.23	[39.74-56.79]	71.58	[61.40-80.36]
Algorithm 12								
A	37	29	8	17	78.38	[61.79-90.17]	63.04	[47.55-76.79]
B	41	20	21	8	48.78	[32.88-64.87]	71.43	[51.33-86.78]
C	33	15	18	6	45.46	[28.11-63.65]	71.43	[47.82-88.72]
total	111	64	47	31	57.66	[47.92-66.98]	67.37	[56.98-76.64]
Algorithm 13								
A	50	33	17	13	66.00	[51.23-78.79]	71.74	[56.54-84.01]
B	51	21	30	7	41.18	[27.58-55.83]	75.00	[55.13-89.31]
C	50	18	32	3	36.00	[22.92-50.81]	85.71	[63.66-96.95]
total	151	72	79	23	47.68	[39.50-55.96]	75.79	[65.92-83.99]
Algorithm 14								
A	7	4	3	42	57.14	[18.41-90.10]	8.70	[2.42-20.79]
B	15	8	7	20	53.33	[26.59-78.73]	28.57	[13.22-48.67]
C	7	5	2	16	71.43	[29.04-96.33]	23.81	[8.22-47.17]
total	29	17	12	78	58.62	[38.94-76.48]	17.89	[10.78-27.10]
Algorithm 15								
A	27	16	11	30	59.26	[38.80-77.61]	34.78	[21.35-50.25]
B	32	18	14	10	56.25	[37.66-73.64]	64.29	[44.07-81.36]
C	27	13	14	8	48.15	[28.67-68.05]	61.90	[38.44-81.89]
total	86	47	39	48	54.65	[43.55-65.42]	49.47	[39.05-59.93]
Algorithm 16								
A	27	16	11	30	59.26	[38.80-77.61]	34.78	[21.35-50.25]
B	30	16	14	12	53.33	[34.33-71.66]	57.14	[37.18-75.54]
C	23	12	11	9	52.17	[30.59-73.18]	57.14	[34.02-78.18]
total	80	44	36	51	55.00	[43.47-66.15]	46.32	[36.02-56.85]
Algorithm 17								
A	35	27	8	19	77.14	[59.86-89.58]	58.70	[43.23-73.00]
B	28	13	15	15	46.43	[27.51-66.13]	46.43	[27.51-66.13]
C	32	18	14	3	56.25	[37.66-73.64]	85.71	[63.66-96.95]
total	95	58	37	37	61.05	[50.50-70.89]	61.05	[50.50-70.89]
Algorithm 18								
A	8	7	1	39	87.50	[47.35-99.68]	15.22	[6.34-28.87]
B	6	4	2	24	66.67	[22.28-95.67]	14.29	[4.03-32.67]
C	15	12	3	9	80.00	[51.91-95.67]	57.14	[34.02-78.18]
total	29	23	6	72	79.31	[60.28-92.01]	24.21	[16.01-34.08]

Supplementary Table 4. NPV and specificity results for the three medical institutions in the primary analysis

Institution	N	Possible Cases	Applicable to the Algorithm		Not applicable to the Algorithm		NPV (%)	95% confidence interval [Lower limit-Upper limit]	Specificity (%)	95% confidence interval [Lower limit-Upper limit]
			True positives	False positives	False negatives	True negatives				
Algorithm of APC										
A	30,442	120	42	78	0	30,322	100.00	-	99.74	[99.68-99.80]
B	36,385	119	26	93	0	36,266	100.00	-	99.74	[99.69-99.79]
C	125,062	109	18	91	0	124,953	100.00	-	99.93	[99.91-99.94]
total	191,889	348	86	262	0	191,541	100.00	-	99.86	[99.85-99.88]
Algorithm 1										
A	30,442	57	28	29	14	30,371	99.95	[99.92-99.97]	99.90	[99.86-99.94]
B	36,385	50	23	27	3	36,332	99.99	[99.98-100.00]	99.93	[99.89-99.95]
C	125,062	53	17	36	1	125,008	100.00	[100.00-100.00]	99.97	[99.96-99.98]
total	191,889	160	68	92	18	191,711	99.99	[99.99-99.99]	99.95	[99.94-99.96]
Algorithm 2										
A	30,442	58	28	30	14	30,370	99.95	[99.92-99.97]	99.90	[99.86-99.93]
B	36,385	55	24	31	2	36,328	99.99	[99.98-100.00]	99.91	[99.88-99.94]
C	125,062	53	17	36	1	125,008	100.00	[100.00-100.00]	99.97	[99.96-99.98]
total	191,889	166	69	97	17	191,706	99.99	[99.99-99.99]	99.95	[99.94-99.96]
Algorithm 3										
A	30,442	56	28	28	14	30,372	99.95	[99.92-99.97]	99.91	[99.87-99.94]
B	36,385	51	23	28	3	36,331	99.99	[99.98-100.00]	99.92	[99.89-99.95]
C	125,062	50	16	34	2	125,010	100.00	[99.99-100.00]	99.97	[99.96-99.98]
total	191,889	157	67	90	19	191,713	99.99	[99.98-99.99]	99.95	[99.94-99.96]
Algorithm 4										
A	30,442	43	25	18	17	30,382	99.94	[99.91-99.97]	99.94	[99.91-99.96]
B	36,385	39	19	20	7	36,339	99.98	[99.96-99.99]	99.94	[99.92-99.97]
C	125,062	37	14	23	4	125,021	100.00	[99.99-100.00]	99.98	[99.97-99.99]
total	191,889	119	58	61	28	191,742	99.99	[99.98-99.99]	99.97	[99.96-99.98]
Algorithm 5										
A	30,442	33	15	18	27	30,382	99.91	[99.87-99.94]	99.94	[99.91-99.96]
B	36,385	34	17	17	9	36,342	99.98	[99.95-99.99]	99.95	[99.93-99.97]
C	125,062	30	13	17	5	125,027	100.00	[99.99-100.00]	99.99	[99.98-99.99]
total	191,889	97	45	52	41	191,751	99.98	[99.97-99.98]	99.97	[99.96-99.98]
Algorithm 6										
A	30,442	30	15	15	27	30,385	99.91	[99.87-99.94]	99.95	[99.92-99.97]
B	36,385	32	15	17	11	36,342	99.97	[99.95-99.98]	99.95	[99.93-99.97]
C	125,062	26	13	13	5	125,031	100.00	[99.99-100.00]	99.99	[99.98-99.99]
total	191,889	88	43	45	43	191,758	99.98	[99.97-99.98]	99.98	[99.97-99.98]
Algorithm 7										
A	30,442	35	25	10	17	30,390	99.94	[99.91-99.97]	99.97	[99.94-99.98]
B	36,385	23	13	10	13	36,349	99.96	[99.94-99.98]	99.97	[99.95-99.99]
C	125,062	36	16	20	2	125,024	100.00	[99.99-100.00]	99.98	[99.98-99.99]
total	191,889	94	54	40	32	191,763	99.98	[99.98-99.99]	99.98	[99.97-99.99]
Algorithm 8										
A	30,442	6	6	0	36	30,400	99.88	[99.84-99.92]	100.00	[99.99-100.00]
B	36,385	6	4	2	22	36,357	99.94	[99.91-99.96]	99.99	[99.98-100.00]
C	125,062	15	11	4	7	125,040	99.99	[99.99-100.00]	100.00	[99.99-100.00]
total	191,889	27	21	6	65	191,797	99.97	[99.96-99.97]	100.00	[99.99-100.00]

Algorithm 9										
A	30,442	69	38	31	4	30,369	99.99	[99.97-100.00]	99.90	[99.86-99.93]
B	36,385	62	23	39	3	36,320	99.99	[99.98-100.00]	99.89	[99.85-99.92]
C	125,062	58	16	42	2	125,002	100.00	[99.99-100.00]	99.97	[99.95-99.98]
total	191,889	189	77	112	9	191,691	100.00	[99.99-100.00]	99.94	[99.93-99.95]
Algorithm 10										
A	30,442	50	32	18	10	30,382	99.97	[99.94-99.98]	99.94	[99.91-99.96]
B	36,385	56	23	33	3	36,326	99.99	[99.98-100.00]	99.91	[99.87-99.94]
C	125,062	51	15	36	3	125,008	100.00	[99.99-100.00]	99.97	[99.96-99.98]
total	191,889	157	70	87	16	191,716	99.99	[99.99-100.00]	99.95	[99.94-99.96]
Algorithm 11										
A	30,442	45	30	15	12	30,385	99.96	[99.93-99.98]	99.95	[99.92-99.97]
B	36,385	48	19	29	7	36,330	99.98	[99.96-99.99]	99.92	[99.89-99.95]
C	125,062	48	15	33	3	125,011	100.00	[99.99-100.00]	99.97	[99.96-99.98]
total	191,889	141	64	77	22	191,726	99.99	[99.98-99.99]	99.96	[99.95-99.97]
Algorithm 12										
A	30,442	37	28	9	14	30,391	99.95	[99.92-99.97]	99.97	[99.94-99.99]
B	36,385	41	19	22	7	36,337	99.98	[99.96-99.99]	99.94	[99.91-99.96]
C	125,062	33	12	21	6	125,023	100.00	[99.99-100.00]	99.98	[99.97-99.99]
total	191,889	111	59	52	27	191,751	99.99	[99.98-99.99]	99.97	[99.96-99.98]
Algorithm 13										
A	30,442	50	32	18	10	30,382	99.97	[99.94-99.98]	99.94	[99.91-99.96]
B	36,385	51	20	31	6	36,328	99.98	[99.96-99.99]	99.91	[99.88-99.94]
C	125,062	50	15	35	3	125,009	100.00	[99.99-100.00]	99.97	[99.96-99.98]
total	191,889	151	67	84	19	191,719	99.99	[99.98-99.99]	99.96	[99.95-99.97]
Algorithm 14										
A	30,442	7	4	3	38	30,397	99.88	[99.83-99.91]	99.99	[99.97-100.00]
B	36,385	15	8	7	18	36,352	99.95	[99.92-99.97]	99.98	[99.96-99.99]
C	125,062	7	5	2	13	125,042	99.99	[99.98-99.99]	100.00	[99.99-100.00]
total	191,889	29	17	12	69	191,791	99.96	[99.95-99.97]	99.99	[99.99-100.00]
Algorithm 15										
A	30,442	27	15	12	27	30,388	99.91	[99.87-99.94]	99.96	[99.93-99.98]
B	36,385	32	16	16	10	36,343	99.97	[99.95-99.99]	99.96	[99.93-99.97]
C	125,062	27	11	16	7	125,028	99.99	[99.99-100.00]	99.99	[99.98-99.99]
total	191,889	86	42	44	44	191,759	99.98	[99.97-99.98]	99.98	[99.97-99.98]
Algorithm 16										
A	30,442	27	15	12	27	30,388	99.91	[99.87-99.94]	99.96	[99.93-99.98]
B	36,385	30	14	16	12	36,343	99.97	[99.94-99.98]	99.96	[99.93-99.97]
C	125,062	23	11	12	7	125,032	99.99	[99.99-100.00]	99.99	[99.98-100.00]
total	191,889	80	40	40	46	191,763	99.98	[99.97-99.98]	99.98	[99.97-99.99]
Algorithm 17										
A	30,442	35	27	8	15	30,392	99.95	[99.92-99.97]	99.97	[99.95-99.99]
B	36,385	28	13	15	13	36,344	99.96	[99.94-99.98]	99.96	[99.93-99.98]
C	125,062	32	15	17	3	125,027	100.00	[99.99-100.00]	99.99	[99.98-99.99]
total	191,889	95	55	40	31	191,763	99.98	[99.98-99.99]	99.98	[99.97-99.99]
Algorithm 18										
A	30,442	8	7	1	35	30,399	99.88	[99.84-99.92]	100.00	[99.98-100.00]
B	36,385	6	4	2	22	36,357	99.94	[99.91-99.96]	99.99	[99.98-100.00]
C	125,062	15	11	4	7	125,040	99.99	[99.99-100.00]	100.00	[99.99-100.00]
total	191,889	29	22	7	64	191,796	99.97	[99.96-99.97]	100.00	[99.99-100.00]

Supplementary Table 5. NPV and specificity results for the three medical institutions in the sensitive analysis

Institution	N	Possible Cases	Applicable to the Algorithm		Not applicable to the Algorithm		NPV (%)	95% confidence interval [Lower limit-Upper limit]	Specificity (%)	95% confidence interval [Lower limit-Upper limit]
			True positives	False positives	False negatives	True negatives				
Algorithm of APC										
A	30,442	120	46	74	0	30,322	100.00	-	99.76	[99.69-99.81]
B	36,385	119	28	91	0	36,266	100.00	-	99.75	[99.69-99.80]
C	125,062	109	21	88	0	124,953	100.00	-	99.93	[99.91-99.94]
total	191,889	348	95	253	0	191,541	100.00	-	99.87	[99.85-99.88]
Algorithm 1										
A	30,442	57	32	25	14	30,371	99.95	[99.92-99.97]	99.92	[99.88-99.95]
B	36,385	50	23	27	5	36,330	99.99	[99.97-100.00]	99.93	[99.89-99.95]
C	125,062	53	20	33	1	125,008	100.00	[100.00-100.00]	99.97	[99.96-99.98]
total	191,889	160	75	85	20	191,709	99.99	[99.98-99.99]	99.96	[99.95-99.96]
Algorithm 2										
A	30,442	58	32	26	14	30,370	99.95	[99.92-99.97]	99.91	[99.87-99.94]
B	36,385	55	24	31	4	36,326	99.99	[99.97-100.00]	99.91	[99.88-99.94]
C	125,062	53	20	33	1	125,008	100.00	[100.00-100.00]	99.97	[99.96-99.98]
total	191,889	166	76	90	19	191,704	99.99	[99.98-99.99]	99.95	[99.94-99.96]
Algorithm 3										
A	30,442	56	31	25	15	30,371	99.95	[99.92-99.97]	99.92	[99.88-99.95]
B	36,385	51	23	28	5	36,329	99.99	[99.97-100.00]	99.92	[99.89-99.95]
C	125,062	50	19	31	2	125,010	100.00	[99.99-100.00]	99.98	[99.96-99.98]
total	191,889	157	73	84	22	191,710	99.99	[99.98-99.99]	99.96	[99.95-99.97]
Algorithm 4										
A	30,442	43	28	15	18	30,381	99.94	[99.91-99.96]	99.95	[99.92-99.97]
B	36,385	39	19	20	9	36,337	99.98	[99.95-99.99]	99.94	[99.92-99.97]
C	125,062	37	17	20	4	125,021	100.00	[99.99-100.00]	99.98	[99.98-99.99]
total	191,889	119	64	55	31	191,739	99.98	[99.98-99.99]	99.97	[99.96-99.98]
Algorithm 5										
A	30,442	33	18	15	28	30,381	99.91	[99.87-99.94]	99.95	[99.92-99.97]
B	36,385	34	17	17	11	36,340	99.97	[99.95-99.98]	99.95	[99.93-99.97]
C	125,062	30	15	15	6	125,026	100.00	[99.99-100.00]	99.99	[99.98-99.99]
total	191,889	97	50	47	45	191,747	99.98	[99.97-99.98]	99.98	[99.97-99.98]
Algorithm 6										
A	30,442	30	18	12	28	30,384	99.91	[99.87-99.94]	99.96	[99.93-99.98]
B	36,385	32	15	17	13	36,340	99.96	[99.94-99.98]	99.95	[99.93-99.97]
C	125,062	26	14	12	7	125,029	99.99	[99.99-100.00]	99.99	[99.98-100.00]
total	191,889	88	47	41	48	191,753	99.97	[99.97-99.98]	99.98	[99.97-99.98]
Algorithm 7										
A	30,442	35	26	9	20	30,387	99.93	[99.90-99.96]	99.97	[99.94-99.99]
B	36,385	23	13	10	15	36,347	99.96	[99.93-99.98]	99.97	[99.95-99.99]
C	125,062	36	19	17	2	125,024	100.00	[99.99-100.00]	99.99	[99.98-99.99]
total	191,889	94	58	36	37	191,758	99.98	[99.97-99.99]	99.98	[99.97-99.99]
Algorithm 8										
A	30,442	6	6	0	40	30,396	99.87	[99.82-99.91]	100.00	[99.99-100.00]
B	36,385	6	4	2	24	36,355	99.93	[99.90-99.96]	99.99	[99.98-100.00]
C	125,062	15	12	3	9	125,038	99.99	[99.99-100.00]	100.00	[99.99-100.00]
total	191,889	27	22	5	73	191,789	99.96	[99.95-99.97]	100.00	[99.99-100.00]

Algorithm 9										
A	30,442	69	40	29	6	30,367	99.98	[99.96-99.99]	99.90	[99.86-99.94]
B	36,385	62	25	37	3	36,320	99.99	[99.98-100.00]	99.90	[99.86-99.93]
C	125,062	58	19	39	2	125,002	100.00	[99.99-100.00]	99.97	[99.96-99.98]
total	191,889	189	84	105	11	191,689	99.99	[99.99-100.00]	99.95	[99.93-99.96]
Algorithm 10										
A	30,442	50	33	17	13	30,379	99.96	[99.93-99.98]	99.94	[99.91-99.97]
B	36,385	56	25	31	3	36,326	99.99	[99.98-100.00]	99.91	[99.88-99.94]
C	125,062	51	18	33	3	125,008	100.00	[99.99-100.00]	99.97	[99.96-99.98]
total	191,889	157	76	81	19	191,713	99.99	[99.98-99.99]	99.96	[99.95-99.97]
Algorithm 11										
A	30,442	45	31	14	15	30,382	99.95	[99.92-99.97]	99.95	[99.92-99.97]
B	36,385	48	20	28	8	36,329	99.98	[99.96-99.99]	99.92	[99.89-99.95]
C	125,062	48	17	31	4	125,010	100.00	[99.99-100.00]	99.98	[99.96-99.98]
total	191,889	141	68	73	27	191,721	99.99	[99.98-99.99]	99.96	[99.95-99.97]
Algorithm 12										
A	30,442	37	29	8	17	30,388	99.94	[99.91-99.97]	99.97	[99.95-99.99]
B	36,385	41	20	21	8	36,336	99.98	[99.96-99.99]	99.94	[99.91-99.96]
C	125,062	33	15	18	6	125,023	100.00	[99.99-100.00]	99.99	[99.98-99.99]
total	191,889	111	64	47	31	191,747	99.98	[99.98-99.99]	99.98	[99.97-99.98]
Algorithm 13										
A	30,442	50	33	17	13	30,379	99.96	[99.93-99.98]	99.94	[99.91-99.97]
B	36,385	51	21	30	7	36,327	99.98	[99.96-99.99]	99.92	[99.88-99.94]
C	125,062	50	18	32	3	125,009	100.00	[99.99-100.00]	99.97	[99.96-99.98]
total	191,889	151	72	79	23	191,715	99.99	[99.98-99.99]	99.96	[99.95-99.97]
Algorithm 14										
A	30,442	7	4	3	42	30,393	99.86	[99.81-99.90]	99.99	[99.97-100.00]
B	36,385	15	8	7	20	36,350	99.95	[99.92-99.97]	99.98	[99.96-99.99]
C	125,062	7	5	2	16	125,039	99.99	[99.98-99.99]	100.00	[99.99-100.00]
total	191,889	29	17	12	78	191,782	99.96	[99.95-99.97]	99.99	[99.99-100.00]
Algorithm 15										
A	30,442	27	16	11	30	30,385	99.90	[99.86-99.93]	99.96	[99.94-99.98]
B	36,385	32	18	14	10	36,343	99.97	[99.95-99.99]	99.96	[99.94-99.98]
C	125,062	27	13	14	8	125,027	99.99	[99.99-100.00]	99.99	[99.98-99.99]
total	191,889	86	47	39	48	191,755	99.97	[99.97-99.98]	99.98	[99.97-99.99]
Algorithm 16										
A	30,442	27	16	11	30	30,385	99.90	[99.86-99.93]	99.96	[99.94-99.98]
B	36,385	30	16	14	12	36,343	99.97	[99.94-99.98]	99.96	[99.94-99.98]
C	125,062	23	12	11	9	125,030	99.99	[99.99-100.00]	99.99	[99.98-100.00]
total	191,889	80	44	36	51	191,758	99.97	[99.97-99.98]	99.98	[99.97-99.99]
Algorithm 17										
A	30,442	35	27	8	19	30,388	99.94	[99.90-99.96]	99.97	[99.95-99.99]
B	36,385	28	13	15	15	36,342	99.96	[99.93-99.98]	99.96	[99.93-99.98]
C	125,062	32	18	14	3	125,027	100.00	[99.99-100.00]	99.99	[99.98-99.99]
total	191,889	95	58	37	37	191,757	99.98	[99.97-99.99]	99.98	[99.97-99.99]
Algorithm 18										
A	30,442	8	7	1	39	30,395	99.87	[99.82-99.91]	100.00	[99.98-100.00]
B	36,385	6	4	2	24	36,355	99.93	[99.90-99.96]	99.99	[99.98-100.00]
C	125,062	15	12	3	9	125,038	99.99	[99.99-100.00]	100.00	[99.99-100.00]
total	191,889	29	23	6	72	191,788	99.96	[99.95-99.97]	100.00	[99.99-100.00]
